# Electromagnetohydrodynamic bioconvective flow of binary fluid containing nanoparticles and gyrotactic microorganisms through a stratified stretching sheet

**DOI:** 10.1038/s41598-021-02320-0

**Published:** 2021-11-30

**Authors:** Abdullah Dawar, Anwar Saeed, Saeed Islam, Zahir Shah, Wiyada Kumam, Poom Kumam

**Affiliations:** 1 Department of Mathematics, Abdul Wali Khan University, Mardan, 23200 Khyber Pakhtunkhwa Pakistan; 2 Center of Excellence in Theoretical and Computational Science (TaCS-CoE), Faculty of Science, King Mongkut’s University of Technology Thonburi (KMUTT), 126 Pracha Uthit Rd., Bang Mod, Thung Khru, Bangkok, 10140 Thailand; 3 Department of Mathematical Sciences, University of Lakki Marwat, Lakki Marwat, Khyber Pakhtunkhwa, 28420 Pakistan; 4 Applied Mathematics for Science and Engineering Research Unit (AMSERU), Program in Applied Statistics, Department of Mathematics and Computer Science, Faculty of Science and Technology, Rajamangala University of Technology Thanyaburi, Thanyaburi, Pathumthani, 12110 Thailand; 5 Department of Medical Research, China Medical University Hospital, China Medical University, Taichung, 40402 Taiwan

**Keywords:** Engineering, Mathematics and computing

## Abstract

Bioconvection has recently been the subject of dispute in a number of biotechnological fields that depend on fluids and their physical properties. When mixed nanofluids are subjected to heat and mass transmission, the process of bioconvection occurs. This attempt conveys the theoretical analysis of two-dimensional electrically conducting and magnetically susceptible binary fluid containing nanoparticles and gyrotactic microorganisms past a stratified stretching surface. Furthermore binary chemical reaction, thermal radiation, and activation energy are taken into assumptions. The analytical solution based on HAM has been performed. The convergence of HAM is presented with the help of figures. The present study is compared with previously published results and has established an excessive agreement which validate the present study. It is perceived that the presence and absence of an electric field influences the variations in fluid velocities due to presence of magnetic field. The micropolar constant heightens the velocity and microrotation of the fluid flow. The buoyancy parameter and bioconvection Rayleigh number diminish the velocity function while these parameters show dual impact on microrotation function. The skin friction and couple stress escalates with the increasing buoyancy ratio parameter and bioconvection Rayleigh number.

## Introduction

The expression "nanofluid" refers to the suspended nanoparticles that maximize the combined heat and mass transfer phenomena within a typical fluid. Nanoparticles have fascinated the researchers’ interest in today’s modern era due to their significant importance in the fields of electronics, food science, biosensors, biomedicine, and mechanical engineering. Additionally, the movement of respective nanoparticles in designated structures is strongly dependent on the elementary concepts of cancer treatment, selective drug delivery, chemotherapy, fermentation science, and nano-medicine. It is a well-established fact that the fluids flowing through microchannels in cooling and heating systems are entirely dependent on the heat transfer particles produced by nanofluids. As a result, nanofluid dynamics is a critical term to grasp for all fields concerned with nonmaterial suspensions in some manner in order to achieve optimum productivity. Choi^1^ pioneered the concept of nanoparticles with improved thermophysical properties, which was subsequently expanded by a number of scientists. Buongiorno^2^ defined the seven slip mechanisms in nanoparticle movement, focusing on Brownian motion and thermophoresis effects. Hsiao^3^ examined mixed convection and slip flow in flow of nanofluid configured by a stretched surface in the existence of both electrical and magnetic field aspects. Turkyilmazoglu^4^ presented the heat transformer of several nanofluids containing Ag, Al_2_O_3_, CuO, Cu, and TiO_2_ nanoparticles through a plane wall jet. Hsiao^5^ probed the hydromagnetic nanofluids flow heat and mass transfer through a stretching sheet with magnetic and viscous dissipation effects. In order to investigate the thermophoresis and Brownian movement of the nanoparticles, Ahmed et al.^6^ used the Buongiorno's model for the stagnation point Maxwell nanofluid flow past a rotating disk. Sandeep and Animasaun^7^ probed the enhanced thermal transmissions of electrically conducting water based nanofluids containing aluminum alloy nanoparticles with magnetic field impact. The effect of radiation thermal transmission on nanofluid between two pipes with horizontal magnetic field is investigated by Sheikholeslami et al.^8^. Shahzadi and Nadeem^9^ presented an effective mathematical model for the blood based peristaltic flow containing two different types of nanoparticles past a porous material with velocity slip conditions and magnetic impact. Sandeep et al.^10^ addressed the water based nanofluids containing magnetite nanoparticles. Sheikholeslami and Bhatti^11^ investigated the heat transmission in a nanofluid over a porous semi-annulus with magnetic field. Raza et al.^12^ deliberated the influence magnetic field on Casson fluid flow containing suspended nanoparticles past a nonlinear permeable surface with velocity slip condition. Siavashi et al.^13^ offered the mixed convection flow of power-law nanofluid containing CuO nanoparticles in a porous enclosure. In addition, the related studies are mentioned in^14–19^.

The activation energy is described as the least energy needed to initiate a chemical reaction. Spontaneous reactions are chemical reactions that require lower activation energy. Nuclear reactions accompanying both fusion and fission of nuclei are crucial, but they necessitate greater activation energy. The importance of activation energy has a significant impact on nanoparticle movement in simple carrier fluids. Chemical engineering, food manufacturing, and the mechanics of oil water emulsions all have a strong demand for activation energy^20–22^. Makinde et al.^23^ analyzed the chemically reactive and thermally radiative unsteady fluid flow over a porous plate. Khan et al.^24^ presented the features of activation energy and entropy generation in Carreau-Yasuda fluid past an extending sheet with magnetic field. The significance of activation energy on an electrically conducting magnetized third grade fluid containing gyrotactic microorganisms over a stretching sheet was probed by Chu et al.^25^. Moreover, the related studies can be found in^26–35^.

Bioconvection has recently been the subject of dispute in a number of biotechnological fields that depend on fluids and their physical properties. When mixed nanofluids are subjected to heat and mass transmission, the process of bioconvection occurs. Kuznetsov^36^ proposed the bioconvection in a nanofluid having gyrotactic microorganisms. Kuznetsov^37^ developed the same idea by adding nanoparticles in order to the stability of nanofluid. Xun et al.^38^ analyzed the bioconvective fluid flow containing gyrotactic microorganisms between two rotating plates. Further studies based on nanofluids containing gyrotactic microorganisms are mentioned in^39–45^.

Based on the literature review, the theoretical analysis of two-dimensional electrically conducting and magnetically susceptible viscoelastic micropolar nanofluid containing nanoparticles and gyrotactic microorganisms through a stratified stretching sheet has not been performed yet. Thus, the authors have presented the viscoelastic micropolar nanofluid containing nanoparticles and gyrotactic microorganisms through a stratified stretching sheet. Furthermore bioconvection, binary chemical reaction, thermal radiation, and activation energy influences are taken into assumptions.

## Problem formulation

**Figure 1:**
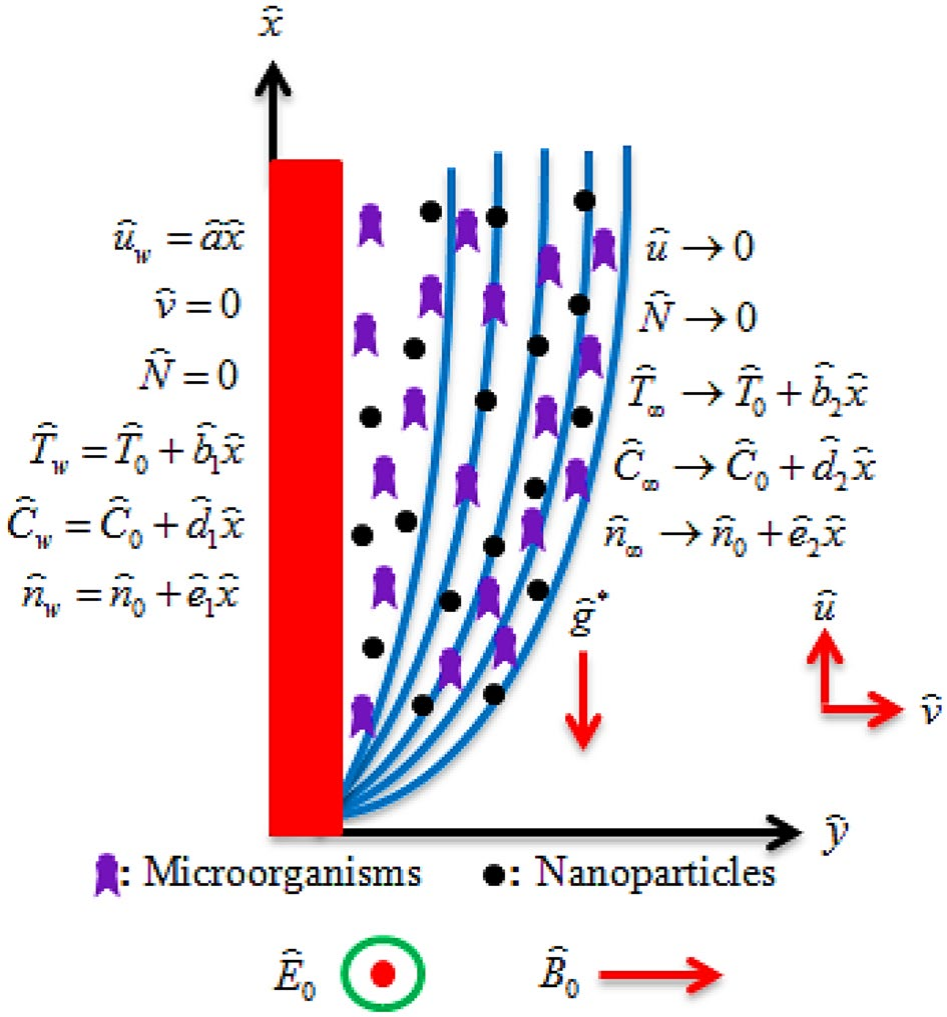
Physical illustration of the flow problem.

Let us assume the incompressible and electrically conducting MHD two-dimensional bioconvective viscoelastic micropolar nanofluid flow containing gyrotactic microorganisms which propagate over a stratified stretching sheet. Microorganisms are brought to become the nanoparticles stable. The nanoparticles do not affect the microorganisms’ velocity and swimming direction. The stretching velocity of the sheet is assumed as $$\overset{\lower0.5em\hbox{$\smash{\scriptscriptstyle\frown}$}}{u}_{w} = \overset{\lower0.5em\hbox{$\smash{\scriptscriptstyle\frown}$}}{a} \overset{\lower0.5em\hbox{$\smash{\scriptscriptstyle\frown}$}}{x}$$ along $$\overset{\lower0.5em\hbox{$\smash{\scriptscriptstyle\frown}$}}{x} -$$direction, whereas $$\overset{\lower0.5em\hbox{$\smash{\scriptscriptstyle\frown}$}}{y} -$$direction is normal to the nanofluid flow. Magnetic $$B = \left( {0,\overset{\lower0.5em\hbox{$\smash{\scriptscriptstyle\frown}$}}{B}_{0} ,0} \right)$$ and electric $$\overline{E} = \left( {0,0,\, - \overset{\lower0.5em\hbox{$\smash{\scriptscriptstyle\frown}$}}{E}_{0} } \right)$$ fields are applied normal to the nanofluid flow. Furthermore, binary chemical reaction, activation energy, thermal radiation, mixed convection, and Joule heating influences are taken into consideration. Figure 1 indicates the physical representation of the flow problem. Following the above assumptions, the leading equations are formulated as^33,46–48^:

### Continuity equation

1 $$ \frac{{\partial \overset{\lower0.5em\hbox{$\smash{\scriptscriptstyle\frown}$}}{u} }}{{\partial \overset{\lower0.5em\hbox{$\smash{\scriptscriptstyle\frown}$}}{x} }} + \frac{{\partial \overset{\lower0.5em\hbox{$\smash{\scriptscriptstyle\frown}$}}{v} }}{{\partial \overset{\lower0.5em\hbox{$\smash{\scriptscriptstyle\frown}$}}{y} }} = 0, $$

### Momentum equation

2 $$ \begin{aligned} \overset{\lower0.5em\hbox{$\smash{\scriptscriptstyle\frown}$}}{u} \frac{{\partial \overset{\lower0.5em\hbox{$\smash{\scriptscriptstyle\frown}$}}{u} }}{{\partial \overset{\lower0.5em\hbox{$\smash{\scriptscriptstyle\frown}$}}{x} }} + \overset{\lower0.5em\hbox{$\smash{\scriptscriptstyle\frown}$}}{v} \frac{{\partial \overset{\lower0.5em\hbox{$\smash{\scriptscriptstyle\frown}$}}{u} }}{{\partial \overset{\lower0.5em\hbox{$\smash{\scriptscriptstyle\frown}$}}{y} }} & = \left( {\overset{\lower0.5em\hbox{$\smash{\scriptscriptstyle\frown}$}}{\nu }_{f} + \frac{{\overset{\lower0.5em\hbox{$\smash{\scriptscriptstyle\frown}$}}{k}_{f} }}{{\overset{\lower0.5em\hbox{$\smash{\scriptscriptstyle\frown}$}}{\rho }_{f} }}} \right)\frac{{\partial^{2} \overset{\lower0.5em\hbox{$\smash{\scriptscriptstyle\frown}$}}{u} }}{{\partial \overset{\lower0.5em\hbox{$\smash{\scriptscriptstyle\frown}$}}{y}^{2} }} + \frac{{\overset{\lower0.5em\hbox{$\smash{\scriptscriptstyle\frown}$}}{k}_{f} }}{{\overset{\lower0.5em\hbox{$\smash{\scriptscriptstyle\frown}$}}{\rho }_{f} }}\frac{{\partial \overset{\lower0.5em\hbox{$\smash{\scriptscriptstyle\frown}$}}{N} }}{{\partial \overset{\lower0.5em\hbox{$\smash{\scriptscriptstyle\frown}$}}{y} }} - \frac{{\overset{\lower0.5em\hbox{$\smash{\scriptscriptstyle\frown}$}}{\alpha } }}{{\overset{\lower0.5em\hbox{$\smash{\scriptscriptstyle\frown}$}}{\rho }_{f} }}\left( {\frac{\partial }{{\partial \overset{\lower0.5em\hbox{$\smash{\scriptscriptstyle\frown}$}}{x} }}\left( {\overset{\lower0.5em\hbox{$\smash{\scriptscriptstyle\frown}$}}{u} \frac{{\partial^{2} \overset{\lower0.5em\hbox{$\smash{\scriptscriptstyle\frown}$}}{u} }}{{\partial \overset{\lower0.5em\hbox{$\smash{\scriptscriptstyle\frown}$}}{y}^{2} }}} \right) - \frac{{\partial \overset{\lower0.5em\hbox{$\smash{\scriptscriptstyle\frown}$}}{u} }}{{\partial \overset{\lower0.5em\hbox{$\smash{\scriptscriptstyle\frown}$}}{y} }}\frac{{\partial^{2} \overset{\lower0.5em\hbox{$\smash{\scriptscriptstyle\frown}$}}{u} }}{{\partial \overset{\lower0.5em\hbox{$\smash{\scriptscriptstyle\frown}$}}{x} \partial \overset{\lower0.5em\hbox{$\smash{\scriptscriptstyle\frown}$}}{y} }} + \overset{\lower0.5em\hbox{$\smash{\scriptscriptstyle\frown}$}}{v} \frac{{\partial^{3} \overset{\lower0.5em\hbox{$\smash{\scriptscriptstyle\frown}$}}{u} }}{{\partial \overset{\lower0.5em\hbox{$\smash{\scriptscriptstyle\frown}$}}{y}^{3} }}} \right) + \frac{{\overset{\lower0.5em\hbox{$\smash{\scriptscriptstyle\frown}$}}{\sigma } }}{{\overset{\lower0.5em\hbox{$\smash{\scriptscriptstyle\frown}$}}{\rho }_{f} }}\overset{\lower0.5em\hbox{$\smash{\scriptscriptstyle\frown}$}}{E}_{0} \overset{\lower0.5em\hbox{$\smash{\scriptscriptstyle\frown}$}}{B}_{0} \\ & \quad - \frac{{\overset{\lower0.5em\hbox{$\smash{\scriptscriptstyle\frown}$}}{\sigma } }}{{\overset{\lower0.5em\hbox{$\smash{\scriptscriptstyle\frown}$}}{\rho }_{f} }}\overset{\lower0.5em\hbox{$\smash{\scriptscriptstyle\frown}$}}{B}_{0}^{2} \overset{\lower0.5em\hbox{$\smash{\scriptscriptstyle\frown}$}}{u} + \frac{1}{{\overset{\lower0.5em\hbox{$\smash{\scriptscriptstyle\frown}$}}{\rho }_{f} }}\left[ {\overset{\lower0.5em\hbox{$\smash{\scriptscriptstyle\frown}$}}{\rho }_{f} \overset{\lower0.5em\hbox{$\smash{\scriptscriptstyle\frown}$}}{\beta }^{*} \overset{\lower0.5em\hbox{$\smash{\scriptscriptstyle\frown}$}}{g}^{*} \left( {\overset{\lower0.5em\hbox{$\smash{\scriptscriptstyle\frown}$}}{T} - \overset{\lower0.5em\hbox{$\smash{\scriptscriptstyle\frown}$}}{T}_{\infty } } \right) - \overset{\lower0.5em\hbox{$\smash{\scriptscriptstyle\frown}$}}{g}^{*} \left( {\overset{\lower0.5em\hbox{$\smash{\scriptscriptstyle\frown}$}}{\rho }_{p} - \overset{\lower0.5em\hbox{$\smash{\scriptscriptstyle\frown}$}}{\rho }_{f} } \right)\left( {\overset{\lower0.5em\hbox{$\smash{\scriptscriptstyle\frown}$}}{C} - \overset{\lower0.5em\hbox{$\smash{\scriptscriptstyle\frown}$}}{C}_{\infty } } \right) - \overset{\lower0.5em\hbox{$\smash{\scriptscriptstyle\frown}$}}{\gamma }^{*} \overset{\lower0.5em\hbox{$\smash{\scriptscriptstyle\frown}$}}{g}^{*} \left( {\overset{\lower0.5em\hbox{$\smash{\scriptscriptstyle\frown}$}}{\rho }_{m} - \overset{\lower0.5em\hbox{$\smash{\scriptscriptstyle\frown}$}}{\rho }_{f} } \right)\left( {\overset{\lower0.5em\hbox{$\smash{\scriptscriptstyle\frown}$}}{n} - \overset{\lower0.5em\hbox{$\smash{\scriptscriptstyle\frown}$}}{n}_{\infty } } \right)} \right], \\ \end{aligned} $$

with boundary conditions:

3 $$ \left\{ \begin{gathered} \overset{\lower0.5em\hbox{$\smash{\scriptscriptstyle\frown}$}}{u} = \overset{\lower0.5em\hbox{$\smash{\scriptscriptstyle\frown}$}}{u}_{w} = \overset{\lower0.5em\hbox{$\smash{\scriptscriptstyle\frown}$}}{a} \overset{\lower0.5em\hbox{$\smash{\scriptscriptstyle\frown}$}}{x} ,\,\,\overset{\lower0.5em\hbox{$\smash{\scriptscriptstyle\frown}$}}{v} = 0\,\,at\,\,\overset{\lower0.5em\hbox{$\smash{\scriptscriptstyle\frown}$}}{y} = 0, \hfill \\ \,\,\overset{\lower0.5em\hbox{$\smash{\scriptscriptstyle\frown}$}}{u} \to 0\,\,as\,\,\overset{\lower0.5em\hbox{$\smash{\scriptscriptstyle\frown}$}}{y} \to \infty , \hfill \\ \end{gathered} \right\} $$

The correspondence transformations are defined as^5,49–51^:

4 $$ \left\{ \begin{gathered} \overset{\lower0.5em\hbox{$\smash{\scriptscriptstyle\frown}$}}{u} = \overset{\lower0.5em\hbox{$\smash{\scriptscriptstyle\frown}$}}{a} \overset{\lower0.5em\hbox{$\smash{\scriptscriptstyle\frown}$}}{x} f^{\prime}\left( \xi \right),\,\,\overset{\lower0.5em\hbox{$\smash{\scriptscriptstyle\frown}$}}{v} = - \sqrt {\overset{\lower0.5em\hbox{$\smash{\scriptscriptstyle\frown}$}}{a} \overset{\lower0.5em\hbox{$\smash{\scriptscriptstyle\frown}$}}{\nu }_{f} } f\left( \xi \right),\,\,\overset{\lower0.5em\hbox{$\smash{\scriptscriptstyle\frown}$}}{N} = \sqrt {\frac{{\overset{\lower0.5em\hbox{$\smash{\scriptscriptstyle\frown}$}}{a} }}{{\overset{\lower0.5em\hbox{$\smash{\scriptscriptstyle\frown}$}}{\nu }_{f} }}} \overset{\lower0.5em\hbox{$\smash{\scriptscriptstyle\frown}$}}{a} \overset{\lower0.5em\hbox{$\smash{\scriptscriptstyle\frown}$}}{x} g\left( \xi \right),\,\, \hfill \\ \theta \left( \xi \right) = \frac{{\overset{\lower0.5em\hbox{$\smash{\scriptscriptstyle\frown}$}}{T} - \overset{\lower0.5em\hbox{$\smash{\scriptscriptstyle\frown}$}}{T}_{\infty } }}{{\overset{\lower0.5em\hbox{$\smash{\scriptscriptstyle\frown}$}}{T}_{w} - \overset{\lower0.5em\hbox{$\smash{\scriptscriptstyle\frown}$}}{T}_{0} }},\,\,\phi \left( \xi \right) = \frac{{\overset{\lower0.5em\hbox{$\smash{\scriptscriptstyle\frown}$}}{C} - \overset{\lower0.5em\hbox{$\smash{\scriptscriptstyle\frown}$}}{C}_{\infty } }}{{\overset{\lower0.5em\hbox{$\smash{\scriptscriptstyle\frown}$}}{C}_{w} - \overset{\lower0.5em\hbox{$\smash{\scriptscriptstyle\frown}$}}{C}_{0} }},\,\,\chi \left( \xi \right) = \frac{{\overset{\lower0.5em\hbox{$\smash{\scriptscriptstyle\frown}$}}{n} - \overset{\lower0.5em\hbox{$\smash{\scriptscriptstyle\frown}$}}{n}_{\infty } }}{{\overset{\lower0.5em\hbox{$\smash{\scriptscriptstyle\frown}$}}{n}_{w} - \overset{\lower0.5em\hbox{$\smash{\scriptscriptstyle\frown}$}}{n}_{0} }},\,\,\xi = \sqrt {\frac{{\overset{\lower0.5em\hbox{$\smash{\scriptscriptstyle\frown}$}}{a} }}{{\overset{\lower0.5em\hbox{$\smash{\scriptscriptstyle\frown}$}}{\nu }_{f} }}} \overset{\lower0.5em\hbox{$\smash{\scriptscriptstyle\frown}$}}{y} . \hfill \\ \end{gathered} \right\} $$

Using (4), (1) is identically contented, (2) and (3) are transformed as:

5 $$ \left( {1 + K} \right)f^{\prime\prime\prime} - f^{{\prime}{2}} + ff^{\prime\prime} + Kg^{\prime} - Mf^{\prime} - \alpha_{1} \left( {2f^{\prime}f^{\prime\prime\prime} - f^{{\prime\prime}{2}} - ff^{iv} } \right) + M\overline{E} + \lambda \left( {\theta - Nr\phi - Rb\chi } \right) = 0, $$

6 $$ f\left( 0 \right) = 0,\,\,f^{\prime}\left( 0 \right) = 1,\,\,f^{\prime}\left( \infty \right) = 0, $$

where $$K = {{\overset{\lower0.5em\hbox{$\smash{\scriptscriptstyle\frown}$}}{k}_{f} } \mathord{\left/ {\vphantom {{\overset{\lower0.5em\hbox{$\smash{\scriptscriptstyle\frown}$}}{k}_{f} } {\overset{\lower0.5em\hbox{$\smash{\scriptscriptstyle\frown}$}}{\mu }_{f} }}} \right. \kern-\nulldelimiterspace} {\overset{\lower0.5em\hbox{$\smash{\scriptscriptstyle\frown}$}}{\mu }_{f} }}$$ is the micropolar constant, $$\alpha_{1} = {{\overset{\lower0.5em\hbox{$\smash{\scriptscriptstyle\frown}$}}{\alpha } \overset{\lower0.5em\hbox{$\smash{\scriptscriptstyle\frown}$}}{a} } \mathord{\left/ {\vphantom {{\overset{\lower0.5em\hbox{$\smash{\scriptscriptstyle\frown}$}}{\alpha } \overset{\lower0.5em\hbox{$\smash{\scriptscriptstyle\frown}$}}{a} } {\overset{\lower0.5em\hbox{$\smash{\scriptscriptstyle\frown}$}}{\mu }_{f} }}} \right. \kern-\nulldelimiterspace} {\overset{\lower0.5em\hbox{$\smash{\scriptscriptstyle\frown}$}}{\mu }_{f} }}$$ is the viscoelastic parameter, $$M = {{\overset{\lower0.5em\hbox{$\smash{\scriptscriptstyle\frown}$}}{\sigma } \overset{\lower0.5em\hbox{$\smash{\scriptscriptstyle\frown}$}}{B}_{0}^{2} } \mathord{\left/ {\vphantom {{\overset{\lower0.5em\hbox{$\smash{\scriptscriptstyle\frown}$}}{\sigma } \overset{\lower0.5em\hbox{$\smash{\scriptscriptstyle\frown}$}}{B}_{0}^{2} } {\overset{\lower0.5em\hbox{$\smash{\scriptscriptstyle\frown}$}}{\rho }_{f} \overset{\lower0.5em\hbox{$\smash{\scriptscriptstyle\frown}$}}{a} }}} \right. \kern-\nulldelimiterspace} {\overset{\lower0.5em\hbox{$\smash{\scriptscriptstyle\frown}$}}{\rho }_{f} \overset{\lower0.5em\hbox{$\smash{\scriptscriptstyle\frown}$}}{a} }}$$ is the magnetic parameter, $$\overline{E} = {{\overset{\lower0.5em\hbox{$\smash{\scriptscriptstyle\frown}$}}{E}_{0} } \mathord{\left/ {\vphantom {{\overset{\lower0.5em\hbox{$\smash{\scriptscriptstyle\frown}$}}{E}_{0} } {\overset{\lower0.5em\hbox{$\smash{\scriptscriptstyle\frown}$}}{u}_{w} \overset{\lower0.5em\hbox{$\smash{\scriptscriptstyle\frown}$}}{B}_{0} }}} \right. \kern-\nulldelimiterspace} {\overset{\lower0.5em\hbox{$\smash{\scriptscriptstyle\frown}$}}{u}_{w} \overset{\lower0.5em\hbox{$\smash{\scriptscriptstyle\frown}$}}{B}_{0} }}$$ is the electric parameter, $$\lambda = {{\overset{\lower0.5em\hbox{$\smash{\scriptscriptstyle\frown}$}}{\beta }^{*} \overset{\lower0.5em\hbox{$\smash{\scriptscriptstyle\frown}$}}{g}^{*} \left( {\overset{\lower0.5em\hbox{$\smash{\scriptscriptstyle\frown}$}}{T}_{w} - \overset{\lower0.5em\hbox{$\smash{\scriptscriptstyle\frown}$}}{T}_{0} } \right)} \mathord{\left/ {\vphantom {{\overset{\lower0.5em\hbox{$\smash{\scriptscriptstyle\frown}$}}{\beta }^{*} \overset{\lower0.5em\hbox{$\smash{\scriptscriptstyle\frown}$}}{g}^{*} \left( {\overset{\lower0.5em\hbox{$\smash{\scriptscriptstyle\frown}$}}{T}_{w} - \overset{\lower0.5em\hbox{$\smash{\scriptscriptstyle\frown}$}}{T}_{0} } \right)} {\overset{\lower0.5em\hbox{$\smash{\scriptscriptstyle\frown}$}}{x} \overset{\lower0.5em\hbox{$\smash{\scriptscriptstyle\frown}$}}{a}^{2} }}} \right. \kern-\nulldelimiterspace} {\overset{\lower0.5em\hbox{$\smash{\scriptscriptstyle\frown}$}}{x} \overset{\lower0.5em\hbox{$\smash{\scriptscriptstyle\frown}$}}{a}^{2} }}$$ is the mixed convection parameter, $$Nr = {{\left( {\overset{\lower0.5em\hbox{$\smash{\scriptscriptstyle\frown}$}}{\rho }_{p} - \overset{\lower0.5em\hbox{$\smash{\scriptscriptstyle\frown}$}}{\rho }_{f} } \right)\left( {\overset{\lower0.5em\hbox{$\smash{\scriptscriptstyle\frown}$}}{C}_{w} - \overset{\lower0.5em\hbox{$\smash{\scriptscriptstyle\frown}$}}{C}_{0} } \right)} \mathord{\left/ {\vphantom {{\left( {\overset{\lower0.5em\hbox{$\smash{\scriptscriptstyle\frown}$}}{\rho }_{p} - \overset{\lower0.5em\hbox{$\smash{\scriptscriptstyle\frown}$}}{\rho }_{f} } \right)\left( {\overset{\lower0.5em\hbox{$\smash{\scriptscriptstyle\frown}$}}{C}_{w} - \overset{\lower0.5em\hbox{$\smash{\scriptscriptstyle\frown}$}}{C}_{0} } \right)} {\overset{\lower0.5em\hbox{$\smash{\scriptscriptstyle\frown}$}}{\rho }_{f} \overset{\lower0.5em\hbox{$\smash{\scriptscriptstyle\frown}$}}{\beta }^{*} \left( {\overset{\lower0.5em\hbox{$\smash{\scriptscriptstyle\frown}$}}{T}_{w} - \overset{\lower0.5em\hbox{$\smash{\scriptscriptstyle\frown}$}}{T}_{0} } \right)}}} \right. \kern-\nulldelimiterspace} {\overset{\lower0.5em\hbox{$\smash{\scriptscriptstyle\frown}$}}{\rho }_{f} \overset{\lower0.5em\hbox{$\smash{\scriptscriptstyle\frown}$}}{\beta }^{*} \left( {\overset{\lower0.5em\hbox{$\smash{\scriptscriptstyle\frown}$}}{T}_{w} - \overset{\lower0.5em\hbox{$\smash{\scriptscriptstyle\frown}$}}{T}_{0} } \right)}}$$ is the buoyancy ratio parameter, and $$Rb = \overset{\lower0.5em\hbox{$\smash{\scriptscriptstyle\frown}$}}{\gamma }^{*} {{\left( {\overset{\lower0.5em\hbox{$\smash{\scriptscriptstyle\frown}$}}{\rho }_{m} - \overset{\lower0.5em\hbox{$\smash{\scriptscriptstyle\frown}$}}{\rho }_{f} } \right)\left( {\overset{\lower0.5em\hbox{$\smash{\scriptscriptstyle\frown}$}}{n}_{w} - \overset{\lower0.5em\hbox{$\smash{\scriptscriptstyle\frown}$}}{n}_{0} } \right)} \mathord{\left/ {\vphantom {{\left( {\overset{\lower0.5em\hbox{$\smash{\scriptscriptstyle\frown}$}}{\rho }_{m} - \overset{\lower0.5em\hbox{$\smash{\scriptscriptstyle\frown}$}}{\rho }_{f} } \right)\left( {\overset{\lower0.5em\hbox{$\smash{\scriptscriptstyle\frown}$}}{n}_{w} - \overset{\lower0.5em\hbox{$\smash{\scriptscriptstyle\frown}$}}{n}_{0} } \right)} {\overset{\lower0.5em\hbox{$\smash{\scriptscriptstyle\frown}$}}{\rho }_{f} \overset{\lower0.5em\hbox{$\smash{\scriptscriptstyle\frown}$}}{\beta }^{*} \left( {\overset{\lower0.5em\hbox{$\smash{\scriptscriptstyle\frown}$}}{T}_{w} - \overset{\lower0.5em\hbox{$\smash{\scriptscriptstyle\frown}$}}{T}_{0} } \right)}}} \right. \kern-\nulldelimiterspace} {\overset{\lower0.5em\hbox{$\smash{\scriptscriptstyle\frown}$}}{\rho }_{f} \overset{\lower0.5em\hbox{$\smash{\scriptscriptstyle\frown}$}}{\beta }^{*} \left( {\overset{\lower0.5em\hbox{$\smash{\scriptscriptstyle\frown}$}}{T}_{w} - \overset{\lower0.5em\hbox{$\smash{\scriptscriptstyle\frown}$}}{T}_{0} } \right)}}$$ is the bioconvection Rayleigh number.

### Angular momentum equation

7 $$ \overset{\lower0.5em\hbox{$\smash{\scriptscriptstyle\frown}$}}{u} \frac{{\partial \overset{\lower0.5em\hbox{$\smash{\scriptscriptstyle\frown}$}}{N} }}{{\partial \overset{\lower0.5em\hbox{$\smash{\scriptscriptstyle\frown}$}}{x} }} + \overset{\lower0.5em\hbox{$\smash{\scriptscriptstyle\frown}$}}{v} \frac{{\partial \overset{\lower0.5em\hbox{$\smash{\scriptscriptstyle\frown}$}}{N} }}{{\partial \overset{\lower0.5em\hbox{$\smash{\scriptscriptstyle\frown}$}}{y} }} = \frac{{\overset{\lower0.5em\hbox{$\smash{\scriptscriptstyle\frown}$}}{\gamma }_{f} }}{{\left( {\overset{\lower0.5em\hbox{$\smash{\scriptscriptstyle\frown}$}}{\rho } j} \right)_{f} }}\frac{{\partial^{2} \overset{\lower0.5em\hbox{$\smash{\scriptscriptstyle\frown}$}}{N} }}{{\partial \overset{\lower0.5em\hbox{$\smash{\scriptscriptstyle\frown}$}}{y}^{2} }} - \frac{{\overset{\lower0.5em\hbox{$\smash{\scriptscriptstyle\frown}$}}{k}_{f} }}{{\left( {\overset{\lower0.5em\hbox{$\smash{\scriptscriptstyle\frown}$}}{\rho } j} \right)_{f} }}\left( {2\overset{\lower0.5em\hbox{$\smash{\scriptscriptstyle\frown}$}}{N} + \frac{{\partial \overset{\lower0.5em\hbox{$\smash{\scriptscriptstyle\frown}$}}{u} }}{{\partial \overset{\lower0.5em\hbox{$\smash{\scriptscriptstyle\frown}$}}{y} }}} \right), $$

with boundary conditions:

8 $$ \begin{gathered} \overset{\lower0.5em\hbox{$\smash{\scriptscriptstyle\frown}$}}{N} = 0\,\,at\,\,\overset{\lower0.5em\hbox{$\smash{\scriptscriptstyle\frown}$}}{y} = 0,\,\, \hfill \\ \overset{\lower0.5em\hbox{$\smash{\scriptscriptstyle\frown}$}}{N} \to 0\,\,as\,\,\overset{\lower0.5em\hbox{$\smash{\scriptscriptstyle\frown}$}}{y} \to \infty , \hfill \\ \end{gathered} $$

Using (4), (7) and (8) are transformed as:

9 $$ \left( {1 + \frac{K}{2}} \right)g^{\prime\prime} - gf^{\prime} + fg^{\prime} - K\left( {2g + f^{\prime\prime}} \right) = 0, $$

10 $$ g\left( 0 \right) = 0,\,\,g\left( \infty \right) = 0. $$

### Temperature equation

11 $$ \overset{\lower0.5em\hbox{$\smash{\scriptscriptstyle\frown}$}}{u} \frac{{\partial \overset{\lower0.5em\hbox{$\smash{\scriptscriptstyle\frown}$}}{T} }}{{\partial \overset{\lower0.5em\hbox{$\smash{\scriptscriptstyle\frown}$}}{x} }} + \overset{\lower0.5em\hbox{$\smash{\scriptscriptstyle\frown}$}}{v} \frac{{\partial \overset{\lower0.5em\hbox{$\smash{\scriptscriptstyle\frown}$}}{T} }}{{\partial \overset{\lower0.5em\hbox{$\smash{\scriptscriptstyle\frown}$}}{y} }} = \overset{\lower0.5em\hbox{$\smash{\scriptscriptstyle\frown}$}}{\alpha }_{f} \frac{{\partial^{2} \overset{\lower0.5em\hbox{$\smash{\scriptscriptstyle\frown}$}}{T} }}{{\partial \overset{\lower0.5em\hbox{$\smash{\scriptscriptstyle\frown}$}}{y}^{2} }} + \frac{{Q_{0} }}{{\left( {\overset{\lower0.5em\hbox{$\smash{\scriptscriptstyle\frown}$}}{\rho } \overset{\lower0.5em\hbox{$\smash{\scriptscriptstyle\frown}$}}{c}_{p} } \right)_{f} }}\left( {\overset{\lower0.5em\hbox{$\smash{\scriptscriptstyle\frown}$}}{T} - \overset{\lower0.5em\hbox{$\smash{\scriptscriptstyle\frown}$}}{T}_{\infty } } \right) - \frac{1}{{\left( {\overset{\lower0.5em\hbox{$\smash{\scriptscriptstyle\frown}$}}{\rho } \overset{\lower0.5em\hbox{$\smash{\scriptscriptstyle\frown}$}}{c}_{p} } \right)_{f} }}\frac{{\partial \overset{\lower0.5em\hbox{$\smash{\scriptscriptstyle\frown}$}}{q}_{r} }}{{\partial \overset{\lower0.5em\hbox{$\smash{\scriptscriptstyle\frown}$}}{y} }} + \frac{{\overset{\lower0.5em\hbox{$\smash{\scriptscriptstyle\frown}$}}{\sigma } }}{{\left( {\overset{\lower0.5em\hbox{$\smash{\scriptscriptstyle\frown}$}}{\rho } \overset{\lower0.5em\hbox{$\smash{\scriptscriptstyle\frown}$}}{c}_{p} } \right)_{f} }}\left( {\overset{\lower0.5em\hbox{$\smash{\scriptscriptstyle\frown}$}}{B}_{0} \overset{\lower0.5em\hbox{$\smash{\scriptscriptstyle\frown}$}}{u} - \overset{\lower0.5em\hbox{$\smash{\scriptscriptstyle\frown}$}}{E}_{0} } \right)^{2} , $$

with boundary conditions:

12 $$ \begin{gathered} \overset{\lower0.5em\hbox{$\smash{\scriptscriptstyle\frown}$}}{T} = \overset{\lower0.5em\hbox{$\smash{\scriptscriptstyle\frown}$}}{T}_{w} = \overset{\lower0.5em\hbox{$\smash{\scriptscriptstyle\frown}$}}{T}_{0} + \overset{\lower0.5em\hbox{$\smash{\scriptscriptstyle\frown}$}}{b}_{1} \overset{\lower0.5em\hbox{$\smash{\scriptscriptstyle\frown}$}}{x} \,\,at\,\,\overset{\lower0.5em\hbox{$\smash{\scriptscriptstyle\frown}$}}{y} = 0, \hfill \\ \overset{\lower0.5em\hbox{$\smash{\scriptscriptstyle\frown}$}}{T} = \overset{\lower0.5em\hbox{$\smash{\scriptscriptstyle\frown}$}}{T}_{\infty } = \overset{\lower0.5em\hbox{$\smash{\scriptscriptstyle\frown}$}}{T}_{0} + \overset{\lower0.5em\hbox{$\smash{\scriptscriptstyle\frown}$}}{b}_{2} \overset{\lower0.5em\hbox{$\smash{\scriptscriptstyle\frown}$}}{x} \,\,as\,\,\overset{\lower0.5em\hbox{$\smash{\scriptscriptstyle\frown}$}}{y} \to \infty , \hfill \\ \end{gathered} $$

Here, $$\overset{\lower0.5em\hbox{$\smash{\scriptscriptstyle\frown}$}}{q}_{r}$$ is the radiative heat flux and is defined as:

13 $$ \overset{\lower0.5em\hbox{$\smash{\scriptscriptstyle\frown}$}}{q}_{r} = - \frac{{4\overset{\lower0.5em\hbox{$\smash{\scriptscriptstyle\frown}$}}{\sigma }^{*} }}{{3\overset{\lower0.5em\hbox{$\smash{\scriptscriptstyle\frown}$}}{k}^{*} }}\frac{{\partial \overset{\lower0.5em\hbox{$\smash{\scriptscriptstyle\frown}$}}{T}^{4} }}{{\partial \overset{\lower0.5em\hbox{$\smash{\scriptscriptstyle\frown}$}}{y} }}. $$

Expending $$\overset{\lower0.5em\hbox{$\smash{\scriptscriptstyle\frown}$}}{T}^{4}$$ by mean of Taylor series, we have:

14 $$ \overset{\lower0.5em\hbox{$\smash{\scriptscriptstyle\frown}$}}{T}^{4} = \overset{\lower0.5em\hbox{$\smash{\scriptscriptstyle\frown}$}}{T}_{\infty }^{4} + 4\overset{\lower0.5em\hbox{$\smash{\scriptscriptstyle\frown}$}}{T}_{\infty }^{3} \left( {\overset{\lower0.5em\hbox{$\smash{\scriptscriptstyle\frown}$}}{T} - \overset{\lower0.5em\hbox{$\smash{\scriptscriptstyle\frown}$}}{T}_{\infty } } \right) + 6\overset{\lower0.5em\hbox{$\smash{\scriptscriptstyle\frown}$}}{T}_{\infty }^{2} \left( {\overset{\lower0.5em\hbox{$\smash{\scriptscriptstyle\frown}$}}{T} - \overset{\lower0.5em\hbox{$\smash{\scriptscriptstyle\frown}$}}{T}_{\infty } } \right)^{2} + \cdots $$

Using (4), (11) and (12) are transformed as:

15 $$ \left( {1 + \frac{4}{3}Rd} \right)\theta^{\prime\prime} + \Pr f\theta^{\prime} - \Pr f^{\prime}\theta - \Pr Sf^{\prime} + \Pr \delta \theta + M^{2} Ec\left[ {f^{{\prime}{2}} + \overline{E}^{2} - 2\overline{E}f^{\prime}} \right] = 0, $$

16 $$ \theta \left( 0 \right) = 1 - S,\,\,\theta \left( \infty \right) = 0, $$

where $$Rd = {{4\overset{\lower0.5em\hbox{$\smash{\scriptscriptstyle\frown}$}}{\sigma }^{*} \overset{\lower0.5em\hbox{$\smash{\scriptscriptstyle\frown}$}}{T}_{\infty }^{3} } \mathord{\left/ {\vphantom {{4\overset{\lower0.5em\hbox{$\smash{\scriptscriptstyle\frown}$}}{\sigma }^{*} \overset{\lower0.5em\hbox{$\smash{\scriptscriptstyle\frown}$}}{T}_{\infty }^{3} } {\overset{\lower0.5em\hbox{$\smash{\scriptscriptstyle\frown}$}}{k} \overset{\lower0.5em\hbox{$\smash{\scriptscriptstyle\frown}$}}{k}^{*} }}} \right. \kern-\nulldelimiterspace} {\overset{\lower0.5em\hbox{$\smash{\scriptscriptstyle\frown}$}}{k} \overset{\lower0.5em\hbox{$\smash{\scriptscriptstyle\frown}$}}{k}^{*} }}$$ is the thermal radiation parameter, $$\Pr = {{\overset{\lower0.5em\hbox{$\smash{\scriptscriptstyle\frown}$}}{\nu }_{f} } \mathord{\left/ {\vphantom {{\overset{\lower0.5em\hbox{$\smash{\scriptscriptstyle\frown}$}}{\nu }_{f} } {\overset{\lower0.5em\hbox{$\smash{\scriptscriptstyle\frown}$}}{\alpha }_{f} }}} \right. \kern-\nulldelimiterspace} {\overset{\lower0.5em\hbox{$\smash{\scriptscriptstyle\frown}$}}{\alpha }_{f} }}$$ is the Prandtl number, $$S = {{\overset{\lower0.5em\hbox{$\smash{\scriptscriptstyle\frown}$}}{b}_{2} } \mathord{\left/ {\vphantom {{\overset{\lower0.5em\hbox{$\smash{\scriptscriptstyle\frown}$}}{b}_{2} } {\overset{\lower0.5em\hbox{$\smash{\scriptscriptstyle\frown}$}}{b}_{1} }}} \right. \kern-\nulldelimiterspace} {\overset{\lower0.5em\hbox{$\smash{\scriptscriptstyle\frown}$}}{b}_{1} }}$$ is the thermal stratification parameter, $$Ec = {{\left( {\overset{\lower0.5em\hbox{$\smash{\scriptscriptstyle\frown}$}}{a} \overset{\lower0.5em\hbox{$\smash{\scriptscriptstyle\frown}$}}{x} } \right)^{2} } \mathord{\left/ {\vphantom {{\left( {\overset{\lower0.5em\hbox{$\smash{\scriptscriptstyle\frown}$}}{a} \overset{\lower0.5em\hbox{$\smash{\scriptscriptstyle\frown}$}}{x} } \right)^{2} } {\left( {\overset{\lower0.5em\hbox{$\smash{\scriptscriptstyle\frown}$}}{c}_{p} } \right)_{f} \left( {\overset{\lower0.5em\hbox{$\smash{\scriptscriptstyle\frown}$}}{T}_{w} - \overset{\lower0.5em\hbox{$\smash{\scriptscriptstyle\frown}$}}{T}_{\infty } } \right)}}} \right. \kern-\nulldelimiterspace} {\left( {\overset{\lower0.5em\hbox{$\smash{\scriptscriptstyle\frown}$}}{c}_{p} } \right)_{f} \left( {\overset{\lower0.5em\hbox{$\smash{\scriptscriptstyle\frown}$}}{T}_{w} - \overset{\lower0.5em\hbox{$\smash{\scriptscriptstyle\frown}$}}{T}_{\infty } } \right)}}$$ is the Eckert number, $$\delta = {{Q_{0} } \mathord{\left/ {\vphantom {{Q_{0} } {\overset{\lower0.5em\hbox{$\smash{\scriptscriptstyle\frown}$}}{a} \left( {\overset{\lower0.5em\hbox{$\smash{\scriptscriptstyle\frown}$}}{\rho } \overset{\lower0.5em\hbox{$\smash{\scriptscriptstyle\frown}$}}{c}_{p} } \right)_{f} }}} \right. \kern-\nulldelimiterspace} {\overset{\lower0.5em\hbox{$\smash{\scriptscriptstyle\frown}$}}{a} \left( {\overset{\lower0.5em\hbox{$\smash{\scriptscriptstyle\frown}$}}{\rho } \overset{\lower0.5em\hbox{$\smash{\scriptscriptstyle\frown}$}}{c}_{p} } \right)_{f} }}$$ is the heat generation parameter.

### Concentration equation

17 $$ \overset{\lower0.5em\hbox{$\smash{\scriptscriptstyle\frown}$}}{u} \frac{{\partial \overset{\lower0.5em\hbox{$\smash{\scriptscriptstyle\frown}$}}{C} }}{{\partial \overset{\lower0.5em\hbox{$\smash{\scriptscriptstyle\frown}$}}{x} }} + \overset{\lower0.5em\hbox{$\smash{\scriptscriptstyle\frown}$}}{v} \frac{{\partial \overset{\lower0.5em\hbox{$\smash{\scriptscriptstyle\frown}$}}{C} }}{{\partial \overset{\lower0.5em\hbox{$\smash{\scriptscriptstyle\frown}$}}{y} }} = \overset{\lower0.5em\hbox{$\smash{\scriptscriptstyle\frown}$}}{D}_{{\overset{\lower0.5em\hbox{$\smash{\scriptscriptstyle\frown}$}}{B} }} \frac{{\partial^{2} \overset{\lower0.5em\hbox{$\smash{\scriptscriptstyle\frown}$}}{C} }}{{\partial \overset{\lower0.5em\hbox{$\smash{\scriptscriptstyle\frown}$}}{y}^{2} }} - \overset{\lower0.5em\hbox{$\smash{\scriptscriptstyle\frown}$}}{k} r^{2} \left( {\overset{\lower0.5em\hbox{$\smash{\scriptscriptstyle\frown}$}}{C} - \overset{\lower0.5em\hbox{$\smash{\scriptscriptstyle\frown}$}}{C}_{\infty } } \right)\left( {\frac{{\overset{\lower0.5em\hbox{$\smash{\scriptscriptstyle\frown}$}}{T} }}{{\overset{\lower0.5em\hbox{$\smash{\scriptscriptstyle\frown}$}}{T}_{\infty } }}} \right)^{{\overline{n}}} \exp \left[ { - \frac{{\overset{\lower0.5em\hbox{$\smash{\scriptscriptstyle\frown}$}}{E} a}}{{\overset{\lower0.5em\hbox{$\smash{\scriptscriptstyle\frown}$}}{\kappa } \overset{\lower0.5em\hbox{$\smash{\scriptscriptstyle\frown}$}}{T} }}} \right], $$

with boundary conditions:

18 $$ \begin{gathered} \overset{\lower0.5em\hbox{$\smash{\scriptscriptstyle\frown}$}}{C} = \overset{\lower0.5em\hbox{$\smash{\scriptscriptstyle\frown}$}}{C}_{w} = \overset{\lower0.5em\hbox{$\smash{\scriptscriptstyle\frown}$}}{C}_{0} + \overset{\lower0.5em\hbox{$\smash{\scriptscriptstyle\frown}$}}{d}_{1} \overset{\lower0.5em\hbox{$\smash{\scriptscriptstyle\frown}$}}{x} \,\,at\,\,\overset{\lower0.5em\hbox{$\smash{\scriptscriptstyle\frown}$}}{y} = 0, \hfill \\ \overset{\lower0.5em\hbox{$\smash{\scriptscriptstyle\frown}$}}{C} = \overset{\lower0.5em\hbox{$\smash{\scriptscriptstyle\frown}$}}{C}_{\infty } = \overset{\lower0.5em\hbox{$\smash{\scriptscriptstyle\frown}$}}{C}_{0} + \overset{\lower0.5em\hbox{$\smash{\scriptscriptstyle\frown}$}}{d}_{2} \overset{\lower0.5em\hbox{$\smash{\scriptscriptstyle\frown}$}}{x} \,\,as\,\,\overset{\lower0.5em\hbox{$\smash{\scriptscriptstyle\frown}$}}{y} \to \infty , \hfill \\ \end{gathered} $$

Using (4), (17) and (18) are transformed as:

19 $$ \phi^{\prime\prime} + Sc\left( {f\phi^{\prime} - f^{\prime}\phi - Qf^{\prime}} \right) - Sc\varpi \left( {1 + \varepsilon \theta } \right)^{{\overline{n}}} \phi \exp \left( {\frac{ - E}{{\left( {1 + \varepsilon \theta } \right)}}} \right) = 0, $$

20 $$ \phi \left( 0 \right) = 1 - Q,\,\,\phi \left( \infty \right) = 0, $$

where $$Sc{{ = \overset{\lower0.5em\hbox{$\smash{\scriptscriptstyle\frown}$}}{\nu }_{f} } \mathord{\left/ {\vphantom {{ = \overset{\lower0.5em\hbox{$\smash{\scriptscriptstyle\frown}$}}{\nu }_{f} } {\overset{\lower0.5em\hbox{$\smash{\scriptscriptstyle\frown}$}}{D}_{{\overset{\lower0.5em\hbox{$\smash{\scriptscriptstyle\frown}$}}{B} }} }}} \right. \kern-\nulldelimiterspace} {\overset{\lower0.5em\hbox{$\smash{\scriptscriptstyle\frown}$}}{D}_{{\overset{\lower0.5em\hbox{$\smash{\scriptscriptstyle\frown}$}}{B} }} }}$$ is the Schmidt number, $$\varpi = {{\overset{\lower0.5em\hbox{$\smash{\scriptscriptstyle\frown}$}}{k} r^{2} } \mathord{\left/ {\vphantom {{\overset{\lower0.5em\hbox{$\smash{\scriptscriptstyle\frown}$}}{k} r^{2} } {\overset{\lower0.5em\hbox{$\smash{\scriptscriptstyle\frown}$}}{a} }}} \right. \kern-\nulldelimiterspace} {\overset{\lower0.5em\hbox{$\smash{\scriptscriptstyle\frown}$}}{a} }}$$ is the reaction rate parameter, $$E = {{\overset{\lower0.5em\hbox{$\smash{\scriptscriptstyle\frown}$}}{E} a} \mathord{\left/ {\vphantom {{\overset{\lower0.5em\hbox{$\smash{\scriptscriptstyle\frown}$}}{E} a} {\overset{\lower0.5em\hbox{$\smash{\scriptscriptstyle\frown}$}}{\kappa } \overset{\lower0.5em\hbox{$\smash{\scriptscriptstyle\frown}$}}{T} }}} \right. \kern-\nulldelimiterspace} {\overset{\lower0.5em\hbox{$\smash{\scriptscriptstyle\frown}$}}{\kappa } \overset{\lower0.5em\hbox{$\smash{\scriptscriptstyle\frown}$}}{T} }}$$ is the activation energy parameter, $$Q = {{\overset{\lower0.5em\hbox{$\smash{\scriptscriptstyle\frown}$}}{d}_{2} } \mathord{\left/ {\vphantom {{\overset{\lower0.5em\hbox{$\smash{\scriptscriptstyle\frown}$}}{d}_{2} } {\overset{\lower0.5em\hbox{$\smash{\scriptscriptstyle\frown}$}}{d}_{1} }}} \right. \kern-\nulldelimiterspace} {\overset{\lower0.5em\hbox{$\smash{\scriptscriptstyle\frown}$}}{d}_{1} }}$$ is the mass stratification parameter, and $$\varepsilon = {{\left( {\overset{\lower0.5em\hbox{$\smash{\scriptscriptstyle\frown}$}}{T}_{w} - \overset{\lower0.5em\hbox{$\smash{\scriptscriptstyle\frown}$}}{T}_{0} } \right)} \mathord{\left/ {\vphantom {{\left( {\overset{\lower0.5em\hbox{$\smash{\scriptscriptstyle\frown}$}}{T}_{w} - \overset{\lower0.5em\hbox{$\smash{\scriptscriptstyle\frown}$}}{T}_{0} } \right)} {\overset{\lower0.5em\hbox{$\smash{\scriptscriptstyle\frown}$}}{T}_{\infty } }}} \right. \kern-\nulldelimiterspace} {\overset{\lower0.5em\hbox{$\smash{\scriptscriptstyle\frown}$}}{T}_{\infty } }}$$ is the temperature difference parameter.

### Motile density equation

21 $$ \overset{\lower0.5em\hbox{$\smash{\scriptscriptstyle\frown}$}}{u} \frac{{\partial \overset{\lower0.5em\hbox{$\smash{\scriptscriptstyle\frown}$}}{n} }}{{\partial \overset{\lower0.5em\hbox{$\smash{\scriptscriptstyle\frown}$}}{x} }} + \overset{\lower0.5em\hbox{$\smash{\scriptscriptstyle\frown}$}}{v} \frac{{\partial \overset{\lower0.5em\hbox{$\smash{\scriptscriptstyle\frown}$}}{n} }}{{\partial \overset{\lower0.5em\hbox{$\smash{\scriptscriptstyle\frown}$}}{y} }} = \overset{\lower0.5em\hbox{$\smash{\scriptscriptstyle\frown}$}}{D}_{m} \frac{{\partial^{2} \overset{\lower0.5em\hbox{$\smash{\scriptscriptstyle\frown}$}}{n} }}{{\partial \overset{\lower0.5em\hbox{$\smash{\scriptscriptstyle\frown}$}}{y}^{2} }} - \frac{{\overset{\lower0.5em\hbox{$\smash{\scriptscriptstyle\frown}$}}{b}_{c} \overset{\lower0.5em\hbox{$\smash{\scriptscriptstyle\frown}$}}{W}_{c} }}{{\overset{\lower0.5em\hbox{$\smash{\scriptscriptstyle\frown}$}}{C}_{w} - \overset{\lower0.5em\hbox{$\smash{\scriptscriptstyle\frown}$}}{C}_{\infty } }}\left( {\frac{\partial }{{\partial \overset{\lower0.5em\hbox{$\smash{\scriptscriptstyle\frown}$}}{y} }}\left( {\overset{\lower0.5em\hbox{$\smash{\scriptscriptstyle\frown}$}}{n} \frac{{\partial \overset{\lower0.5em\hbox{$\smash{\scriptscriptstyle\frown}$}}{C} }}{{\partial \overset{\lower0.5em\hbox{$\smash{\scriptscriptstyle\frown}$}}{y} }}} \right)} \right), $$

with boundary conditions:

22 $$ \begin{gathered} \overset{\lower0.5em\hbox{$\smash{\scriptscriptstyle\frown}$}}{n} = \overset{\lower0.5em\hbox{$\smash{\scriptscriptstyle\frown}$}}{n}_{w} = \overset{\lower0.5em\hbox{$\smash{\scriptscriptstyle\frown}$}}{n}_{0} + \overset{\lower0.5em\hbox{$\smash{\scriptscriptstyle\frown}$}}{e}_{1} \overset{\lower0.5em\hbox{$\smash{\scriptscriptstyle\frown}$}}{x} \,\,at\,\,\overset{\lower0.5em\hbox{$\smash{\scriptscriptstyle\frown}$}}{y} = 0, \hfill \\ \overset{\lower0.5em\hbox{$\smash{\scriptscriptstyle\frown}$}}{n} = \overset{\lower0.5em\hbox{$\smash{\scriptscriptstyle\frown}$}}{n}_{\infty } = \overset{\lower0.5em\hbox{$\smash{\scriptscriptstyle\frown}$}}{n}_{0} + \overset{\lower0.5em\hbox{$\smash{\scriptscriptstyle\frown}$}}{e}_{2} \overset{\lower0.5em\hbox{$\smash{\scriptscriptstyle\frown}$}}{x} \,\,as\,\,\overset{\lower0.5em\hbox{$\smash{\scriptscriptstyle\frown}$}}{y} \to \infty , \hfill \\ \end{gathered} $$

Using (4), (21) and (22) are transformed as:

23 $$ \chi^{\prime\prime} - Lb\left( {f^{\prime}\chi - \chi^{\prime}f + Bf^{\prime}} \right) - Pe\left( {\chi^{\prime}\phi^{\prime} + \left( {\Omega + \chi } \right)\phi^{\prime\prime}} \right) = 0, $$

with transformed boundary conditions:

24 $$ \chi \left( 0 \right) = 1 - B,\,\,\chi \left( \infty \right) = 0, $$

where $$Lb = {{\overset{\lower0.5em\hbox{$\smash{\scriptscriptstyle\frown}$}}{\nu }_{f} } \mathord{\left/ {\vphantom {{\overset{\lower0.5em\hbox{$\smash{\scriptscriptstyle\frown}$}}{\nu }_{f} } {\overset{\lower0.5em\hbox{$\smash{\scriptscriptstyle\frown}$}}{D}_{m} }}} \right. \kern-\nulldelimiterspace} {\overset{\lower0.5em\hbox{$\smash{\scriptscriptstyle\frown}$}}{D}_{m} }}$$ is the bioconvection Lewis number, $$Pe = {{\overset{\lower0.5em\hbox{$\smash{\scriptscriptstyle\frown}$}}{b}_{c} \overset{\lower0.5em\hbox{$\smash{\scriptscriptstyle\frown}$}}{W}_{c} } \mathord{\left/ {\vphantom {{\overset{\lower0.5em\hbox{$\smash{\scriptscriptstyle\frown}$}}{b}_{c} \overset{\lower0.5em\hbox{$\smash{\scriptscriptstyle\frown}$}}{W}_{c} } {\overset{\lower0.5em\hbox{$\smash{\scriptscriptstyle\frown}$}}{D}_{m} }}} \right. \kern-\nulldelimiterspace} {\overset{\lower0.5em\hbox{$\smash{\scriptscriptstyle\frown}$}}{D}_{m} }}$$ is the bioconvection Peclet number, $$\Omega = {{\overset{\lower0.5em\hbox{$\smash{\scriptscriptstyle\frown}$}}{n}_{\infty } } \mathord{\left/ {\vphantom {{\overset{\lower0.5em\hbox{$\smash{\scriptscriptstyle\frown}$}}{n}_{\infty } } {\left( {\overset{\lower0.5em\hbox{$\smash{\scriptscriptstyle\frown}$}}{n}_{w} - \overset{\lower0.5em\hbox{$\smash{\scriptscriptstyle\frown}$}}{n}_{0} } \right)}}} \right. \kern-\nulldelimiterspace} {\left( {\overset{\lower0.5em\hbox{$\smash{\scriptscriptstyle\frown}$}}{n}_{w} - \overset{\lower0.5em\hbox{$\smash{\scriptscriptstyle\frown}$}}{n}_{0} } \right)}}$$ microorganisms’ concentration difference parameter, and $$B = {{\overset{\lower0.5em\hbox{$\smash{\scriptscriptstyle\frown}$}}{e}_{2} } \mathord{\left/ {\vphantom {{\overset{\lower0.5em\hbox{$\smash{\scriptscriptstyle\frown}$}}{e}_{2} } {\overset{\lower0.5em\hbox{$\smash{\scriptscriptstyle\frown}$}}{e}_{1} }}} \right. \kern-\nulldelimiterspace} {\overset{\lower0.5em\hbox{$\smash{\scriptscriptstyle\frown}$}}{e}_{1} }}$$ is the motile density stratification parameter.

The expressions of skin friction, couple stress, Nusselt number, Sherwood number, and density number can be written as:

25 $$ C_{f} = \frac{{\left. {\left[ {\left( {\overset{\lower0.5em\hbox{$\smash{\scriptscriptstyle\frown}$}}{\mu }_{f} + \overset{\lower0.5em\hbox{$\smash{\scriptscriptstyle\frown}$}}{k}_{f} } \right)\frac{{\partial \overset{\lower0.5em\hbox{$\smash{\scriptscriptstyle\frown}$}}{u} }}{{\partial \overset{\lower0.5em\hbox{$\smash{\scriptscriptstyle\frown}$}}{y} }} + \overset{\lower0.5em\hbox{$\smash{\scriptscriptstyle\frown}$}}{k}_{f} \overset{\lower0.5em\hbox{$\smash{\scriptscriptstyle\frown}$}}{N} - \alpha \left( {\overset{\lower0.5em\hbox{$\smash{\scriptscriptstyle\frown}$}}{u} \frac{{\partial^{2} \overset{\lower0.5em\hbox{$\smash{\scriptscriptstyle\frown}$}}{u} }}{{\partial \overset{\lower0.5em\hbox{$\smash{\scriptscriptstyle\frown}$}}{x} \partial \overset{\lower0.5em\hbox{$\smash{\scriptscriptstyle\frown}$}}{y} }} + \overset{\lower0.5em\hbox{$\smash{\scriptscriptstyle\frown}$}}{v} \frac{{\partial^{2} \overset{\lower0.5em\hbox{$\smash{\scriptscriptstyle\frown}$}}{u} }}{{\partial \overset{\lower0.5em\hbox{$\smash{\scriptscriptstyle\frown}$}}{y}^{2} }} + 2\frac{{\partial \overset{\lower0.5em\hbox{$\smash{\scriptscriptstyle\frown}$}}{u} }}{{\partial \overset{\lower0.5em\hbox{$\smash{\scriptscriptstyle\frown}$}}{x} }}\frac{{\partial \overset{\lower0.5em\hbox{$\smash{\scriptscriptstyle\frown}$}}{u} }}{{\partial \overset{\lower0.5em\hbox{$\smash{\scriptscriptstyle\frown}$}}{y} }}} \right)} \right]} \right|_{{\overset{\lower0.5em\hbox{$\smash{\scriptscriptstyle\frown}$}}{y} = 0}} }}{{\frac{1}{2}\overset{\lower0.5em\hbox{$\smash{\scriptscriptstyle\frown}$}}{\rho }_{f} \overset{\lower0.5em\hbox{$\smash{\scriptscriptstyle\frown}$}}{u}_{w}^{2} }}, $$

26 $$ C_{s} = \frac{{\left. {\overset{\lower0.5em\hbox{$\smash{\scriptscriptstyle\frown}$}}{\gamma } \frac{{\partial \overset{\lower0.5em\hbox{$\smash{\scriptscriptstyle\frown}$}}{N} }}{{\partial \overset{\lower0.5em\hbox{$\smash{\scriptscriptstyle\frown}$}}{y} }}} \right|_{{\overset{\lower0.5em\hbox{$\smash{\scriptscriptstyle\frown}$}}{y} = 0}} }}{{\overset{\lower0.5em\hbox{$\smash{\scriptscriptstyle\frown}$}}{\rho }_{f} \overset{\lower0.5em\hbox{$\smash{\scriptscriptstyle\frown}$}}{a}^{2} \overset{\lower0.5em\hbox{$\smash{\scriptscriptstyle\frown}$}}{x}^{3} }}, $$

27 $$ Nu_{x} = \frac{{\left. { - \overset{\lower0.5em\hbox{$\smash{\scriptscriptstyle\frown}$}}{x} \overset{\lower0.5em\hbox{$\smash{\scriptscriptstyle\frown}$}}{k} \left( {1 + \frac{4}{3}\frac{{4\overset{\lower0.5em\hbox{$\smash{\scriptscriptstyle\frown}$}}{\sigma }^{*} \overset{\lower0.5em\hbox{$\smash{\scriptscriptstyle\frown}$}}{T}_{\infty }^{3} }}{{\overset{\lower0.5em\hbox{$\smash{\scriptscriptstyle\frown}$}}{k} \overset{\lower0.5em\hbox{$\smash{\scriptscriptstyle\frown}$}}{k}^{*} }}} \right)\frac{{\partial \overset{\lower0.5em\hbox{$\smash{\scriptscriptstyle\frown}$}}{T} }}{{\partial \overset{\lower0.5em\hbox{$\smash{\scriptscriptstyle\frown}$}}{y} }}} \right|_{{\overset{\lower0.5em\hbox{$\smash{\scriptscriptstyle\frown}$}}{y} = 0}} }}{{\overset{\lower0.5em\hbox{$\smash{\scriptscriptstyle\frown}$}}{k} \left( {\overset{\lower0.5em\hbox{$\smash{\scriptscriptstyle\frown}$}}{T}_{w} - \overset{\lower0.5em\hbox{$\smash{\scriptscriptstyle\frown}$}}{T}_{0} } \right)}}, $$

28 $$ Sh_{x} = \frac{{\left. { - \overset{\lower0.5em\hbox{$\smash{\scriptscriptstyle\frown}$}}{x} \overset{\lower0.5em\hbox{$\smash{\scriptscriptstyle\frown}$}}{D}_{{\overset{\lower0.5em\hbox{$\smash{\scriptscriptstyle\frown}$}}{B} }} \frac{{\partial \overset{\lower0.5em\hbox{$\smash{\scriptscriptstyle\frown}$}}{C} }}{{\partial \overset{\lower0.5em\hbox{$\smash{\scriptscriptstyle\frown}$}}{y} }}} \right|_{{\overset{\lower0.5em\hbox{$\smash{\scriptscriptstyle\frown}$}}{y} = 0}} }}{{\overset{\lower0.5em\hbox{$\smash{\scriptscriptstyle\frown}$}}{D}_{{\overset{\lower0.5em\hbox{$\smash{\scriptscriptstyle\frown}$}}{B} }} \left( {\overset{\lower0.5em\hbox{$\smash{\scriptscriptstyle\frown}$}}{C}_{w} - \overset{\lower0.5em\hbox{$\smash{\scriptscriptstyle\frown}$}}{C}_{0} } \right)}}, $$

and

29 $$ n_{x} = \frac{{\left. { - \overset{\lower0.5em\hbox{$\smash{\scriptscriptstyle\frown}$}}{x} \overset{\lower0.5em\hbox{$\smash{\scriptscriptstyle\frown}$}}{D}_{m} \frac{{\partial \overset{\lower0.5em\hbox{$\smash{\scriptscriptstyle\frown}$}}{n} }}{{\partial \overset{\lower0.5em\hbox{$\smash{\scriptscriptstyle\frown}$}}{y} }}} \right|_{{\overset{\lower0.5em\hbox{$\smash{\scriptscriptstyle\frown}$}}{y} = 0}} }}{{\overset{\lower0.5em\hbox{$\smash{\scriptscriptstyle\frown}$}}{D}_{m} \left( {\overset{\lower0.5em\hbox{$\smash{\scriptscriptstyle\frown}$}}{n}_{w} - \overset{\lower0.5em\hbox{$\smash{\scriptscriptstyle\frown}$}}{n}_{0} } \right)}}, $$

Using (4), (25)-(29) are transformed as:

30 $$ S_{f} = - \left( {1 + K - 3\alpha_{1} } \right)f^{\prime\prime}\left( 0 \right). $$

where $$S_{f} = - \frac{1}{2}\sqrt {{\text{Re}}_{x} } S_{fx}$$.

31 $$ \sqrt {{\text{Re}}_{x} } C_{s} = \left( {1 + \frac{K}{2}} \right)g^{\prime}\left( 0 \right). $$

32 $$ \frac{{Nu_{x} }}{{\sqrt {{\text{Re}}_{x} } }} = - \left( {1 + \frac{4}{3}Rd} \right)\theta^{\prime}\left( 0 \right). $$

33 $$ \frac{{Sh_{x} }}{{\sqrt {{\text{Re}}_{x} } }} = \phi^{\prime}\left( 0 \right). $$

34 $$ \frac{{n_{x} }}{{\sqrt {{\text{Re}}_{x} } }} = - \chi^{\prime}\left( 0 \right). $$

## HAM solution

The linear operators and initial guesses are taken as:

35 $$ L_{f} = f^{\prime\prime\prime} - f^{\prime},\,\,L_{g} = g^{\prime\prime} - g,\,\,L_{\theta } = \theta^{\prime\prime} - \theta ,\,\,L_{\phi } = \phi^{\prime\prime} - \phi ,\,\,L_{\chi } = \chi^{\prime\prime} - \chi , $$

36 $$ f_{0} = 1 - e^{ - \xi } ,\,\,g_{0} = 0,\,\,\theta_{0} = \left( {1 - S} \right)e^{ - \xi } ,\,\,\phi_{0} = \left( {1 - Q} \right)e^{ - \xi } ,\,\,\chi_{0} = \left( {1 - B} \right)e^{ - \xi } , $$

with

37 $$ \begin{gathered} L_{f} \left( {x_{1} + x_{2} e^{\xi } + x_{3} e^{ - \xi } } \right) = 0,\,\,L_{g} \left( {x_{4} e^{\xi } + x_{5} e^{ - \xi } } \right),\,\,L_{\theta } \left( {x_{6} e^{\xi } + x_{7} e^{ - \xi } } \right) = 0, \hfill \\ L_{\phi } \left( {x_{8} e^{\xi } + x_{9} e^{ - \xi } } \right) = 0,\,\,L_{\chi } \left( {x_{10} e^{\xi } + x_{11} e^{ - \xi } } \right) = 0, \hfill \\ \end{gathered} $$

where $x_{1} - x_{11}$ are called constants.

The zeroth order deformation can be written as

38 $$ \left( {1 - \overset{\lower0.5em\hbox{$\smash{\scriptscriptstyle\frown}$}}{p} } \right)\overset{\lower0.5em\hbox{$\smash{\scriptscriptstyle\frown}$}}{L}_{{\overset{\lower0.5em\hbox{$\smash{\scriptscriptstyle\frown}$}}{f} }} \left[ {\overset{\lower0.5em\hbox{$\smash{\scriptscriptstyle\frown}$}}{f} \left( {\xi ;\overset{\lower0.5em\hbox{$\smash{\scriptscriptstyle\frown}$}}{p} } \right) - \overset{\lower0.5em\hbox{$\smash{\scriptscriptstyle\frown}$}}{f}_{0} \left( {\overset{\lower0.5em\hbox{$\smash{\scriptscriptstyle\frown}$}}{p} } \right)} \right] = \overset{\lower0.5em\hbox{$\smash{\scriptscriptstyle\frown}$}}{p} \overset{\lower0.5em\hbox{$\smash{\scriptscriptstyle\frown}$}}{\hbar }_{{\overset{\lower0.5em\hbox{$\smash{\scriptscriptstyle\frown}$}}{f} }} \overset{\lower0.5em\hbox{$\smash{\scriptscriptstyle\frown}$}}{N}_{{\overset{\lower0.5em\hbox{$\smash{\scriptscriptstyle\frown}$}}{f} }} \left[ {\overset{\lower0.5em\hbox{$\smash{\scriptscriptstyle\frown}$}}{f} \left( {\xi ;\overset{\lower0.5em\hbox{$\smash{\scriptscriptstyle\frown}$}}{p} } \right),\overset{\lower0.5em\hbox{$\smash{\scriptscriptstyle\frown}$}}{g} \left( {\xi ;\overset{\lower0.5em\hbox{$\smash{\scriptscriptstyle\frown}$}}{p} } \right),\overset{\lower0.5em\hbox{$\smash{\scriptscriptstyle\frown}$}}{\theta } \left( {\xi ;\overset{\lower0.5em\hbox{$\smash{\scriptscriptstyle\frown}$}}{p} } \right),\overset{\lower0.5em\hbox{$\smash{\scriptscriptstyle\frown}$}}{\phi } \left( {\xi ;\overset{\lower0.5em\hbox{$\smash{\scriptscriptstyle\frown}$}}{p} } \right),\overset{\lower0.5em\hbox{$\smash{\scriptscriptstyle\frown}$}}{\chi } \left( {\xi ;\overset{\lower0.5em\hbox{$\smash{\scriptscriptstyle\frown}$}}{p} } \right)} \right], $$

39 $$ \left( {1 - \overset{\lower0.5em\hbox{$\smash{\scriptscriptstyle\frown}$}}{p} } \right)\overset{\lower0.5em\hbox{$\smash{\scriptscriptstyle\frown}$}}{L}_{{\overset{\lower0.5em\hbox{$\smash{\scriptscriptstyle\frown}$}}{g} }} \left[ {\overset{\lower0.5em\hbox{$\smash{\scriptscriptstyle\frown}$}}{g} \left( {\xi ;\overset{\lower0.5em\hbox{$\smash{\scriptscriptstyle\frown}$}}{p} } \right) - \overset{\lower0.5em\hbox{$\smash{\scriptscriptstyle\frown}$}}{g}_{0} \left( {\overset{\lower0.5em\hbox{$\smash{\scriptscriptstyle\frown}$}}{p} } \right)} \right] = \overset{\lower0.5em\hbox{$\smash{\scriptscriptstyle\frown}$}}{p} \overset{\lower0.5em\hbox{$\smash{\scriptscriptstyle\frown}$}}{\hbar }_{{\overset{\lower0.5em\hbox{$\smash{\scriptscriptstyle\frown}$}}{g} }} \overset{\lower0.5em\hbox{$\smash{\scriptscriptstyle\frown}$}}{N}_{{\overset{\lower0.5em\hbox{$\smash{\scriptscriptstyle\frown}$}}{g} }} \left[ {\overset{\lower0.5em\hbox{$\smash{\scriptscriptstyle\frown}$}}{f} \left( {\xi ;\overset{\lower0.5em\hbox{$\smash{\scriptscriptstyle\frown}$}}{p} } \right),\overset{\lower0.5em\hbox{$\smash{\scriptscriptstyle\frown}$}}{g} \left( {\xi ;\overset{\lower0.5em\hbox{$\smash{\scriptscriptstyle\frown}$}}{p} } \right)} \right], $$

40 $$ \left( {1 - \overset{\lower0.5em\hbox{$\smash{\scriptscriptstyle\frown}$}}{p} } \right)\overset{\lower0.5em\hbox{$\smash{\scriptscriptstyle\frown}$}}{L}_{{\overset{\lower0.5em\hbox{$\smash{\scriptscriptstyle\frown}$}}{\theta } }} \left[ {\overset{\lower0.5em\hbox{$\smash{\scriptscriptstyle\frown}$}}{\theta } \left( {\xi ;\overset{\lower0.5em\hbox{$\smash{\scriptscriptstyle\frown}$}}{p} } \right) - \overset{\lower0.5em\hbox{$\smash{\scriptscriptstyle\frown}$}}{\theta }_{0} \left( {\overset{\lower0.5em\hbox{$\smash{\scriptscriptstyle\frown}$}}{p} } \right)} \right] = \overset{\lower0.5em\hbox{$\smash{\scriptscriptstyle\frown}$}}{p} \overset{\lower0.5em\hbox{$\smash{\scriptscriptstyle\frown}$}}{\hbar }_{{\overset{\lower0.5em\hbox{$\smash{\scriptscriptstyle\frown}$}}{\theta } }} \overset{\lower0.5em\hbox{$\smash{\scriptscriptstyle\frown}$}}{N}_{{\overset{\lower0.5em\hbox{$\smash{\scriptscriptstyle\frown}$}}{\theta } }} \left[ {\overset{\lower0.5em\hbox{$\smash{\scriptscriptstyle\frown}$}}{\theta } \left( {\xi ;\overset{\lower0.5em\hbox{$\smash{\scriptscriptstyle\frown}$}}{p} } \right),\overset{\lower0.5em\hbox{$\smash{\scriptscriptstyle\frown}$}}{f} \left( {\xi ;\overset{\lower0.5em\hbox{$\smash{\scriptscriptstyle\frown}$}}{p} } \right)} \right], $$

41 $$ \left( {1 - \overset{\lower0.5em\hbox{$\smash{\scriptscriptstyle\frown}$}}{p} } \right)\overset{\lower0.5em\hbox{$\smash{\scriptscriptstyle\frown}$}}{L}_{{\overset{\lower0.5em\hbox{$\smash{\scriptscriptstyle\frown}$}}{\phi } }} \left[ {\overset{\lower0.5em\hbox{$\smash{\scriptscriptstyle\frown}$}}{\phi } \left( {\xi ;\overset{\lower0.5em\hbox{$\smash{\scriptscriptstyle\frown}$}}{p} } \right) - \overset{\lower0.5em\hbox{$\smash{\scriptscriptstyle\frown}$}}{\phi }_{0} \left( {\overset{\lower0.5em\hbox{$\smash{\scriptscriptstyle\frown}$}}{p} } \right)} \right] = \overset{\lower0.5em\hbox{$\smash{\scriptscriptstyle\frown}$}}{p} \overset{\lower0.5em\hbox{$\smash{\scriptscriptstyle\frown}$}}{\hbar }_{{\overset{\lower0.5em\hbox{$\smash{\scriptscriptstyle\frown}$}}{\phi } }} \overset{\lower0.5em\hbox{$\smash{\scriptscriptstyle\frown}$}}{N}_{{\overset{\lower0.5em\hbox{$\smash{\scriptscriptstyle\frown}$}}{\phi } }} \left[ {\overset{\lower0.5em\hbox{$\smash{\scriptscriptstyle\frown}$}}{\phi } \left( {\xi ;\overset{\lower0.5em\hbox{$\smash{\scriptscriptstyle\frown}$}}{p} } \right),\overset{\lower0.5em\hbox{$\smash{\scriptscriptstyle\frown}$}}{f} \left( {\xi ;\overset{\lower0.5em\hbox{$\smash{\scriptscriptstyle\frown}$}}{p} } \right),\overset{\lower0.5em\hbox{$\smash{\scriptscriptstyle\frown}$}}{\theta } \left( {\xi ;\overset{\lower0.5em\hbox{$\smash{\scriptscriptstyle\frown}$}}{p} } \right)} \right], $$

42 $$ \left( {1 - \overset{\lower0.5em\hbox{$\smash{\scriptscriptstyle\frown}$}}{p} } \right)\overset{\lower0.5em\hbox{$\smash{\scriptscriptstyle\frown}$}}{L}_{{\overset{\lower0.5em\hbox{$\smash{\scriptscriptstyle\frown}$}}{\chi } }} \left[ {\overset{\lower0.5em\hbox{$\smash{\scriptscriptstyle\frown}$}}{\chi } \left( {\xi ;\overset{\lower0.5em\hbox{$\smash{\scriptscriptstyle\frown}$}}{p} } \right) - \overset{\lower0.5em\hbox{$\smash{\scriptscriptstyle\frown}$}}{\chi }_{0} \left( {\overset{\lower0.5em\hbox{$\smash{\scriptscriptstyle\frown}$}}{p} } \right)} \right] = \overset{\lower0.5em\hbox{$\smash{\scriptscriptstyle\frown}$}}{p} \overset{\lower0.5em\hbox{$\smash{\scriptscriptstyle\frown}$}}{\hbar }_{{\overset{\lower0.5em\hbox{$\smash{\scriptscriptstyle\frown}$}}{\chi } }} \overset{\lower0.5em\hbox{$\smash{\scriptscriptstyle\frown}$}}{N}_{{\overset{\lower0.5em\hbox{$\smash{\scriptscriptstyle\frown}$}}{\chi } }} \left[ {\overset{\lower0.5em\hbox{$\smash{\scriptscriptstyle\frown}$}}{\chi } \left( {\xi ;\overset{\lower0.5em\hbox{$\smash{\scriptscriptstyle\frown}$}}{p} } \right),\overset{\lower0.5em\hbox{$\smash{\scriptscriptstyle\frown}$}}{f} \left( {\xi ;\overset{\lower0.5em\hbox{$\smash{\scriptscriptstyle\frown}$}}{p} } \right),\overset{\lower0.5em\hbox{$\smash{\scriptscriptstyle\frown}$}}{\phi } \left( {\xi ;\overset{\lower0.5em\hbox{$\smash{\scriptscriptstyle\frown}$}}{p} } \right)} \right], $$

43 $$ \overset{\lower0.5em\hbox{$\smash{\scriptscriptstyle\frown}$}}{f} \left( {0;\overset{\lower0.5em\hbox{$\smash{\scriptscriptstyle\frown}$}}{p} } \right) = 0,\,\,\overset{\lower0.5em\hbox{$\smash{\scriptscriptstyle\frown}$}}{f^{\prime}} \left( {0;\overset{\lower0.5em\hbox{$\smash{\scriptscriptstyle\frown}$}}{p} } \right) = 1,\,\,\overset{\lower0.5em\hbox{$\smash{\scriptscriptstyle\frown}$}}{f^{\prime}} \left( {\infty ;\overset{\lower0.5em\hbox{$\smash{\scriptscriptstyle\frown}$}}{p} } \right) = 0, $$

44 $$ \overset{\lower0.5em\hbox{$\smash{\scriptscriptstyle\frown}$}}{g} \left( {0;\overset{\lower0.5em\hbox{$\smash{\scriptscriptstyle\frown}$}}{p} } \right) = 0,\,\,\overset{\lower0.5em\hbox{$\smash{\scriptscriptstyle\frown}$}}{g} \left( {\infty ;\overset{\lower0.5em\hbox{$\smash{\scriptscriptstyle\frown}$}}{p} } \right) = 0, $$

45 $$ \overset{\lower0.5em\hbox{$\smash{\scriptscriptstyle\frown}$}}{\theta } \left( {0;\overset{\lower0.5em\hbox{$\smash{\scriptscriptstyle\frown}$}}{p} } \right) = 1 - S,\,\,\overset{\lower0.5em\hbox{$\smash{\scriptscriptstyle\frown}$}}{\theta } \left( {\infty ;\overset{\lower0.5em\hbox{$\smash{\scriptscriptstyle\frown}$}}{p} } \right) = 0, $$

46 $$ \overset{\lower0.5em\hbox{$\smash{\scriptscriptstyle\frown}$}}{\phi } \left( {0;\overset{\lower0.5em\hbox{$\smash{\scriptscriptstyle\frown}$}}{p} } \right) = 1 - Q,\,\,\overset{\lower0.5em\hbox{$\smash{\scriptscriptstyle\frown}$}}{\phi } \left( {\infty ;\overset{\lower0.5em\hbox{$\smash{\scriptscriptstyle\frown}$}}{p} } \right) = 0, $$

47 $$ \overset{\lower0.5em\hbox{$\smash{\scriptscriptstyle\frown}$}}{\chi } \left( {0;\overset{\lower0.5em\hbox{$\smash{\scriptscriptstyle\frown}$}}{p} } \right) = 1 - B,\,\,\overset{\lower0.5em\hbox{$\smash{\scriptscriptstyle\frown}$}}{\chi } \left( {\infty ;\overset{\lower0.5em\hbox{$\smash{\scriptscriptstyle\frown}$}}{p} } \right) = 0, $$

48 $$ \begin{gathered} \overset{\lower0.5em\hbox{$\smash{\scriptscriptstyle\frown}$}}{N}_{{\overset{\lower0.5em\hbox{$\smash{\scriptscriptstyle\frown}$}}{f} }} \left[ {\overset{\lower0.5em\hbox{$\smash{\scriptscriptstyle\frown}$}}{f} \left( {\xi ;\overset{\lower0.5em\hbox{$\smash{\scriptscriptstyle\frown}$}}{p} } \right),\overset{\lower0.5em\hbox{$\smash{\scriptscriptstyle\frown}$}}{g} \left( {\xi ;\overset{\lower0.5em\hbox{$\smash{\scriptscriptstyle\frown}$}}{p} } \right),\overset{\lower0.5em\hbox{$\smash{\scriptscriptstyle\frown}$}}{\theta } \left( {\xi ;\overset{\lower0.5em\hbox{$\smash{\scriptscriptstyle\frown}$}}{p} } \right),\overset{\lower0.5em\hbox{$\smash{\scriptscriptstyle\frown}$}}{\phi } \left( {\xi ;\overset{\lower0.5em\hbox{$\smash{\scriptscriptstyle\frown}$}}{p} } \right),\overset{\lower0.5em\hbox{$\smash{\scriptscriptstyle\frown}$}}{\chi } \left( {\xi ;\overset{\lower0.5em\hbox{$\smash{\scriptscriptstyle\frown}$}}{p} } \right)} \right] = \left( {1 + K} \right)\frac{{\partial^{3} \overset{\lower0.5em\hbox{$\smash{\scriptscriptstyle\frown}$}}{f} \left( {\xi ;\overset{\lower0.5em\hbox{$\smash{\scriptscriptstyle\frown}$}}{p} } \right)}}{{\partial \xi^{3} }} - \left( {\frac{{\partial \overset{\lower0.5em\hbox{$\smash{\scriptscriptstyle\frown}$}}{f} \left( {\xi ;\overset{\lower0.5em\hbox{$\smash{\scriptscriptstyle\frown}$}}{p} } \right)}}{\partial \xi }} \right)^{2} \hfill \\ + \overset{\lower0.5em\hbox{$\smash{\scriptscriptstyle\frown}$}}{f} \left( {\xi ;\overset{\lower0.5em\hbox{$\smash{\scriptscriptstyle\frown}$}}{p} } \right)\frac{{\partial^{2} \overset{\lower0.5em\hbox{$\smash{\scriptscriptstyle\frown}$}}{f} \left( {\xi ;\overset{\lower0.5em\hbox{$\smash{\scriptscriptstyle\frown}$}}{p} } \right)}}{{\partial \xi^{2} }} - \alpha_{1} \left( {2\frac{{\partial \overset{\lower0.5em\hbox{$\smash{\scriptscriptstyle\frown}$}}{f} \left( {\xi ;\overset{\lower0.5em\hbox{$\smash{\scriptscriptstyle\frown}$}}{p} } \right)}}{\partial \xi }\frac{{\partial^{3} \overset{\lower0.5em\hbox{$\smash{\scriptscriptstyle\frown}$}}{f} \left( {\xi ;\overset{\lower0.5em\hbox{$\smash{\scriptscriptstyle\frown}$}}{p} } \right)}}{{\partial \xi^{3} }} - \overset{\lower0.5em\hbox{$\smash{\scriptscriptstyle\frown}$}}{f} \left( {\xi ;\overset{\lower0.5em\hbox{$\smash{\scriptscriptstyle\frown}$}}{p} } \right)\left( {\frac{{\partial^{2} \overset{\lower0.5em\hbox{$\smash{\scriptscriptstyle\frown}$}}{f} \left( {\xi ;\overset{\lower0.5em\hbox{$\smash{\scriptscriptstyle\frown}$}}{p} } \right)}}{{\partial \xi^{2} }}} \right)^{2} \frac{{\partial^{4} \overset{\lower0.5em\hbox{$\smash{\scriptscriptstyle\frown}$}}{f} \left( {\xi ;\overset{\lower0.5em\hbox{$\smash{\scriptscriptstyle\frown}$}}{p} } \right)}}{{\partial \xi^{4} }}} \right) \hfill \\ + K\frac{{\partial \overset{\lower0.5em\hbox{$\smash{\scriptscriptstyle\frown}$}}{g} \left( {\xi ;\overset{\lower0.5em\hbox{$\smash{\scriptscriptstyle\frown}$}}{p} } \right)}}{\partial \xi } - M\frac{{\partial \overset{\lower0.5em\hbox{$\smash{\scriptscriptstyle\frown}$}}{f} \left( {\xi ;\overset{\lower0.5em\hbox{$\smash{\scriptscriptstyle\frown}$}}{p} } \right)}}{\partial \xi } + M\overline{E} + \lambda \left( {\overset{\lower0.5em\hbox{$\smash{\scriptscriptstyle\frown}$}}{\theta } \left( {\xi ;\overset{\lower0.5em\hbox{$\smash{\scriptscriptstyle\frown}$}}{p} } \right) - Nr\overset{\lower0.5em\hbox{$\smash{\scriptscriptstyle\frown}$}}{\phi } \left( {\xi ;\overset{\lower0.5em\hbox{$\smash{\scriptscriptstyle\frown}$}}{p} } \right) - Rb\overset{\lower0.5em\hbox{$\smash{\scriptscriptstyle\frown}$}}{\chi } \left( {\xi ;\overset{\lower0.5em\hbox{$\smash{\scriptscriptstyle\frown}$}}{p} } \right)} \right), \hfill \\ \end{gathered} $$

49 $$ \begin{gathered} \overset{\lower0.5em\hbox{$\smash{\scriptscriptstyle\frown}$}}{N}_{{\overset{\lower0.5em\hbox{$\smash{\scriptscriptstyle\frown}$}}{g} }} \left[ {\overset{\lower0.5em\hbox{$\smash{\scriptscriptstyle\frown}$}}{g} \left( {\xi ;\overset{\lower0.5em\hbox{$\smash{\scriptscriptstyle\frown}$}}{p} } \right),\overset{\lower0.5em\hbox{$\smash{\scriptscriptstyle\frown}$}}{f} \left( {\xi ;\overset{\lower0.5em\hbox{$\smash{\scriptscriptstyle\frown}$}}{p} } \right)} \right] = \left( {1 + \frac{K}{2}} \right)\frac{{\partial^{2} \overset{\lower0.5em\hbox{$\smash{\scriptscriptstyle\frown}$}}{g} \left( {\xi ;\overset{\lower0.5em\hbox{$\smash{\scriptscriptstyle\frown}$}}{p} } \right)}}{{\partial \xi^{2} }} - \frac{{\partial \overset{\lower0.5em\hbox{$\smash{\scriptscriptstyle\frown}$}}{f} \left( {\xi ;\overset{\lower0.5em\hbox{$\smash{\scriptscriptstyle\frown}$}}{p} } \right)}}{\partial \xi } \times \hfill \\ \overset{\lower0.5em\hbox{$\smash{\scriptscriptstyle\frown}$}}{g} \left( {\xi ;\overset{\lower0.5em\hbox{$\smash{\scriptscriptstyle\frown}$}}{p} } \right) + \overset{\lower0.5em\hbox{$\smash{\scriptscriptstyle\frown}$}}{f} \left( {\xi ;\overset{\lower0.5em\hbox{$\smash{\scriptscriptstyle\frown}$}}{p} } \right)\frac{{\partial \overset{\lower0.5em\hbox{$\smash{\scriptscriptstyle\frown}$}}{g} \left( {\xi ;\overset{\lower0.5em\hbox{$\smash{\scriptscriptstyle\frown}$}}{p} } \right)}}{\partial \xi } - K\left( {2\overset{\lower0.5em\hbox{$\smash{\scriptscriptstyle\frown}$}}{g} \left( {\xi ;\overset{\lower0.5em\hbox{$\smash{\scriptscriptstyle\frown}$}}{p} } \right) + \frac{{\partial^{2} \overset{\lower0.5em\hbox{$\smash{\scriptscriptstyle\frown}$}}{f} \left( {\xi ;\overset{\lower0.5em\hbox{$\smash{\scriptscriptstyle\frown}$}}{p} } \right)}}{{\partial \xi^{2} }}} \right), \hfill \\ \end{gathered} $$

50 $$ \begin{gathered} \overset{\lower0.5em\hbox{$\smash{\scriptscriptstyle\frown}$}}{N}_{{\overset{\lower0.5em\hbox{$\smash{\scriptscriptstyle\frown}$}}{\theta } }} \left[ {\overset{\lower0.5em\hbox{$\smash{\scriptscriptstyle\frown}$}}{\theta } \left( {\xi ;\overset{\lower0.5em\hbox{$\smash{\scriptscriptstyle\frown}$}}{p} } \right),\overset{\lower0.5em\hbox{$\smash{\scriptscriptstyle\frown}$}}{f} \left( {\xi ;\overset{\lower0.5em\hbox{$\smash{\scriptscriptstyle\frown}$}}{p} } \right)} \right] = \left( {1 + \frac{4}{3}Rd} \right)\frac{{\partial^{2} \overset{\lower0.5em\hbox{$\smash{\scriptscriptstyle\frown}$}}{\theta } \left( {\xi ;\overset{\lower0.5em\hbox{$\smash{\scriptscriptstyle\frown}$}}{p} } \right)}}{{\partial \xi^{2} }} + \Pr \overset{\lower0.5em\hbox{$\smash{\scriptscriptstyle\frown}$}}{f} \left( {\xi ;\overset{\lower0.5em\hbox{$\smash{\scriptscriptstyle\frown}$}}{p} } \right)\frac{{\partial \overset{\lower0.5em\hbox{$\smash{\scriptscriptstyle\frown}$}}{\theta } \left( {\xi ;\overset{\lower0.5em\hbox{$\smash{\scriptscriptstyle\frown}$}}{p} } \right)}}{\partial \xi } - \overset{\lower0.5em\hbox{$\smash{\scriptscriptstyle\frown}$}}{\theta } \left( {\xi ;\overset{\lower0.5em\hbox{$\smash{\scriptscriptstyle\frown}$}}{p} } \right) \times \hfill \\ \frac{{\partial \overset{\lower0.5em\hbox{$\smash{\scriptscriptstyle\frown}$}}{f} \left( {\xi ;\overset{\lower0.5em\hbox{$\smash{\scriptscriptstyle\frown}$}}{p} } \right)}}{\partial \xi } - \Pr S\frac{{\partial \overset{\lower0.5em\hbox{$\smash{\scriptscriptstyle\frown}$}}{f} \left( {\xi ;\overset{\lower0.5em\hbox{$\smash{\scriptscriptstyle\frown}$}}{p} } \right)}}{\partial \xi } + \Pr \delta \overset{\lower0.5em\hbox{$\smash{\scriptscriptstyle\frown}$}}{\theta } \left( {\xi ;\overset{\lower0.5em\hbox{$\smash{\scriptscriptstyle\frown}$}}{p} } \right) + M^{2} Ec\left( {\left( {\frac{{\partial \overset{\lower0.5em\hbox{$\smash{\scriptscriptstyle\frown}$}}{f} \left( {\xi ;\overset{\lower0.5em\hbox{$\smash{\scriptscriptstyle\frown}$}}{p} } \right)}}{\partial \xi }} \right)^{2} + E^{2} - 2E\frac{{\partial \overset{\lower0.5em\hbox{$\smash{\scriptscriptstyle\frown}$}}{f} \left( {\xi ;\overset{\lower0.5em\hbox{$\smash{\scriptscriptstyle\frown}$}}{p} } \right)}}{\partial \xi }} \right), \hfill \\ \end{gathered} $$

51 $$ \begin{gathered} \overset{\lower0.5em\hbox{$\smash{\scriptscriptstyle\frown}$}}{N}_{{\overset{\lower0.5em\hbox{$\smash{\scriptscriptstyle\frown}$}}{\phi } }} \left[ {\overset{\lower0.5em\hbox{$\smash{\scriptscriptstyle\frown}$}}{\phi } \left( {\xi ;\overset{\lower0.5em\hbox{$\smash{\scriptscriptstyle\frown}$}}{p} } \right),\overset{\lower0.5em\hbox{$\smash{\scriptscriptstyle\frown}$}}{f} \left( {\xi ;\overset{\lower0.5em\hbox{$\smash{\scriptscriptstyle\frown}$}}{p} } \right),\overset{\lower0.5em\hbox{$\smash{\scriptscriptstyle\frown}$}}{\theta } \left( {\xi ;\overset{\lower0.5em\hbox{$\smash{\scriptscriptstyle\frown}$}}{p} } \right)} \right] = \frac{{\partial^{2} \overset{\lower0.5em\hbox{$\smash{\scriptscriptstyle\frown}$}}{\phi } \left( {\xi ;\overset{\lower0.5em\hbox{$\smash{\scriptscriptstyle\frown}$}}{p} } \right)}}{{\partial \xi^{2} }} + Sc\overset{\lower0.5em\hbox{$\smash{\scriptscriptstyle\frown}$}}{f} \left( {\xi ;\overset{\lower0.5em\hbox{$\smash{\scriptscriptstyle\frown}$}}{p} } \right)\frac{{\partial \overset{\lower0.5em\hbox{$\smash{\scriptscriptstyle\frown}$}}{\phi } \left( {\xi ;\overset{\lower0.5em\hbox{$\smash{\scriptscriptstyle\frown}$}}{p} } \right)}}{\partial \xi } - Sc\overset{\lower0.5em\hbox{$\smash{\scriptscriptstyle\frown}$}}{\phi } \left( {\xi ;\overset{\lower0.5em\hbox{$\smash{\scriptscriptstyle\frown}$}}{p} } \right) \times \hfill \\ \frac{{\partial \overset{\lower0.5em\hbox{$\smash{\scriptscriptstyle\frown}$}}{f} \left( {\xi ;\overset{\lower0.5em\hbox{$\smash{\scriptscriptstyle\frown}$}}{p} } \right)}}{\partial \xi } - ScQ\frac{{\partial \overset{\lower0.5em\hbox{$\smash{\scriptscriptstyle\frown}$}}{f} \left( {\xi ;\overset{\lower0.5em\hbox{$\smash{\scriptscriptstyle\frown}$}}{p} } \right)}}{\partial \xi } - Sc\varpi \left( {1 + \varepsilon \overset{\lower0.5em\hbox{$\smash{\scriptscriptstyle\frown}$}}{\theta } \left( {\xi ;\overset{\lower0.5em\hbox{$\smash{\scriptscriptstyle\frown}$}}{p} } \right)} \right)^{{\overline{n}}} \overset{\lower0.5em\hbox{$\smash{\scriptscriptstyle\frown}$}}{\phi } \left( {\xi ;\overset{\lower0.5em\hbox{$\smash{\scriptscriptstyle\frown}$}}{p} } \right)\exp \left( {\frac{ - E}{{\left( {1 + \varepsilon \theta \overset{\lower0.5em\hbox{$\smash{\scriptscriptstyle\frown}$}}{\theta } \left( {\xi ;\overset{\lower0.5em\hbox{$\smash{\scriptscriptstyle\frown}$}}{p} } \right)} \right)}}} \right), \hfill \\ \end{gathered} $$

52 $$ \begin{gathered} \overset{\lower0.5em\hbox{$\smash{\scriptscriptstyle\frown}$}}{N}_{{\overset{\lower0.5em\hbox{$\smash{\scriptscriptstyle\frown}$}}{\chi } }} \left[ {\overset{\lower0.5em\hbox{$\smash{\scriptscriptstyle\frown}$}}{\chi } \left( {\xi ;\overset{\lower0.5em\hbox{$\smash{\scriptscriptstyle\frown}$}}{p} } \right),\overset{\lower0.5em\hbox{$\smash{\scriptscriptstyle\frown}$}}{f} \left( {\xi ;\overset{\lower0.5em\hbox{$\smash{\scriptscriptstyle\frown}$}}{p} } \right),\overset{\lower0.5em\hbox{$\smash{\scriptscriptstyle\frown}$}}{\phi } \left( {\xi ;\overset{\lower0.5em\hbox{$\smash{\scriptscriptstyle\frown}$}}{p} } \right)} \right] = \frac{{\partial^{2} \overset{\lower0.5em\hbox{$\smash{\scriptscriptstyle\frown}$}}{\chi } \left( {\xi ;\overset{\lower0.5em\hbox{$\smash{\scriptscriptstyle\frown}$}}{p} } \right)}}{{\partial \xi^{2} }} + Lb\overset{\lower0.5em\hbox{$\smash{\scriptscriptstyle\frown}$}}{f} \left( {\xi ;\overset{\lower0.5em\hbox{$\smash{\scriptscriptstyle\frown}$}}{p} } \right)\frac{{\partial \overset{\lower0.5em\hbox{$\smash{\scriptscriptstyle\frown}$}}{\chi } \left( {\xi ;\overset{\lower0.5em\hbox{$\smash{\scriptscriptstyle\frown}$}}{p} } \right)}}{\partial \xi } - B\frac{{\partial \overset{\lower0.5em\hbox{$\smash{\scriptscriptstyle\frown}$}}{f} \left( {\xi ;\overset{\lower0.5em\hbox{$\smash{\scriptscriptstyle\frown}$}}{p} } \right)}}{\partial \xi } \hfill \\ - Lb\overset{\lower0.5em\hbox{$\smash{\scriptscriptstyle\frown}$}}{\chi } \left( {\xi ;\overset{\lower0.5em\hbox{$\smash{\scriptscriptstyle\frown}$}}{p} } \right)\frac{{\partial \overset{\lower0.5em\hbox{$\smash{\scriptscriptstyle\frown}$}}{f} \left( {\xi ;\overset{\lower0.5em\hbox{$\smash{\scriptscriptstyle\frown}$}}{p} } \right)}}{\partial \xi } - Pe\left( {\frac{{\partial \overset{\lower0.5em\hbox{$\smash{\scriptscriptstyle\frown}$}}{\chi } \left( {\xi ;\overset{\lower0.5em\hbox{$\smash{\scriptscriptstyle\frown}$}}{p} } \right)}}{\partial \xi }\frac{{\partial \overset{\lower0.5em\hbox{$\smash{\scriptscriptstyle\frown}$}}{\phi } \left( {\xi ;\overset{\lower0.5em\hbox{$\smash{\scriptscriptstyle\frown}$}}{p} } \right)}}{\partial \xi } + \left( {\Omega + \overset{\lower0.5em\hbox{$\smash{\scriptscriptstyle\frown}$}}{\chi } \left( {\xi ;\overset{\lower0.5em\hbox{$\smash{\scriptscriptstyle\frown}$}}{p} } \right)} \right)\frac{{\partial^{2} \overset{\lower0.5em\hbox{$\smash{\scriptscriptstyle\frown}$}}{\phi } \left( {\xi ;\overset{\lower0.5em\hbox{$\smash{\scriptscriptstyle\frown}$}}{p} } \right)}}{{\partial \xi^{2} }}} \right), \hfill \\ \end{gathered} $$

where $$\overset{\lower0.5em\hbox{$\smash{\scriptscriptstyle\frown}$}}{p} \in \left[ {0,\,\,1} \right]$$ is the embedded parameter and $$\hbar_{{\overset{\lower0.5em\hbox{$\smash{\scriptscriptstyle\frown}$}}{f} }}$$, $$\hbar_{{\overset{\lower0.5em\hbox{$\smash{\scriptscriptstyle\frown}$}}{g} }}$$, $$\hbar_{{\overset{\lower0.5em\hbox{$\smash{\scriptscriptstyle\frown}$}}{\theta } }}$$, $$\hbar_{{\overset{\lower0.5em\hbox{$\smash{\scriptscriptstyle\frown}$}}{\phi } }}$$, and $$\hbar_{{\overset{\lower0.5em\hbox{$\smash{\scriptscriptstyle\frown}$}}{\chi } }}$$ are auxiliary factors.

The $$\overset{\lower0.5em\hbox{$\smash{\scriptscriptstyle\frown}$}}{n}$$th order deformation can be written as:

53 $$ \overset{\lower0.5em\hbox{$\smash{\scriptscriptstyle\frown}$}}{L}_{{\overset{\lower0.5em\hbox{$\smash{\scriptscriptstyle\frown}$}}{f} }} \left[ {\overset{\lower0.5em\hbox{$\smash{\scriptscriptstyle\frown}$}}{f}_{{\overset{\lower0.5em\hbox{$\smash{\scriptscriptstyle\frown}$}}{n} }} \left( \xi \right) - \overline{\chi }_{{\overset{\lower0.5em\hbox{$\smash{\scriptscriptstyle\frown}$}}{n} }} \overset{\lower0.5em\hbox{$\smash{\scriptscriptstyle\frown}$}}{f}_{{\overset{\lower0.5em\hbox{$\smash{\scriptscriptstyle\frown}$}}{n} - 1}} \left( \xi \right)} \right] = \overset{\lower0.5em\hbox{$\smash{\scriptscriptstyle\frown}$}}{\hbar }_{{\overset{\lower0.5em\hbox{$\smash{\scriptscriptstyle\frown}$}}{f} }} \overset{\lower0.5em\hbox{$\smash{\scriptscriptstyle\frown}$}}{R}_{{_{{\overset{\lower0.5em\hbox{$\smash{\scriptscriptstyle\frown}$}}{n} }} }}^{{\overset{\lower0.5em\hbox{$\smash{\scriptscriptstyle\frown}$}}{f} }} , $$

54 $$ \overset{\lower0.5em\hbox{$\smash{\scriptscriptstyle\frown}$}}{L}_{{\overset{\lower0.5em\hbox{$\smash{\scriptscriptstyle\frown}$}}{g} }} \left[ {\overset{\lower0.5em\hbox{$\smash{\scriptscriptstyle\frown}$}}{g}_{{\overset{\lower0.5em\hbox{$\smash{\scriptscriptstyle\frown}$}}{n} }} \left( \xi \right) - \overline{\chi }_{{\overset{\lower0.5em\hbox{$\smash{\scriptscriptstyle\frown}$}}{n} }} \overset{\lower0.5em\hbox{$\smash{\scriptscriptstyle\frown}$}}{g}_{{\overset{\lower0.5em\hbox{$\smash{\scriptscriptstyle\frown}$}}{n} - 1}} \left( \xi \right)} \right] = \overset{\lower0.5em\hbox{$\smash{\scriptscriptstyle\frown}$}}{\hbar }_{{\overset{\lower0.5em\hbox{$\smash{\scriptscriptstyle\frown}$}}{g} }} \overset{\lower0.5em\hbox{$\smash{\scriptscriptstyle\frown}$}}{R}_{{_{{\overset{\lower0.5em\hbox{$\smash{\scriptscriptstyle\frown}$}}{n} }} }}^{{\overset{\lower0.5em\hbox{$\smash{\scriptscriptstyle\frown}$}}{g} }} , $$

55 $$ \overset{\lower0.5em\hbox{$\smash{\scriptscriptstyle\frown}$}}{L}_{{\overset{\lower0.5em\hbox{$\smash{\scriptscriptstyle\frown}$}}{\theta } }} \left[ {\overset{\lower0.5em\hbox{$\smash{\scriptscriptstyle\frown}$}}{\theta }_{{\overset{\lower0.5em\hbox{$\smash{\scriptscriptstyle\frown}$}}{n} }} \left( \xi \right) - \overline{\chi }_{{\overset{\lower0.5em\hbox{$\smash{\scriptscriptstyle\frown}$}}{n} }} \overset{\lower0.5em\hbox{$\smash{\scriptscriptstyle\frown}$}}{\theta }_{{\overset{\lower0.5em\hbox{$\smash{\scriptscriptstyle\frown}$}}{n} - 1}} \left( \xi \right)} \right] = \overset{\lower0.5em\hbox{$\smash{\scriptscriptstyle\frown}$}}{\hbar }_{{\overset{\lower0.5em\hbox{$\smash{\scriptscriptstyle\frown}$}}{\theta } }} \overset{\lower0.5em\hbox{$\smash{\scriptscriptstyle\frown}$}}{R}_{{_{{\overset{\lower0.5em\hbox{$\smash{\scriptscriptstyle\frown}$}}{n} }} }}^{{\overset{\lower0.5em\hbox{$\smash{\scriptscriptstyle\frown}$}}{\theta } }} , $$

56 $$ \overset{\lower0.5em\hbox{$\smash{\scriptscriptstyle\frown}$}}{L}_{{\overset{\lower0.5em\hbox{$\smash{\scriptscriptstyle\frown}$}}{\phi } }} \left[ {\overset{\lower0.5em\hbox{$\smash{\scriptscriptstyle\frown}$}}{\phi }_{{\overset{\lower0.5em\hbox{$\smash{\scriptscriptstyle\frown}$}}{n} }} \left( \xi \right) - \overline{\chi }_{{\overset{\lower0.5em\hbox{$\smash{\scriptscriptstyle\frown}$}}{n} }} \overset{\lower0.5em\hbox{$\smash{\scriptscriptstyle\frown}$}}{\phi }_{{\overset{\lower0.5em\hbox{$\smash{\scriptscriptstyle\frown}$}}{n} - 1}} \left( \xi \right)} \right] = \overset{\lower0.5em\hbox{$\smash{\scriptscriptstyle\frown}$}}{\hbar }_{{\overset{\lower0.5em\hbox{$\smash{\scriptscriptstyle\frown}$}}{\phi } }} \overset{\lower0.5em\hbox{$\smash{\scriptscriptstyle\frown}$}}{R}_{{_{{\overset{\lower0.5em\hbox{$\smash{\scriptscriptstyle\frown}$}}{n} }} }}^{{\overset{\lower0.5em\hbox{$\smash{\scriptscriptstyle\frown}$}}{\phi } }} , $$

57 $$ \overset{\lower0.5em\hbox{$\smash{\scriptscriptstyle\frown}$}}{L}_{{\overset{\lower0.5em\hbox{$\smash{\scriptscriptstyle\frown}$}}{\chi } }} \left[ {\overset{\lower0.5em\hbox{$\smash{\scriptscriptstyle\frown}$}}{\chi }_{{\overset{\lower0.5em\hbox{$\smash{\scriptscriptstyle\frown}$}}{n} }} \left( \xi \right) - \overline{\chi }_{{\overset{\lower0.5em\hbox{$\smash{\scriptscriptstyle\frown}$}}{n} }} \overset{\lower0.5em\hbox{$\smash{\scriptscriptstyle\frown}$}}{\chi }_{{\overset{\lower0.5em\hbox{$\smash{\scriptscriptstyle\frown}$}}{n} - 1}} \left( \xi \right)} \right] = \overset{\lower0.5em\hbox{$\smash{\scriptscriptstyle\frown}$}}{\hbar }_{{\overset{\lower0.5em\hbox{$\smash{\scriptscriptstyle\frown}$}}{\chi } }} \overset{\lower0.5em\hbox{$\smash{\scriptscriptstyle\frown}$}}{R}_{{_{{\overset{\lower0.5em\hbox{$\smash{\scriptscriptstyle\frown}$}}{n} }} }}^{{\overset{\lower0.5em\hbox{$\smash{\scriptscriptstyle\frown}$}}{\chi } }} , $$

58 $$ \overset{\lower0.5em\hbox{$\smash{\scriptscriptstyle\frown}$}}{f}_{{\overset{\lower0.5em\hbox{$\smash{\scriptscriptstyle\frown}$}}{n} }} \left( 0 \right) = 0,\,\,\overset{\lower0.5em\hbox{$\smash{\scriptscriptstyle\frown}$}}{f^{\prime}}_{{\overset{\lower0.5em\hbox{$\smash{\scriptscriptstyle\frown}$}}{n} }} \left( 0 \right) = 1,\,\,\overset{\lower0.5em\hbox{$\smash{\scriptscriptstyle\frown}$}}{f^{\prime}}_{{\overset{\lower0.5em\hbox{$\smash{\scriptscriptstyle\frown}$}}{n} }} \left( \infty \right) = 0, $$

59 $$ \overset{\lower0.5em\hbox{$\smash{\scriptscriptstyle\frown}$}}{g}_{{\overset{\lower0.5em\hbox{$\smash{\scriptscriptstyle\frown}$}}{n} }} \left( 0 \right) = 0,\,\,\overset{\lower0.5em\hbox{$\smash{\scriptscriptstyle\frown}$}}{g}_{{\overset{\lower0.5em\hbox{$\smash{\scriptscriptstyle\frown}$}}{n} }} \left( \infty \right) = 0, $$

60 $$ \overset{\lower0.5em\hbox{$\smash{\scriptscriptstyle\frown}$}}{\theta }_{{\overset{\lower0.5em\hbox{$\smash{\scriptscriptstyle\frown}$}}{n} }} \left( 0 \right) = 0,\,\,\overset{\lower0.5em\hbox{$\smash{\scriptscriptstyle\frown}$}}{\theta }_{{\overset{\lower0.5em\hbox{$\smash{\scriptscriptstyle\frown}$}}{n} }} \left( \infty \right) = 0, $$

61 $$ \overset{\lower0.5em\hbox{$\smash{\scriptscriptstyle\frown}$}}{\phi }_{{\overset{\lower0.5em\hbox{$\smash{\scriptscriptstyle\frown}$}}{n} }} \left( 0 \right) = 0,\,\,\overset{\lower0.5em\hbox{$\smash{\scriptscriptstyle\frown}$}}{\phi }_{{\overset{\lower0.5em\hbox{$\smash{\scriptscriptstyle\frown}$}}{n} }} \left( \infty \right) = 0, $$

62 $$ \overset{\lower0.5em\hbox{$\smash{\scriptscriptstyle\frown}$}}{\chi }_{{\overset{\lower0.5em\hbox{$\smash{\scriptscriptstyle\frown}$}}{n} }} \left( 0 \right) = 0,\,\,\overset{\lower0.5em\hbox{$\smash{\scriptscriptstyle\frown}$}}{\chi }_{{\overset{\lower0.5em\hbox{$\smash{\scriptscriptstyle\frown}$}}{n} }} \left( \infty \right) = 0, $$

63 $$ \begin{aligned} \overset{\lower0.5em\hbox{$\smash{\scriptscriptstyle\frown}$}}{R}_{{_{{\overset{\lower0.5em\hbox{$\smash{\scriptscriptstyle\frown}$}}{n} }} }}^{{\overset{\lower0.5em\hbox{$\smash{\scriptscriptstyle\frown}$}}{f} }} & = \left( {1 + K} \right)\overset{\lower0.5em\hbox{$\smash{\scriptscriptstyle\frown}$}}{f^{\prime\prime\prime}}_{{\overset{\lower0.5em\hbox{$\smash{\scriptscriptstyle\frown}$}}{n} - 1}} + \sum\limits_{{\overset{\lower0.5em\hbox{$\smash{\scriptscriptstyle\frown}$}}{k} = 0}}^{{\overset{\lower0.5em\hbox{$\smash{\scriptscriptstyle\frown}$}}{n} - 1}} {\overset{\lower0.5em\hbox{$\smash{\scriptscriptstyle\frown}$}}{f}_{{\overset{\lower0.5em\hbox{$\smash{\scriptscriptstyle\frown}$}}{n} - 1 - \overset{\lower0.5em\hbox{$\smash{\scriptscriptstyle\frown}$}}{k} }} } \overset{\lower0.5em\hbox{$\smash{\scriptscriptstyle\frown}$}}{f^{\prime\prime}}_{{\overset{\lower0.5em\hbox{$\smash{\scriptscriptstyle\frown}$}}{n} - 1 - \overset{\lower0.5em\hbox{$\smash{\scriptscriptstyle\frown}$}}{k} }} - \alpha_{1} \sum\limits_{{\overset{\lower0.5em\hbox{$\smash{\scriptscriptstyle\frown}$}}{k} = 0}}^{{\overset{\lower0.5em\hbox{$\smash{\scriptscriptstyle\frown}$}}{n} - 1}} {\left( {2\overset{\lower0.5em\hbox{$\smash{\scriptscriptstyle\frown}$}}{f^{\prime}}_{{\overset{\lower0.5em\hbox{$\smash{\scriptscriptstyle\frown}$}}{n} - 1 - \overset{\lower0.5em\hbox{$\smash{\scriptscriptstyle\frown}$}}{k} }} \overset{\lower0.5em\hbox{$\smash{\scriptscriptstyle\frown}$}}{f^{\prime\prime\prime}}_{{\overset{\lower0.5em\hbox{$\smash{\scriptscriptstyle\frown}$}}{k} }} - \overset{\lower0.5em\hbox{$\smash{\scriptscriptstyle\frown}$}}{f^{\prime\prime}}_{{\overset{\lower0.5em\hbox{$\smash{\scriptscriptstyle\frown}$}}{n} - 1 - \overset{\lower0.5em\hbox{$\smash{\scriptscriptstyle\frown}$}}{k} }}^{2} \overset{\lower0.5em\hbox{$\smash{\scriptscriptstyle\frown}$}}{f}_{{\overset{\lower0.5em\hbox{$\smash{\scriptscriptstyle\frown}$}}{n} - j}} \overset{\lower0.5em\hbox{$\smash{\scriptscriptstyle\frown}$}}{f}_{{\overset{\lower0.5em\hbox{$\smash{\scriptscriptstyle\frown}$}}{k} }}^{iv} } \right)} \\ & \quad - \overset{\lower0.5em\hbox{$\smash{\scriptscriptstyle\frown}$}}{f^{\prime}}_{{\overset{\lower0.5em\hbox{$\smash{\scriptscriptstyle\frown}$}}{n} - 1}}^{2} + K\overset{\lower0.5em\hbox{$\smash{\scriptscriptstyle\frown}$}}{g^{\prime}}_{{\overset{\lower0.5em\hbox{$\smash{\scriptscriptstyle\frown}$}}{n} - 1}} - M\overset{\lower0.5em\hbox{$\smash{\scriptscriptstyle\frown}$}}{f^{\prime}}_{{\overset{\lower0.5em\hbox{$\smash{\scriptscriptstyle\frown}$}}{n} - 1}} + M\overline{E} + \lambda \left( {\overset{\lower0.5em\hbox{$\smash{\scriptscriptstyle\frown}$}}{\theta }_{{\overset{\lower0.5em\hbox{$\smash{\scriptscriptstyle\frown}$}}{n} - 1}} - Nr\overset{\lower0.5em\hbox{$\smash{\scriptscriptstyle\frown}$}}{\phi }_{{\overset{\lower0.5em\hbox{$\smash{\scriptscriptstyle\frown}$}}{n} - 1}} - Rb\overset{\lower0.5em\hbox{$\smash{\scriptscriptstyle\frown}$}}{\chi }_{{\overset{\lower0.5em\hbox{$\smash{\scriptscriptstyle\frown}$}}{n} - 1}} } \right) = 0, \\ \end{aligned} $$

64 $$ \overset{\lower0.5em\hbox{$\smash{\scriptscriptstyle\frown}$}}{R}_{{\overset{\lower0.5em\hbox{$\smash{\scriptscriptstyle\frown}$}}{n} }}^{{\overset{\lower0.5em\hbox{$\smash{\scriptscriptstyle\frown}$}}{g} }} = \left( {1 + \frac{K}{2}} \right)\overset{\lower0.5em\hbox{$\smash{\scriptscriptstyle\frown}$}}{g}^{\prime\prime}_{{\overset{\lower0.5em\hbox{$\smash{\scriptscriptstyle\frown}$}}{n} - 1}} - \sum\limits_{{\overset{\lower0.5em\hbox{$\smash{\scriptscriptstyle\frown}$}}{k} = 0}}^{{\overset{\lower0.5em\hbox{$\smash{\scriptscriptstyle\frown}$}}{n} - 1}} {\overset{\lower0.5em\hbox{$\smash{\scriptscriptstyle\frown}$}}{g}_{{\overset{\lower0.5em\hbox{$\smash{\scriptscriptstyle\frown}$}}{n} - 1 - \overset{\lower0.5em\hbox{$\smash{\scriptscriptstyle\frown}$}}{k} }} \overset{\lower0.5em\hbox{$\smash{\scriptscriptstyle\frown}$}}{f}^{\prime}_{{\overset{\lower0.5em\hbox{$\smash{\scriptscriptstyle\frown}$}}{k} }} } + \sum\limits_{{\overset{\lower0.5em\hbox{$\smash{\scriptscriptstyle\frown}$}}{k} = 0}}^{{\overset{\lower0.5em\hbox{$\smash{\scriptscriptstyle\frown}$}}{n} - 1}} {\overset{\lower0.5em\hbox{$\smash{\scriptscriptstyle\frown}$}}{f}_{{\overset{\lower0.5em\hbox{$\smash{\scriptscriptstyle\frown}$}}{n} - 1 - \overset{\lower0.5em\hbox{$\smash{\scriptscriptstyle\frown}$}}{k} }} \overset{\lower0.5em\hbox{$\smash{\scriptscriptstyle\frown}$}}{g^{\prime}}_{{\overset{\lower0.5em\hbox{$\smash{\scriptscriptstyle\frown}$}}{k} }} } - K(2\overset{\lower0.5em\hbox{$\smash{\scriptscriptstyle\frown}$}}{g}^{\prime\prime}_{{\overset{\lower0.5em\hbox{$\smash{\scriptscriptstyle\frown}$}}{n} - 1}} + \overset{\lower0.5em\hbox{$\smash{\scriptscriptstyle\frown}$}}{f^{\prime\prime}}_{{\overset{\lower0.5em\hbox{$\smash{\scriptscriptstyle\frown}$}}{n} - 1}} ), $$

65 $$ \overset{\lower0.5em\hbox{$\smash{\scriptscriptstyle\frown}$}}{R}_{{\overset{\lower0.5em\hbox{$\smash{\scriptscriptstyle\frown}$}}{n} }}^{{\overset{\lower0.5em\hbox{$\smash{\scriptscriptstyle\frown}$}}{\theta } }} = \left( {1 + \frac{3}{4}Rd} \right)\overset{\lower0.5em\hbox{$\smash{\scriptscriptstyle\frown}$}}{\theta }^{\prime\prime}_{{\overset{\lower0.5em\hbox{$\smash{\scriptscriptstyle\frown}$}}{n} - 1}} - \Pr \sum\limits_{{\overset{\lower0.5em\hbox{$\smash{\scriptscriptstyle\frown}$}}{k} = 0}}^{{\overset{\lower0.5em\hbox{$\smash{\scriptscriptstyle\frown}$}}{n} - 1}} {\overset{\lower0.5em\hbox{$\smash{\scriptscriptstyle\frown}$}}{\theta }_{{\overset{\lower0.5em\hbox{$\smash{\scriptscriptstyle\frown}$}}{n} - 1 - \overset{\lower0.5em\hbox{$\smash{\scriptscriptstyle\frown}$}}{k} }} \overset{\lower0.5em\hbox{$\smash{\scriptscriptstyle\frown}$}}{f}^{\prime}_{{\overset{\lower0.5em\hbox{$\smash{\scriptscriptstyle\frown}$}}{k} }} } + \sum\limits_{{\overset{\lower0.5em\hbox{$\smash{\scriptscriptstyle\frown}$}}{k} = 0}}^{{\overset{\lower0.5em\hbox{$\smash{\scriptscriptstyle\frown}$}}{n} - 1}} {\overset{\lower0.5em\hbox{$\smash{\scriptscriptstyle\frown}$}}{f}_{{\overset{\lower0.5em\hbox{$\smash{\scriptscriptstyle\frown}$}}{n} - 1 - \overset{\lower0.5em\hbox{$\smash{\scriptscriptstyle\frown}$}}{k} }} \overset{\lower0.5em\hbox{$\smash{\scriptscriptstyle\frown}$}}{\theta }^{\prime}_{{\overset{\lower0.5em\hbox{$\smash{\scriptscriptstyle\frown}$}}{k} }} } - \Pr S\overset{\lower0.5em\hbox{$\smash{\scriptscriptstyle\frown}$}}{f}^{\prime}_{{\overset{\lower0.5em\hbox{$\smash{\scriptscriptstyle\frown}$}}{n} - 1}} + \Pr \delta \overset{\lower0.5em\hbox{$\smash{\scriptscriptstyle\frown}$}}{\theta }_{{\overset{\lower0.5em\hbox{$\smash{\scriptscriptstyle\frown}$}}{n} - 1}} + M^{2} Ec\left( {\overset{\lower0.5em\hbox{$\smash{\scriptscriptstyle\frown}$}}{f}_{{\overset{\lower0.5em\hbox{$\smash{\scriptscriptstyle\frown}$}}{n} - 1}}^{\prime 2} + E^{2} - 2E\overset{\lower0.5em\hbox{$\smash{\scriptscriptstyle\frown}$}}{f}^{\prime}_{{\overset{\lower0.5em\hbox{$\smash{\scriptscriptstyle\frown}$}}{n} - 1}} } \right), $$

66 $$ \overset{\lower0.5em\hbox{$\smash{\scriptscriptstyle\frown}$}}{R}_{{_{{\overset{\lower0.5em\hbox{$\smash{\scriptscriptstyle\frown}$}}{n} }} }}^{{\overset{\lower0.5em\hbox{$\smash{\scriptscriptstyle\frown}$}}{\phi } }} = \left( {1 + \frac{3}{4}Rd} \right)\overset{\lower0.5em\hbox{$\smash{\scriptscriptstyle\frown}$}}{\phi }^{\prime\prime}_{{\overset{\lower0.5em\hbox{$\smash{\scriptscriptstyle\frown}$}}{n} - 1}} - Sc\sum\limits_{{\overset{\lower0.5em\hbox{$\smash{\scriptscriptstyle\frown}$}}{k} = 0}}^{{\overset{\lower0.5em\hbox{$\smash{\scriptscriptstyle\frown}$}}{n} - 1}} {\overset{\lower0.5em\hbox{$\smash{\scriptscriptstyle\frown}$}}{\phi }_{{\overset{\lower0.5em\hbox{$\smash{\scriptscriptstyle\frown}$}}{n} - 1 - \overset{\lower0.5em\hbox{$\smash{\scriptscriptstyle\frown}$}}{k} }} } \overset{\lower0.5em\hbox{$\smash{\scriptscriptstyle\frown}$}}{f}^{\prime}_{{\overset{\lower0.5em\hbox{$\smash{\scriptscriptstyle\frown}$}}{k} }} + Sc\sum\limits_{{\overset{\lower0.5em\hbox{$\smash{\scriptscriptstyle\frown}$}}{k} = 0}}^{{\overset{\lower0.5em\hbox{$\smash{\scriptscriptstyle\frown}$}}{n} - 1}} {\overset{\lower0.5em\hbox{$\smash{\scriptscriptstyle\frown}$}}{f}_{{\overset{\lower0.5em\hbox{$\smash{\scriptscriptstyle\frown}$}}{n} - 1 - \overset{\lower0.5em\hbox{$\smash{\scriptscriptstyle\frown}$}}{k} }} } \overset{\lower0.5em\hbox{$\smash{\scriptscriptstyle\frown}$}}{\phi }^{\prime}_{{\overset{\lower0.5em\hbox{$\smash{\scriptscriptstyle\frown}$}}{k} }} - ScQ\overset{\lower0.5em\hbox{$\smash{\scriptscriptstyle\frown}$}}{f}^{\prime}_{{\overset{\lower0.5em\hbox{$\smash{\scriptscriptstyle\frown}$}}{n} - 1}} - Sc\varpi \left( {1 + \varepsilon \overset{\lower0.5em\hbox{$\smash{\scriptscriptstyle\frown}$}}{\theta }_{{\overset{\lower0.5em\hbox{$\smash{\scriptscriptstyle\frown}$}}{n} - 1}} } \right)^{{\overline{n}}} \overset{\lower0.5em\hbox{$\smash{\scriptscriptstyle\frown}$}}{\phi }_{{\overset{\lower0.5em\hbox{$\smash{\scriptscriptstyle\frown}$}}{n} - 1}} \exp \left( {\frac{ - E}{{\left( {1 + \varepsilon \theta_{{\overset{\lower0.5em\hbox{$\smash{\scriptscriptstyle\frown}$}}{n} - 1}} } \right)}}} \right), $$

67 $$ \overset{\lower0.5em\hbox{$\smash{\scriptscriptstyle\frown}$}}{R}_{{_{{\overset{\lower0.5em\hbox{$\smash{\scriptscriptstyle\frown}$}}{n} }} }}^{{\overset{\lower0.5em\hbox{$\smash{\scriptscriptstyle\frown}$}}{\chi } }} = \overset{\lower0.5em\hbox{$\smash{\scriptscriptstyle\frown}$}}{\chi }^{\prime\prime}_{{\overset{\lower0.5em\hbox{$\smash{\scriptscriptstyle\frown}$}}{n} - 1}} - Lb\sum\limits_{{\overset{\lower0.5em\hbox{$\smash{\scriptscriptstyle\frown}$}}{k} = 0}}^{{\overset{\lower0.5em\hbox{$\smash{\scriptscriptstyle\frown}$}}{n} - 1}} {\overset{\lower0.5em\hbox{$\smash{\scriptscriptstyle\frown}$}}{\chi }_{{\overset{\lower0.5em\hbox{$\smash{\scriptscriptstyle\frown}$}}{n} - 1 - \overset{\lower0.5em\hbox{$\smash{\scriptscriptstyle\frown}$}}{k} }} } \overset{\lower0.5em\hbox{$\smash{\scriptscriptstyle\frown}$}}{f}^{\prime}_{{\overset{\lower0.5em\hbox{$\smash{\scriptscriptstyle\frown}$}}{k} }} + Lb\sum\limits_{{\overset{\lower0.5em\hbox{$\smash{\scriptscriptstyle\frown}$}}{k} = 0}}^{{\overset{\lower0.5em\hbox{$\smash{\scriptscriptstyle\frown}$}}{n} - 1}} {\overset{\lower0.5em\hbox{$\smash{\scriptscriptstyle\frown}$}}{f}_{{\overset{\lower0.5em\hbox{$\smash{\scriptscriptstyle\frown}$}}{n} - 1 - \overset{\lower0.5em\hbox{$\smash{\scriptscriptstyle\frown}$}}{k} }} } \overset{\lower0.5em\hbox{$\smash{\scriptscriptstyle\frown}$}}{\chi }_{{\overset{\lower0.5em\hbox{$\smash{\scriptscriptstyle\frown}$}}{k} }} - LbB\overset{\lower0.5em\hbox{$\smash{\scriptscriptstyle\frown}$}}{f}^{\prime}_{{\overset{\lower0.5em\hbox{$\smash{\scriptscriptstyle\frown}$}}{n} - 1}} - Pe\left( {\sum\limits_{{\overset{\lower0.5em\hbox{$\smash{\scriptscriptstyle\frown}$}}{k} = 0}}^{{\overset{\lower0.5em\hbox{$\smash{\scriptscriptstyle\frown}$}}{n} - 1}} {\overset{\lower0.5em\hbox{$\smash{\scriptscriptstyle\frown}$}}{\chi }^{\prime}_{{\overset{\lower0.5em\hbox{$\smash{\scriptscriptstyle\frown}$}}{n} - 1 - \overset{\lower0.5em\hbox{$\smash{\scriptscriptstyle\frown}$}}{k} }} } \overset{\lower0.5em\hbox{$\smash{\scriptscriptstyle\frown}$}}{\phi^{\prime}}_{{\overset{\lower0.5em\hbox{$\smash{\scriptscriptstyle\frown}$}}{k} }} + \left( {\Omega + \overset{\lower0.5em\hbox{$\smash{\scriptscriptstyle\frown}$}}{\chi }_{{\overset{\lower0.5em\hbox{$\smash{\scriptscriptstyle\frown}$}}{n} - 1}} } \right)\overset{\lower0.5em\hbox{$\smash{\scriptscriptstyle\frown}$}}{\phi^{\prime}}_{{\overset{\lower0.5em\hbox{$\smash{\scriptscriptstyle\frown}$}}{n} - 1}} } \right). $$

For $$\overset{\lower0.5em\hbox{$\smash{\scriptscriptstyle\frown}$}}{p} = 0$$ and $$\overset{\lower0.5em\hbox{$\smash{\scriptscriptstyle\frown}$}}{p} = 1$$, we can write:

68 $$ \overset{\lower0.5em\hbox{$\smash{\scriptscriptstyle\frown}$}}{f} \left( {\xi ,0} \right) = \overset{\lower0.5em\hbox{$\smash{\scriptscriptstyle\frown}$}}{f}_{0} \left( \xi \right),\,\,\,\overset{\lower0.5em\hbox{$\smash{\scriptscriptstyle\frown}$}}{f} \left( {\xi ,1} \right) = \overset{\lower0.5em\hbox{$\smash{\scriptscriptstyle\frown}$}}{f} \left( \xi \right), $$

69 $$ \overset{\lower0.5em\hbox{$\smash{\scriptscriptstyle\frown}$}}{g} \left( {\xi ,0} \right) = \overset{\lower0.5em\hbox{$\smash{\scriptscriptstyle\frown}$}}{g}_{0} \left( \xi \right),\,\,\,\overset{\lower0.5em\hbox{$\smash{\scriptscriptstyle\frown}$}}{g} \left( {\xi ,1} \right) = \overset{\lower0.5em\hbox{$\smash{\scriptscriptstyle\frown}$}}{g} \left( \xi \right), $$

70 $$ \overset{\lower0.5em\hbox{$\smash{\scriptscriptstyle\frown}$}}{\theta } \left( {\xi ,0} \right) = \overset{\lower0.5em\hbox{$\smash{\scriptscriptstyle\frown}$}}{\theta }_{0} \left( \xi \right),\,\,\,\overset{\lower0.5em\hbox{$\smash{\scriptscriptstyle\frown}$}}{\theta } \left( {\xi ,1} \right) = \overset{\lower0.5em\hbox{$\smash{\scriptscriptstyle\frown}$}}{\theta } \left( \xi \right), $$

71 $$ \overset{\lower0.5em\hbox{$\smash{\scriptscriptstyle\frown}$}}{\phi } \left( {\xi ,0} \right) = \overset{\lower0.5em\hbox{$\smash{\scriptscriptstyle\frown}$}}{\phi }_{0} \left( \xi \right),\,\,\,\overset{\lower0.5em\hbox{$\smash{\scriptscriptstyle\frown}$}}{\phi } \left( {\xi ,1} \right) = \overset{\lower0.5em\hbox{$\smash{\scriptscriptstyle\frown}$}}{\phi } \left( \xi \right), $$

72 $$ \overset{\lower0.5em\hbox{$\smash{\scriptscriptstyle\frown}$}}{\chi } \left( {\xi ,0} \right) = \overset{\lower0.5em\hbox{$\smash{\scriptscriptstyle\frown}$}}{\chi }_{0} \left( \xi \right),\,\,\,\overset{\lower0.5em\hbox{$\smash{\scriptscriptstyle\frown}$}}{\chi } \left( {\xi ,1} \right) = \overset{\lower0.5em\hbox{$\smash{\scriptscriptstyle\frown}$}}{\chi } \left( \xi \right), $$

When $$\overset{\lower0.5em\hbox{$\smash{\scriptscriptstyle\frown}$}}{p}$$ varies from 0 to 1, the solutions varies from initial to final. Using Taylor’s series to expand the solutions: i.e.

73 $$ \overset{\lower0.5em\hbox{$\smash{\scriptscriptstyle\frown}$}}{f} \left( {\xi ,\overset{\lower0.5em\hbox{$\smash{\scriptscriptstyle\frown}$}}{p} } \right) = \overset{\lower0.5em\hbox{$\smash{\scriptscriptstyle\frown}$}}{f}_{0} \left( \xi \right) + \sum\limits_{{\overset{\lower0.5em\hbox{$\smash{\scriptscriptstyle\frown}$}}{n} = 1}}^{\infty } {\overset{\lower0.5em\hbox{$\smash{\scriptscriptstyle\frown}$}}{f}_{{\overset{\lower0.5em\hbox{$\smash{\scriptscriptstyle\frown}$}}{n} }} \left( \xi \right)\overset{\lower0.5em\hbox{$\smash{\scriptscriptstyle\frown}$}}{p}^{{\overset{\lower0.5em\hbox{$\smash{\scriptscriptstyle\frown}$}}{n} }} } ,\,\,\,\overset{\lower0.5em\hbox{$\smash{\scriptscriptstyle\frown}$}}{f}_{{\overset{\lower0.5em\hbox{$\smash{\scriptscriptstyle\frown}$}}{n} }} \left( \xi \right) = \frac{1}{{\overset{\lower0.5em\hbox{$\smash{\scriptscriptstyle\frown}$}}{n} !}}\left. {\frac{{\partial^{{\overset{\lower0.5em\hbox{$\smash{\scriptscriptstyle\frown}$}}{n} }} \overset{\lower0.5em\hbox{$\smash{\scriptscriptstyle\frown}$}}{f} \left( {\xi ;\overset{\lower0.5em\hbox{$\smash{\scriptscriptstyle\frown}$}}{p} } \right)}}{{\partial \overset{\lower0.5em\hbox{$\smash{\scriptscriptstyle\frown}$}}{p}^{{\overset{\lower0.5em\hbox{$\smash{\scriptscriptstyle\frown}$}}{n} }} }}} \right|_{{\overset{\lower0.5em\hbox{$\smash{\scriptscriptstyle\frown}$}}{p} = 0}} . $$

74 $$ \overset{\lower0.5em\hbox{$\smash{\scriptscriptstyle\frown}$}}{g} \left( {\xi ,\overset{\lower0.5em\hbox{$\smash{\scriptscriptstyle\frown}$}}{p} } \right) = \overset{\lower0.5em\hbox{$\smash{\scriptscriptstyle\frown}$}}{g}_{0} \left( \xi \right) + \sum\limits_{{\overset{\lower0.5em\hbox{$\smash{\scriptscriptstyle\frown}$}}{n} = 1}}^{\infty } {\overset{\lower0.5em\hbox{$\smash{\scriptscriptstyle\frown}$}}{g}_{{\overset{\lower0.5em\hbox{$\smash{\scriptscriptstyle\frown}$}}{n} }} \left( \xi \right)\overset{\lower0.5em\hbox{$\smash{\scriptscriptstyle\frown}$}}{p}^{{\overset{\lower0.5em\hbox{$\smash{\scriptscriptstyle\frown}$}}{n} }} } ,\,\,\,\overset{\lower0.5em\hbox{$\smash{\scriptscriptstyle\frown}$}}{g}_{{\overset{\lower0.5em\hbox{$\smash{\scriptscriptstyle\frown}$}}{n} }} \left( \xi \right) = \frac{1}{{\overset{\lower0.5em\hbox{$\smash{\scriptscriptstyle\frown}$}}{n} !}}\left. {\frac{{\partial^{{\overset{\lower0.5em\hbox{$\smash{\scriptscriptstyle\frown}$}}{n} }} \overset{\lower0.5em\hbox{$\smash{\scriptscriptstyle\frown}$}}{g} \left( {\xi ;\overset{\lower0.5em\hbox{$\smash{\scriptscriptstyle\frown}$}}{p} } \right)}}{{\partial \overset{\lower0.5em\hbox{$\smash{\scriptscriptstyle\frown}$}}{p}^{{\overset{\lower0.5em\hbox{$\smash{\scriptscriptstyle\frown}$}}{n} }} }}} \right|_{{\overset{\lower0.5em\hbox{$\smash{\scriptscriptstyle\frown}$}}{p} = 0}} . $$

75 $$ \overset{\lower0.5em\hbox{$\smash{\scriptscriptstyle\frown}$}}{\theta } \left( {\xi ,\overset{\lower0.5em\hbox{$\smash{\scriptscriptstyle\frown}$}}{p} } \right) = \overset{\lower0.5em\hbox{$\smash{\scriptscriptstyle\frown}$}}{\theta }_{0} \left( \xi \right) + \sum\limits_{{\overset{\lower0.5em\hbox{$\smash{\scriptscriptstyle\frown}$}}{n} = 1}}^{\infty } {\overset{\lower0.5em\hbox{$\smash{\scriptscriptstyle\frown}$}}{\theta }_{{\overset{\lower0.5em\hbox{$\smash{\scriptscriptstyle\frown}$}}{n} }} \left( \xi \right)\overset{\lower0.5em\hbox{$\smash{\scriptscriptstyle\frown}$}}{p}^{{\overset{\lower0.5em\hbox{$\smash{\scriptscriptstyle\frown}$}}{n} }} } ,\,\,\,\overset{\lower0.5em\hbox{$\smash{\scriptscriptstyle\frown}$}}{\theta }_{{\overset{\lower0.5em\hbox{$\smash{\scriptscriptstyle\frown}$}}{n} }} \left( \xi \right) = \frac{1}{{\overset{\lower0.5em\hbox{$\smash{\scriptscriptstyle\frown}$}}{n} !}}\left. {\frac{{\partial^{{\overset{\lower0.5em\hbox{$\smash{\scriptscriptstyle\frown}$}}{n} }} \overset{\lower0.5em\hbox{$\smash{\scriptscriptstyle\frown}$}}{\theta } \left( {\xi ;\overset{\lower0.5em\hbox{$\smash{\scriptscriptstyle\frown}$}}{p} } \right)}}{{\partial \overset{\lower0.5em\hbox{$\smash{\scriptscriptstyle\frown}$}}{p}^{{\overset{\lower0.5em\hbox{$\smash{\scriptscriptstyle\frown}$}}{n} }} }}} \right|_{{\overset{\lower0.5em\hbox{$\smash{\scriptscriptstyle\frown}$}}{p} = 0}} . $$

76 $$ \overset{\lower0.5em\hbox{$\smash{\scriptscriptstyle\frown}$}}{\phi } \left( {\xi ,\overset{\lower0.5em\hbox{$\smash{\scriptscriptstyle\frown}$}}{p} } \right) = \overset{\lower0.5em\hbox{$\smash{\scriptscriptstyle\frown}$}}{\phi }_{0} \left( \xi \right) + \sum\limits_{{\overset{\lower0.5em\hbox{$\smash{\scriptscriptstyle\frown}$}}{n} = 1}}^{\infty } {\overset{\lower0.5em\hbox{$\smash{\scriptscriptstyle\frown}$}}{\phi }_{{\overset{\lower0.5em\hbox{$\smash{\scriptscriptstyle\frown}$}}{n} }} \left( \xi \right)\overset{\lower0.5em\hbox{$\smash{\scriptscriptstyle\frown}$}}{p}^{{\overset{\lower0.5em\hbox{$\smash{\scriptscriptstyle\frown}$}}{n} }} } ,\,\,\,\overset{\lower0.5em\hbox{$\smash{\scriptscriptstyle\frown}$}}{\phi }_{{\overset{\lower0.5em\hbox{$\smash{\scriptscriptstyle\frown}$}}{n} }} \left( \xi \right) = \frac{1}{{\overset{\lower0.5em\hbox{$\smash{\scriptscriptstyle\frown}$}}{n} !}}\left. {\frac{{\partial^{{\overset{\lower0.5em\hbox{$\smash{\scriptscriptstyle\frown}$}}{n} }} \overset{\lower0.5em\hbox{$\smash{\scriptscriptstyle\frown}$}}{\phi } \left( {\xi ;\overset{\lower0.5em\hbox{$\smash{\scriptscriptstyle\frown}$}}{p} } \right)}}{{\partial \overset{\lower0.5em\hbox{$\smash{\scriptscriptstyle\frown}$}}{p}^{{\overset{\lower0.5em\hbox{$\smash{\scriptscriptstyle\frown}$}}{n} }} }}} \right|_{{\overset{\lower0.5em\hbox{$\smash{\scriptscriptstyle\frown}$}}{p} = 0}} . $$

77 $$ \overset{\lower0.5em\hbox{$\smash{\scriptscriptstyle\frown}$}}{\chi } \left( {\xi ,\overset{\lower0.5em\hbox{$\smash{\scriptscriptstyle\frown}$}}{p} } \right) = \overset{\lower0.5em\hbox{$\smash{\scriptscriptstyle\frown}$}}{\chi }_{0} \left( \xi \right) + \sum\limits_{{\overset{\lower0.5em\hbox{$\smash{\scriptscriptstyle\frown}$}}{n} = 1}}^{\infty } {\overset{\lower0.5em\hbox{$\smash{\scriptscriptstyle\frown}$}}{\chi }_{{\overset{\lower0.5em\hbox{$\smash{\scriptscriptstyle\frown}$}}{n} }} \left( \xi \right)\overset{\lower0.5em\hbox{$\smash{\scriptscriptstyle\frown}$}}{p}^{{\overset{\lower0.5em\hbox{$\smash{\scriptscriptstyle\frown}$}}{n} }} } ,\,\,\,\overset{\lower0.5em\hbox{$\smash{\scriptscriptstyle\frown}$}}{\chi }_{{\overset{\lower0.5em\hbox{$\smash{\scriptscriptstyle\frown}$}}{n} }} \left( \xi \right) = \frac{1}{{\overset{\lower0.5em\hbox{$\smash{\scriptscriptstyle\frown}$}}{n} !}}\left. {\frac{{\partial^{{\overset{\lower0.5em\hbox{$\smash{\scriptscriptstyle\frown}$}}{n} }} \overset{\lower0.5em\hbox{$\smash{\scriptscriptstyle\frown}$}}{\chi } \left( {\xi ;\overset{\lower0.5em\hbox{$\smash{\scriptscriptstyle\frown}$}}{p} } \right)}}{{\partial \overset{\lower0.5em\hbox{$\smash{\scriptscriptstyle\frown}$}}{p}^{{\overset{\lower0.5em\hbox{$\smash{\scriptscriptstyle\frown}$}}{n} }} }}} \right|_{{\overset{\lower0.5em\hbox{$\smash{\scriptscriptstyle\frown}$}}{p} = 0}} . $$

The series (73–77) converges by choosing $$\overset{\lower0.5em\hbox{$\smash{\scriptscriptstyle\frown}$}}{p} = 1$$, i.e.

78 $$ \overset{\lower0.5em\hbox{$\smash{\scriptscriptstyle\frown}$}}{f} \left( \xi \right) = \overset{\lower0.5em\hbox{$\smash{\scriptscriptstyle\frown}$}}{f}_{0} \left( \xi \right) + \sum\limits_{{\overset{\lower0.5em\hbox{$\smash{\scriptscriptstyle\frown}$}}{n} = 1}}^{\infty } {\overset{\lower0.5em\hbox{$\smash{\scriptscriptstyle\frown}$}}{f}_{{\overset{\lower0.5em\hbox{$\smash{\scriptscriptstyle\frown}$}}{n} }} \left( \xi \right)} , $$

79 $$ \overset{\lower0.5em\hbox{$\smash{\scriptscriptstyle\frown}$}}{g} \left( \xi \right) = \overset{\lower0.5em\hbox{$\smash{\scriptscriptstyle\frown}$}}{g}_{0} \left( \xi \right) + \sum\limits_{{\overset{\lower0.5em\hbox{$\smash{\scriptscriptstyle\frown}$}}{n} = 1}}^{\infty } {\overset{\lower0.5em\hbox{$\smash{\scriptscriptstyle\frown}$}}{g}_{{\overset{\lower0.5em\hbox{$\smash{\scriptscriptstyle\frown}$}}{n} }} \left( \xi \right)} , $$

80 $$ \overset{\lower0.5em\hbox{$\smash{\scriptscriptstyle\frown}$}}{\theta } \left( \xi \right) = \overset{\lower0.5em\hbox{$\smash{\scriptscriptstyle\frown}$}}{\theta }_{0} \left( \xi \right) + \sum\limits_{{\overset{\lower0.5em\hbox{$\smash{\scriptscriptstyle\frown}$}}{n} = 1}}^{\infty } {\overset{\lower0.5em\hbox{$\smash{\scriptscriptstyle\frown}$}}{\theta }_{{\overset{\lower0.5em\hbox{$\smash{\scriptscriptstyle\frown}$}}{n} }} \left( \xi \right)} , $$

81 $$ \overset{\lower0.5em\hbox{$\smash{\scriptscriptstyle\frown}$}}{\phi } \left( \xi \right) = \overset{\lower0.5em\hbox{$\smash{\scriptscriptstyle\frown}$}}{\phi }_{0} \left( \xi \right) + \sum\limits_{{\overset{\lower0.5em\hbox{$\smash{\scriptscriptstyle\frown}$}}{n} = 1}}^{\infty } {\overset{\lower0.5em\hbox{$\smash{\scriptscriptstyle\frown}$}}{\phi }_{{\overset{\lower0.5em\hbox{$\smash{\scriptscriptstyle\frown}$}}{n} }} \left( \xi \right)} , $$

82 $$ \overset{\lower0.5em\hbox{$\smash{\scriptscriptstyle\frown}$}}{\chi } \left( \xi \right) = \overset{\lower0.5em\hbox{$\smash{\scriptscriptstyle\frown}$}}{\chi }_{0} \left( \xi \right) + \sum\limits_{{\overset{\lower0.5em\hbox{$\smash{\scriptscriptstyle\frown}$}}{n} = 1}}^{\infty } {\overset{\lower0.5em\hbox{$\smash{\scriptscriptstyle\frown}$}}{\chi }_{{\overset{\lower0.5em\hbox{$\smash{\scriptscriptstyle\frown}$}}{n} }} \left( \xi \right)} , $$

### HAM convergence

**Figure 2:**
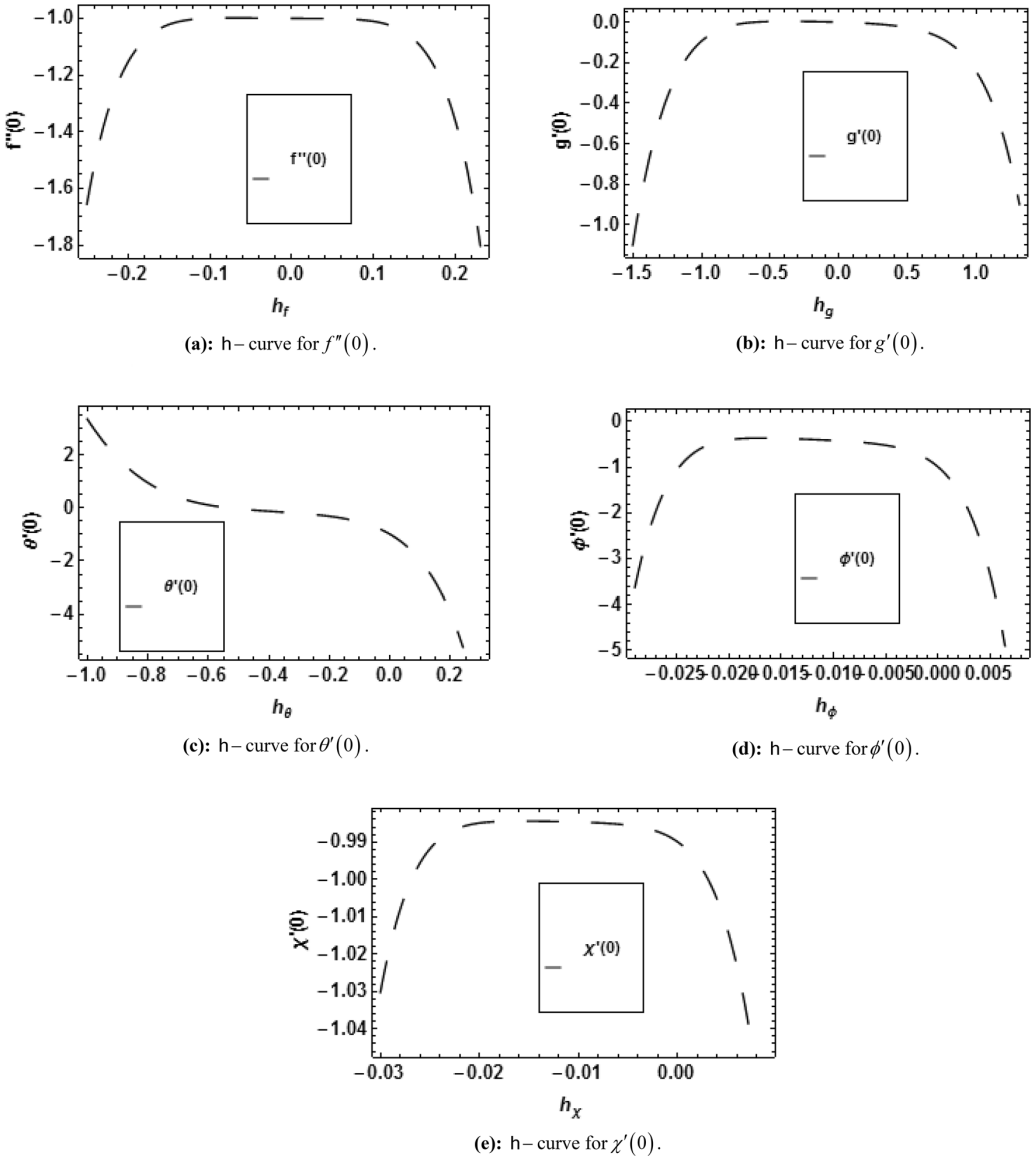
**(a)** $\hbar -$ curve for $f^{\prime\prime}\left( 0 \right)$. **(b)** $\hbar -$ curve for $g^{\prime}\left( 0 \right)$. **(c)** $\hbar -$ curve for $\theta^{\prime}\left( 0 \right)$. **(d)** $\hbar -$ curve for $\phi^{\prime}\left( 0 \right)$. **(e)** $\hbar -$ curve for $\chi^{\prime}\left( 0 \right)$.

HAM solution is operated to investigate the analytical solution of the present analysis. HAM is associated with the axillary parameters $\hbar_{f}$, $\hbar_{g}$, $\hbar_{\theta }$, $\hbar_{\phi }$, and $\hbar_{\chi }$. These parameters are responsible to utilize and control the convergence area of the series solutions. The convergence areas of the velocity, microrotation, temperature, concentration, and motile density functions are $- 0.1 \le \hbar_{f} \le 0.1$, $- 0.75 \le \hbar_{g} \le 0.5$, $- 0.5 \le \hbar_{\theta } \le - 0.2$, $- 0.020 \le \hbar_{\phi } \le - 0.005$ and $- 0.02 \le \hbar_{\chi } \le 0.05$ (see Fig. 2a–e).

## Validation of the present analysis

An analytical scheme called HAM has been focused for the solution of the flow problem. The present analysis has been compared with previously published results by Wakif et al.^50^, and Eldabe and Ouaf^52^ and found a great agreement (see Tables 1 and 2).

**Table 1:** Numerical comparison of the present values with Wakif et al.^50^ when $\lambda = Nr = Rb = K = \overline{E} = 0.0$.

$\alpha_{1}$	$M$	$- f^{\prime\prime}\left( 0 \right)$	
		^50^	Present values
0.0	0.0	1.000000000	1.000000000
0.0	16.0	4.123105625	4.123105625
0.1	16.0	4.346134936	4.346134936
0.2	16.0	4.609772228	4.609772228
0.3	16.0	4.928053803	4.928053803
0.1	4.0	1.054092553	1.054092553
0.1	9.0	3.333333333	3.333333333
0.1	16.0	4.346134936	4.346134936

**Table 2:** Numerical comparison of the present values with Hsiao^5^ when $\alpha_{1} = 0.0$.

$M$	$K$	$g^{\prime}\left( 0 \right)$	
		^52^	Present values
0.0	0.1	0.09500	0.095000
0.5	0.1	0.10509	0.105091
1.0	0.1	0.11212	0.112131
0.1	0.0	0.00000	0.000000
0.1	0.5	0.21116	0.211169
0.1	2.0	0.35855	0.358562

## Results and discussion

**Figure 3:**
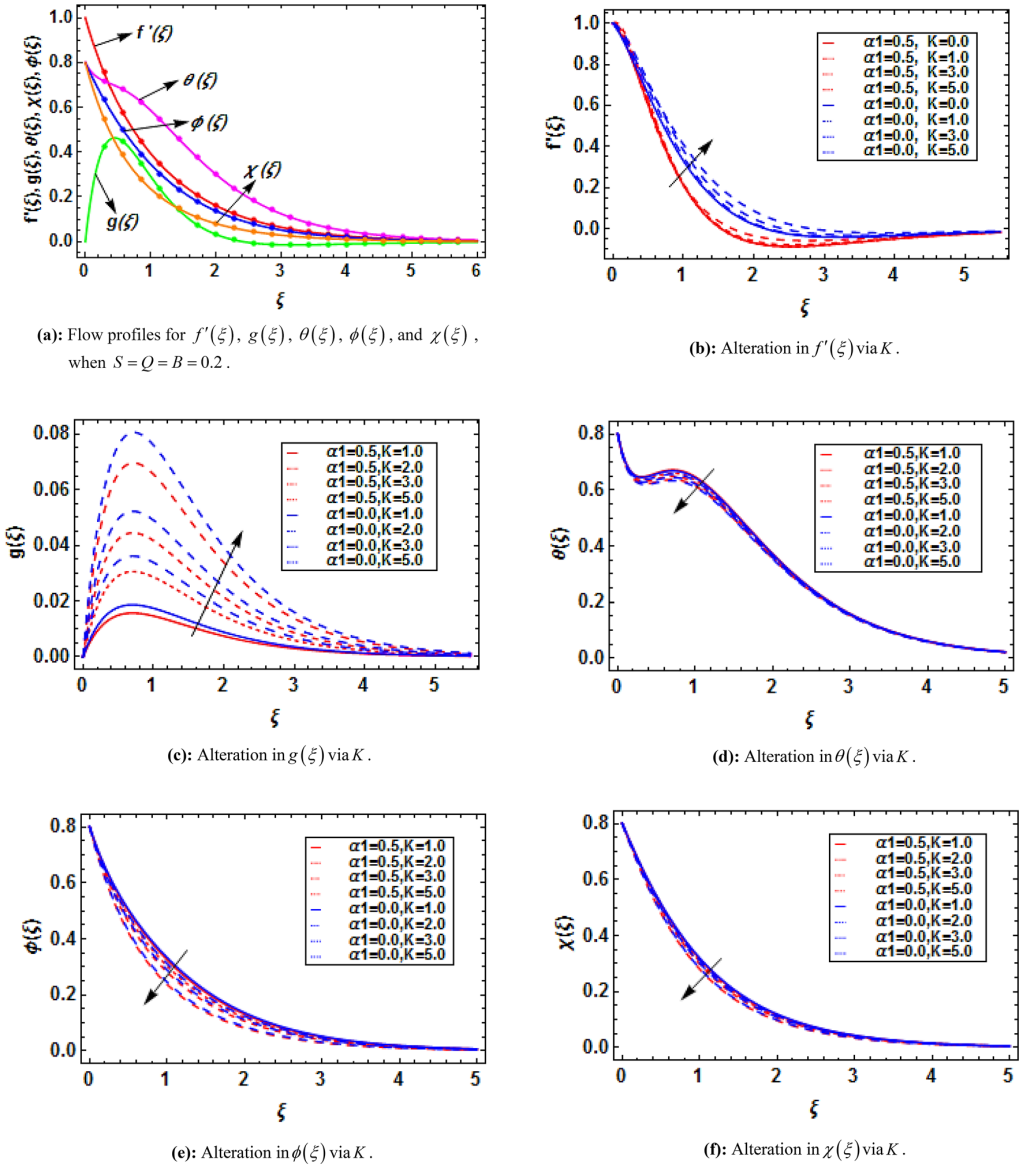
**(a)** Flow profiles for $f^{\prime}\left( \xi \right)$, $g\left( \xi \right)$, $\theta \left( \xi \right)$, $\phi \left( \xi \right)$, and $\chi \left( \xi \right)$ , when $S = Q = B = 0.2$. **(b)** Alteration in $f^{\prime}\left( \xi \right)$ via $K$. **(c)** Alteration in $g\left( \xi \right)$ via $K$. **(d)** Alteration in $\theta \left( \xi \right)$ via $K$. **(e)** Alteration in $\phi \left( \xi \right)$ via $K$. **(f)** Alteration in $\chi \left( \xi \right)$ via $K$.

**Figure 4:**
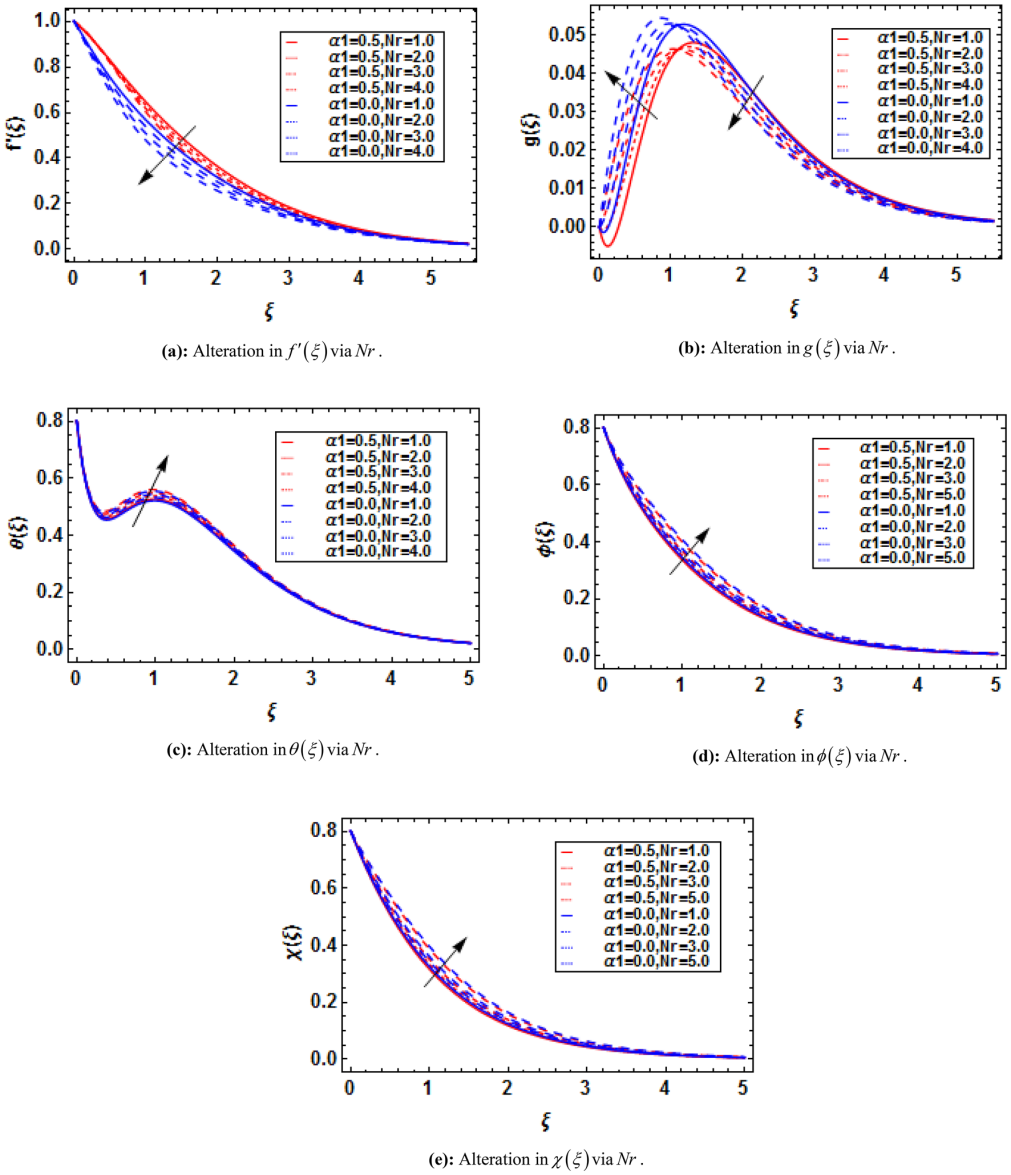
**(a)** Alteration in $f^{\prime}\left( \xi \right)$ via $Nr$. **(b)** Alteration in $g\left( \xi \right)$ via $Nr$. **(c)** Alteration in $\theta \left( \xi \right)$ via $Nr$. **(d)** Alteration in $\phi \left( \xi \right)$ via $Nr$. **(e)** Alteration in $\chi \left( \xi \right)$ via $Nr$.

**Figure 5:**
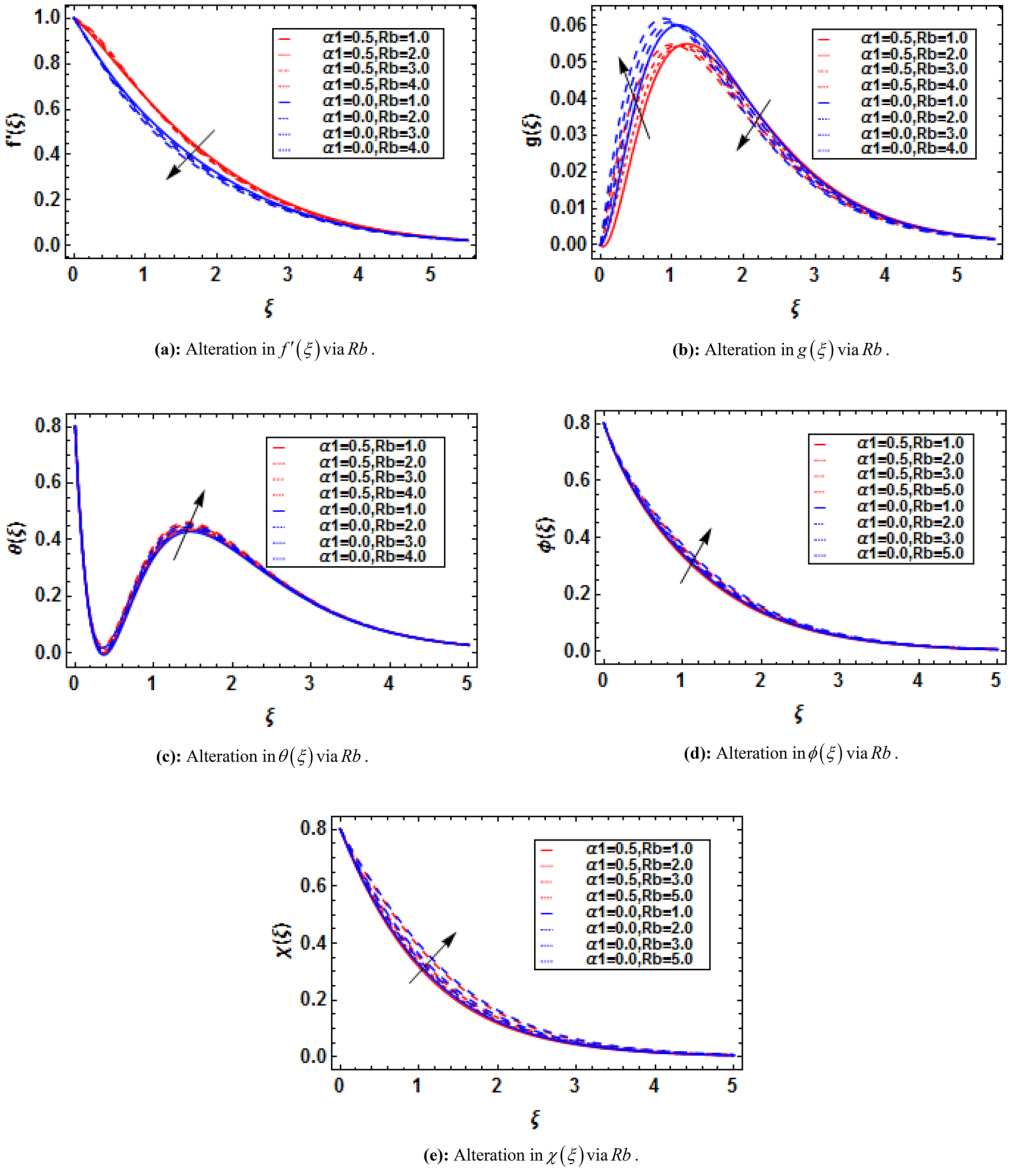
**(a)** Alteration in $f^{\prime}\left( \xi \right)$ via $Rb$. **(b)** Alteration in $g\left( \xi \right)$ via $Rb$. **(c)** Alteration in $\theta \left( \xi \right)$ via $Rb$. **(d)** Alteration in $\phi \left( \xi \right)$ via $Rb$. **(e)** Alteration in $\chi \left( \xi \right)$ via $Rb$.

**Figure 6:**
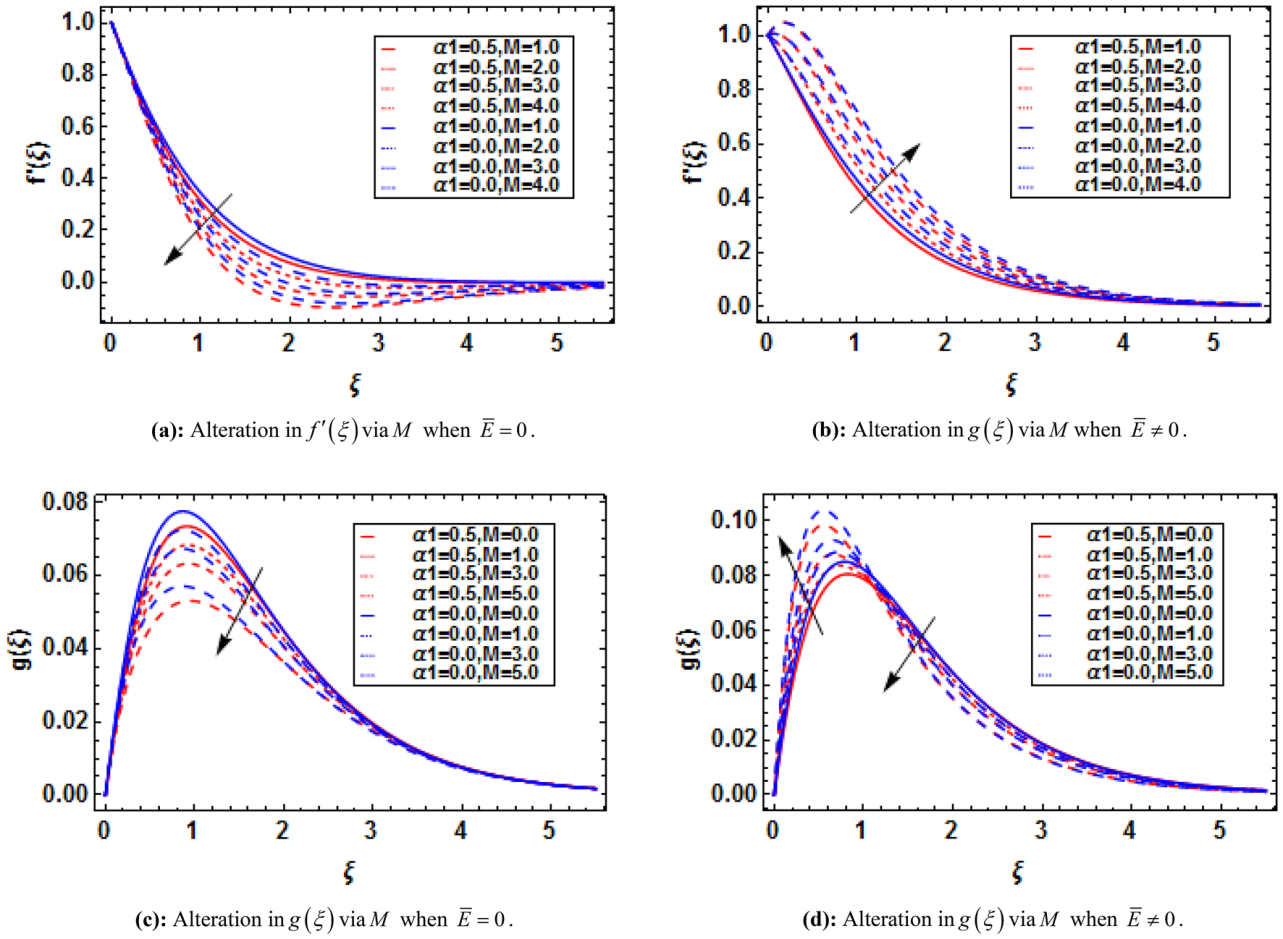
**(a)** Alteration in $f^{\prime}\left( \xi \right)$ via $M$ when $\overline{E} = 0$. **(b)** Alteration in $g\left( \xi \right)$ via $M$ when $\overline{E} \ne 0$. **(c)** Alteration in $g\left( \xi \right)$ via $M$ when $\overline{E} = 0$. **(d)** Alteration in $g\left( \xi \right)$ via $M$ when $\overline{E} \ne 0$.

**Figure 7:**
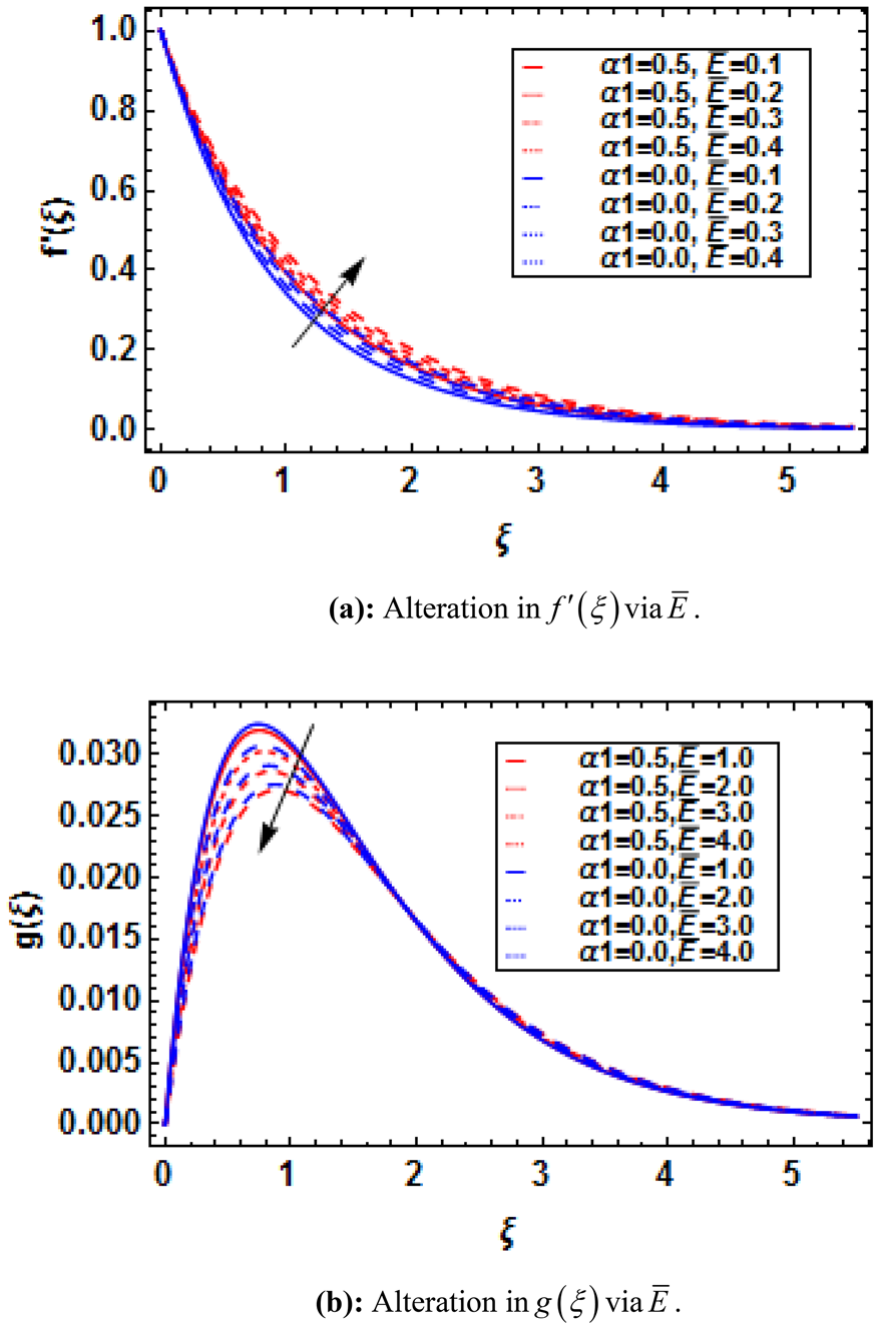
**(a)** Alteration in $f^{\prime}\left( \xi \right)$ via $\overline{E}$. **(b)** Alteration in $g\left( \xi \right)$ via $\overline{E}$.

**Figure 8:**
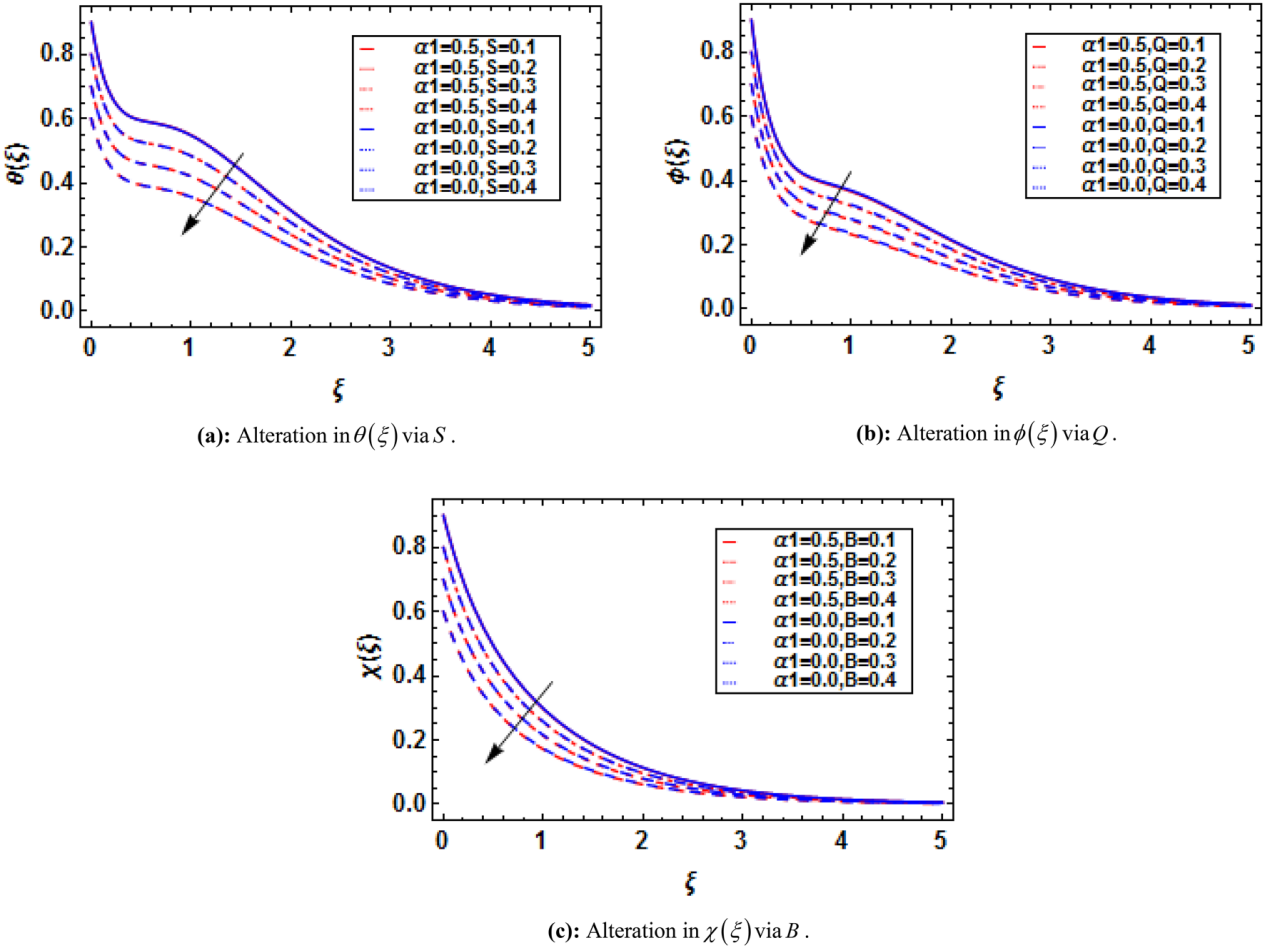
**(a)** Alteration in $\theta \left( \xi \right)$ via $S$. **(b)** Alteration in $\phi \left( \xi \right)$ via $Q$. **(c)** Alteration in $\chi \left( \xi \right)$ via $B$.

**Figure 9:**
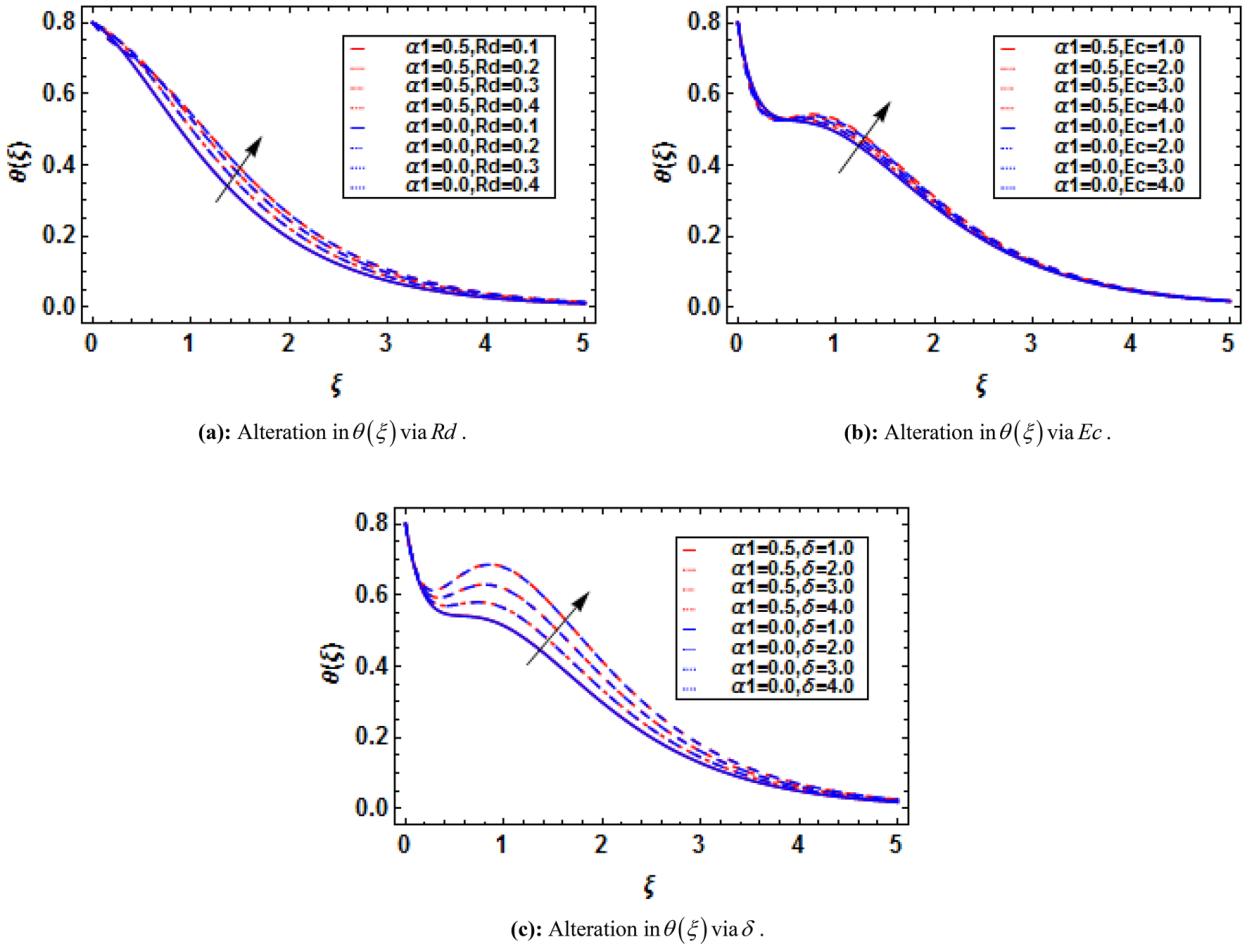
**(a)** Alteration in $\theta \left( \xi \right)$ via $Rd$. **(b)** Alteration in $\theta \left( \xi \right)$ via $Ec$. **(c)** Alteration in $\theta \left( \xi \right)$ via $\delta$.

**Figure 10:**
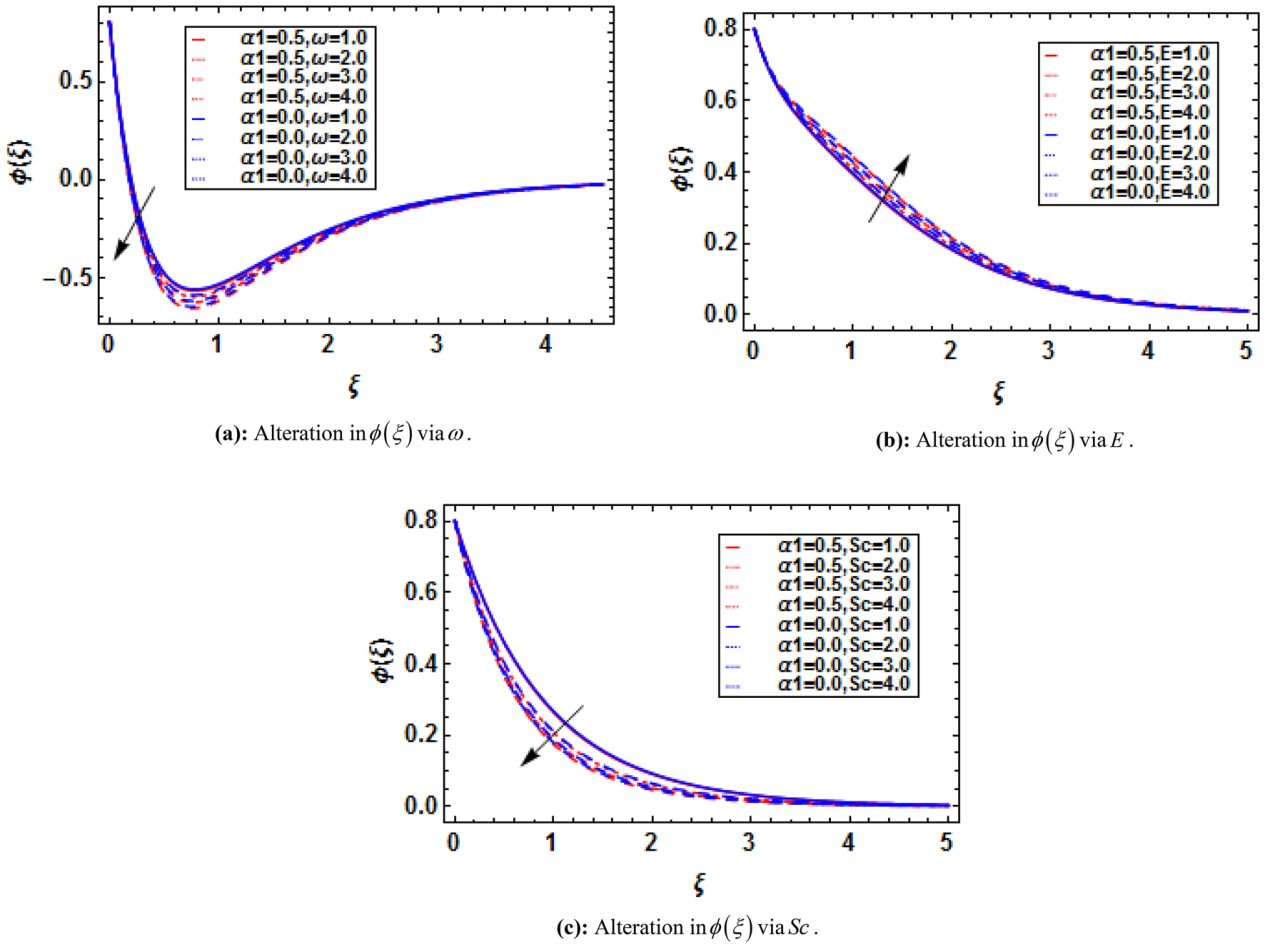
**(a)** Alteration in $\phi \left( \xi \right)$ via $\omega$. **(b)** Alteration in $\phi \left( \xi \right)$ via $E$. **(c)** Alteration in $\phi \left( \xi \right)$ via $Sc$.

**Figure 11:**
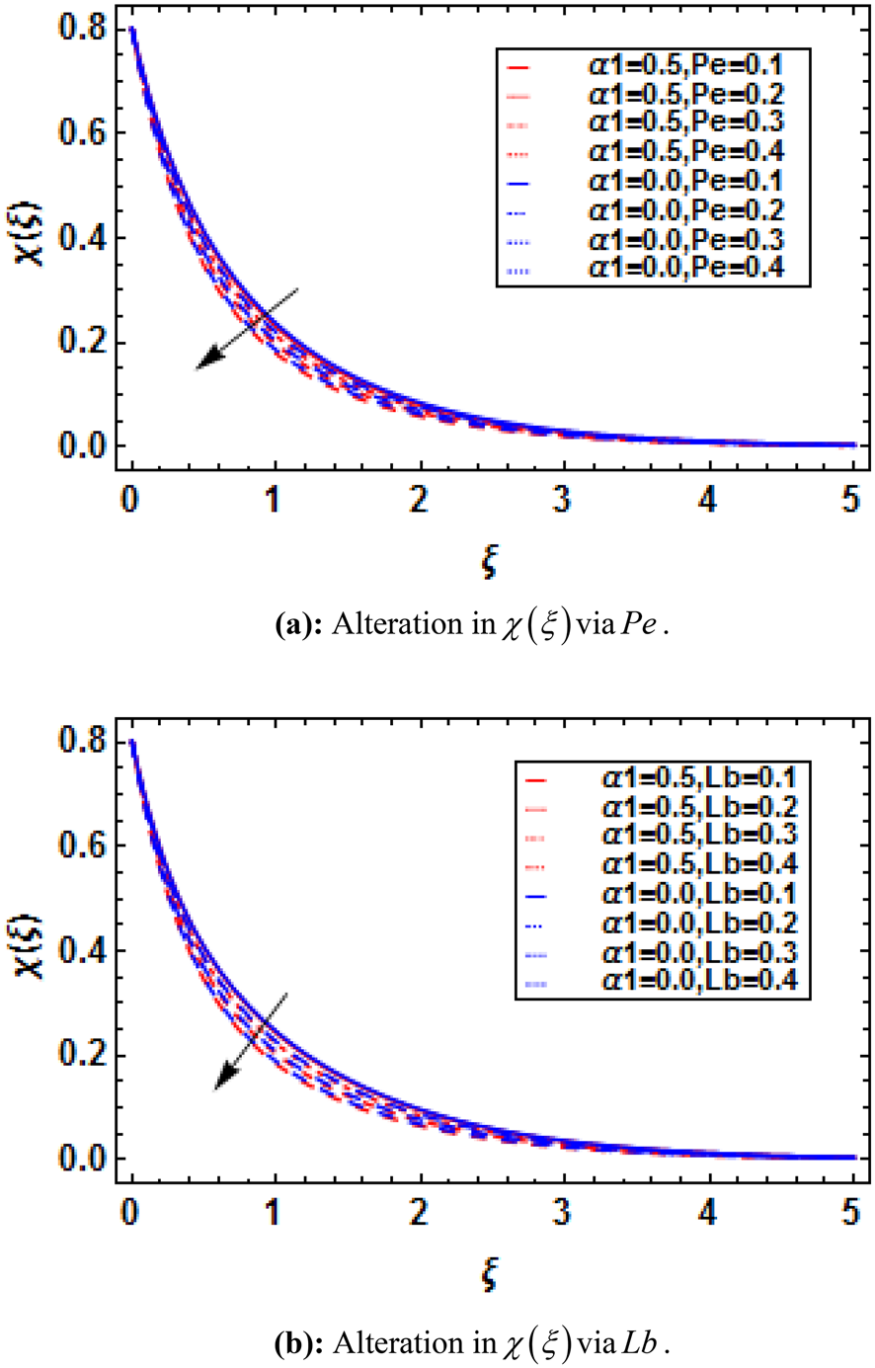
**(a)** Alteration in $\chi \left( \xi \right)$ via $Pe$. **(b)** Alteration in $\chi \left( \xi \right)$ via $Lb$.

**Figure 12:**
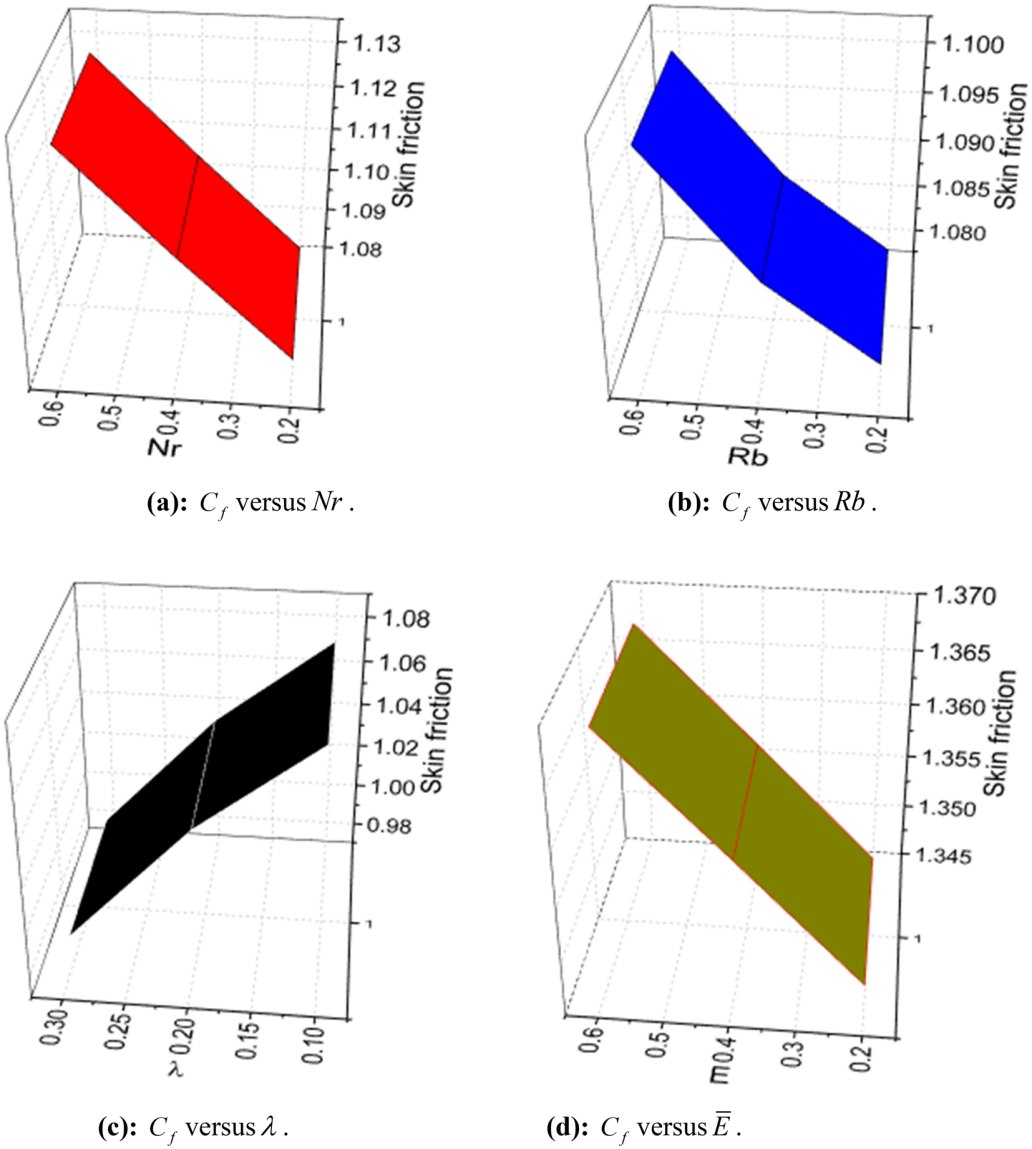
**(a)** $C_{f}$ versus $Nr$. **(b)** $C_{f}$ versus $Rb$. **(c)** $C_{f}$ versus $\lambda$. **(d)** $C_{f}$ versus $\overline{E}$.

**Figure 13:**
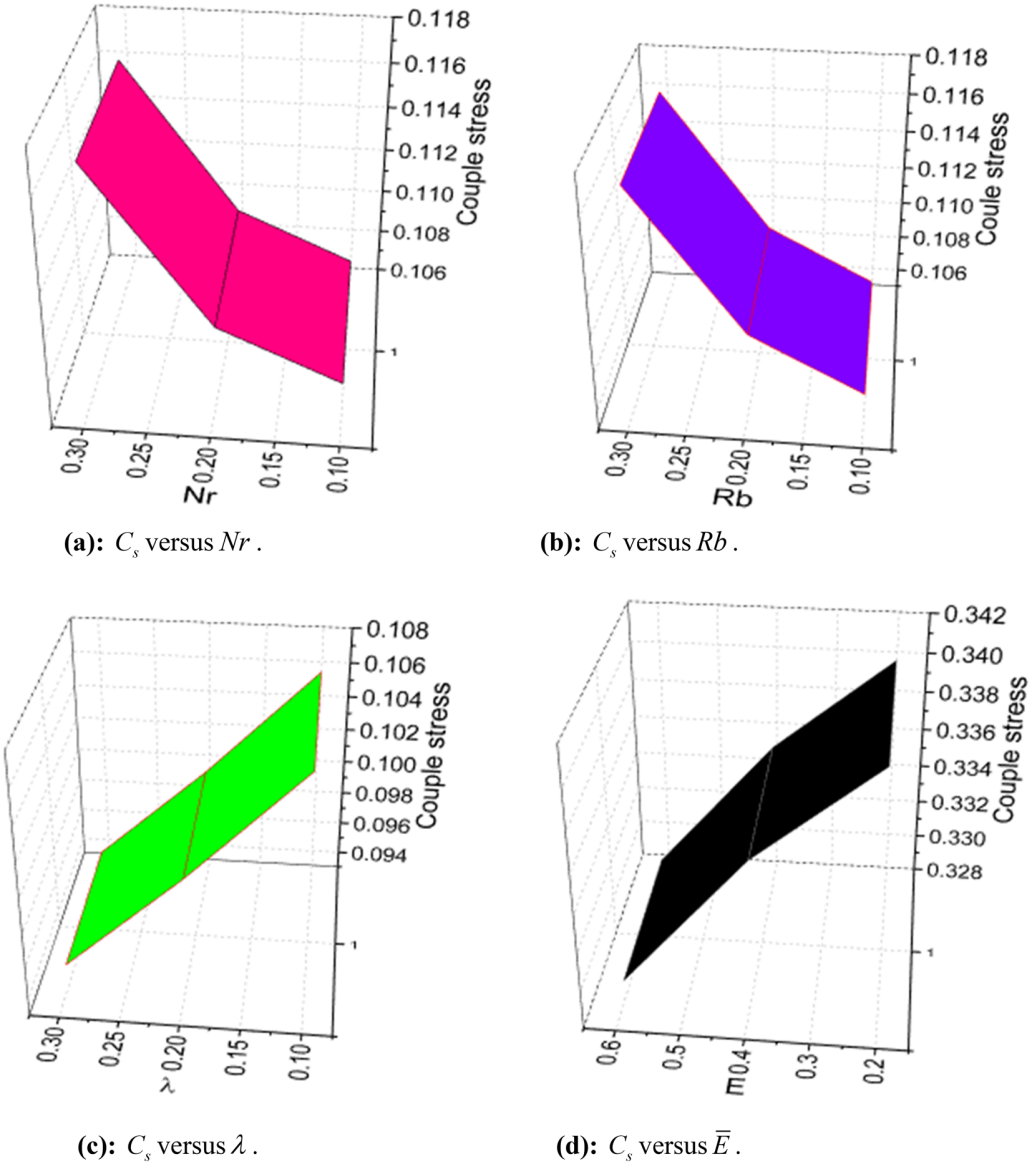
**(a)** $C_{s}$ versus $Nr$. **(b)** $C_{s}$ versus $Rb$. **(c)** $C_{s}$ versus $\lambda$. **(d)** $C_{s}$ versus $\overline{E}$.

**Figure 14:**
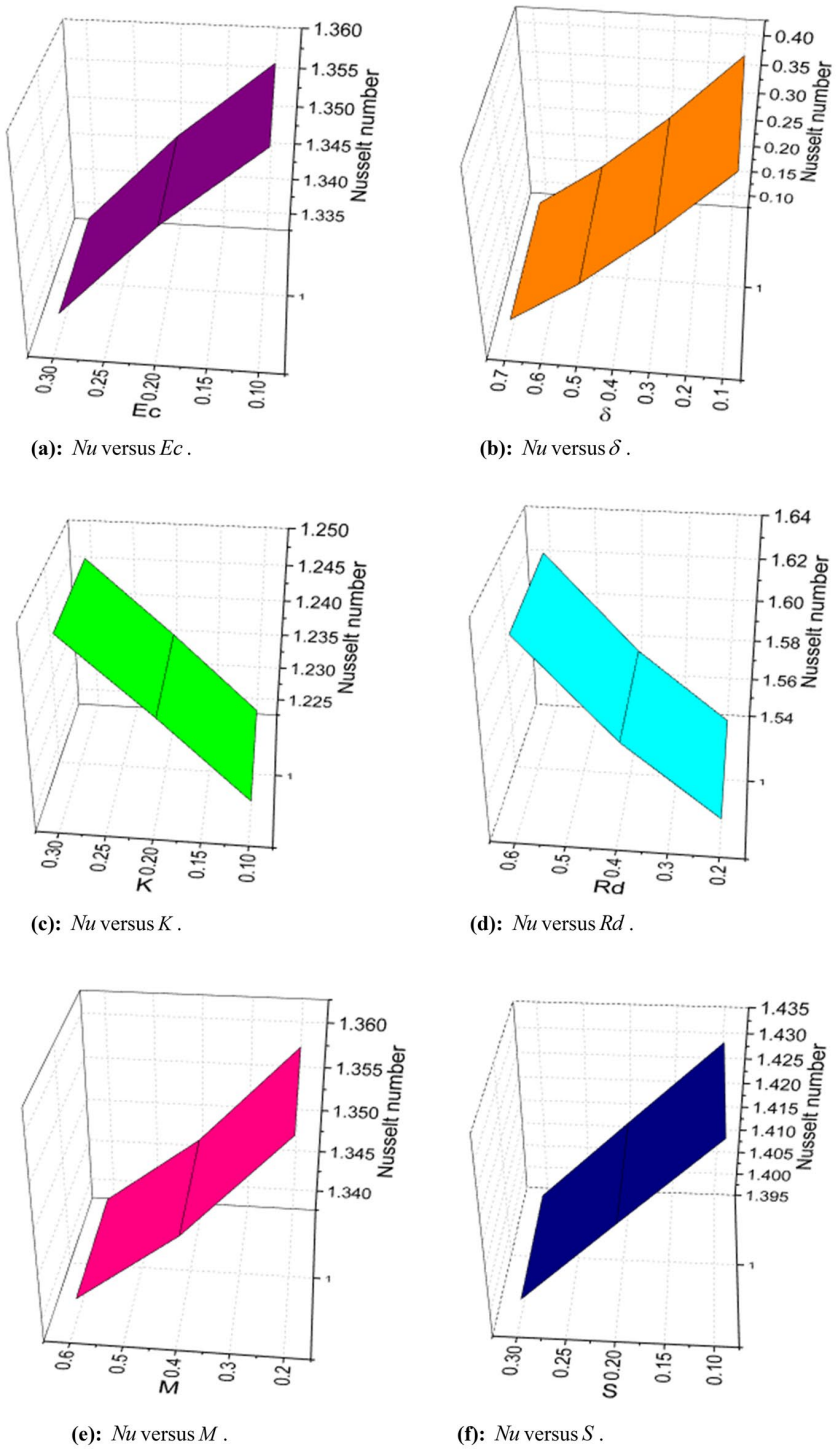
**(a)** $Nu$ versus $Ec$. **(b)** $Nu$ versus $\delta$. **(c)** $Nu$ versus $K$. **(d)** $Nu$ versus $Rd$. **(e)** $Nu$ versus $M$. **(f)** $Nu$ versus $S$.

**Figure 15:**
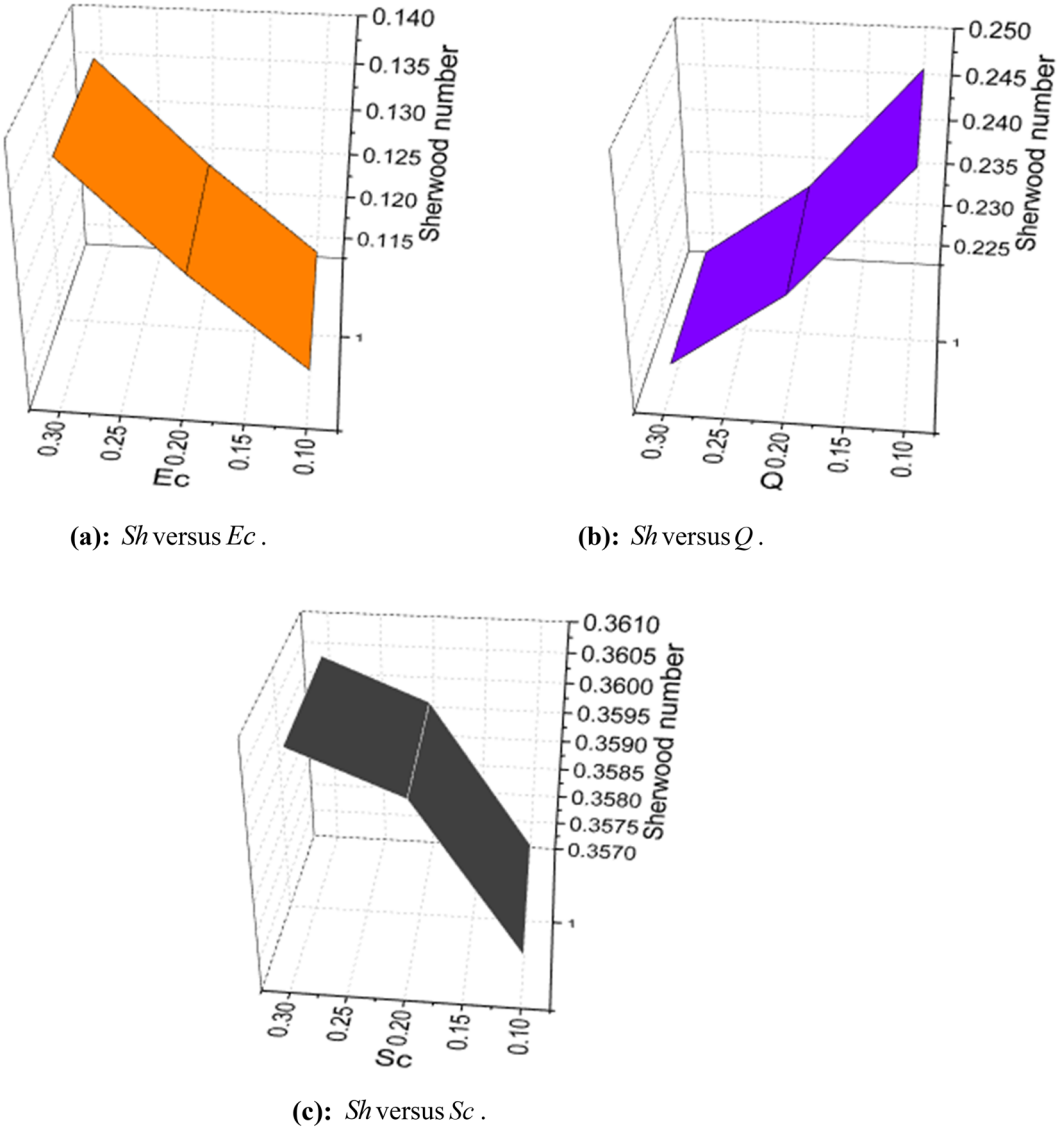
**(a)** $Sh$ versus $Ec$. **(b)** $Sh$ versus $Q$. **(b)** $Sh$ versus $Sc$.

**Figure 16:**
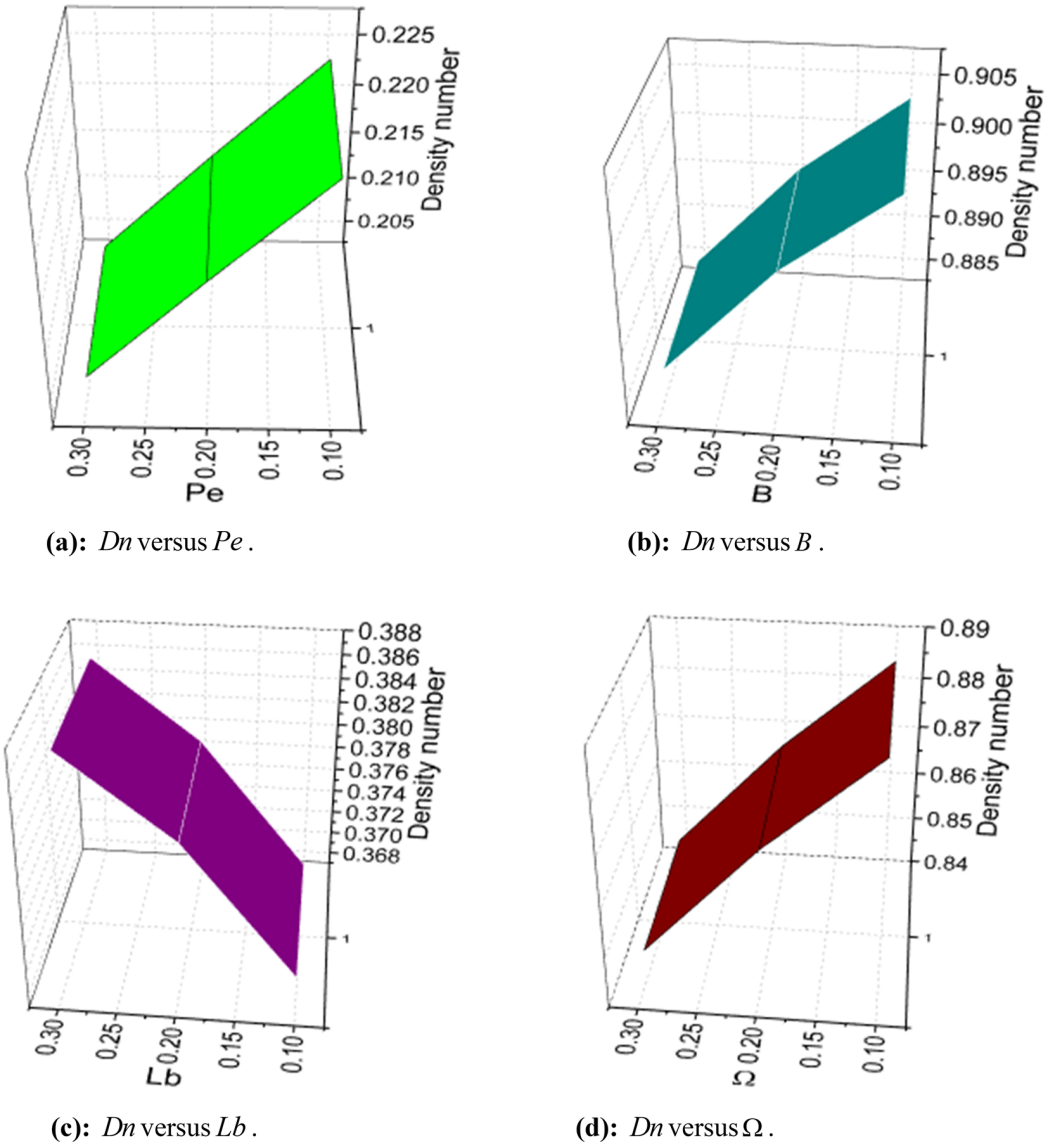
**(a)** $Dn$ versus $Pe$. **(b)** $Dn$ versus $B$. **(c)** $Dn$ versus $Lb$. **(d)** $Dn$ versus $\Omega$.

In this section, variations in the flow profiles of binary fluid due to embedded parameters defined in Table 3, are displayed with the help of Figures, and discussed in detail. Figure 3a shows the flow profiles of the modeled problem. Here, we have verified the boundary conditions for $f^{\prime}\left( \xi \right)$, $g\left( \xi \right)$, $\theta \left( \xi \right)$, $\phi \left( \xi \right)$, and $\chi \left( \xi \right)$. Figure 3b–f show the variation in $f^{\prime}\left( \xi \right)$, $g\left( \xi \right)$, $\theta \left( \xi \right)$, $\phi \left( \xi \right)$, and $\chi \left( \xi \right)$ due to $K$. Here, it is found that the increasing micropolar constant increases the velocity and microrotation functions (see Fig. 3b,c). Physically the greater change in micropolar constant is associated with the fluid viscosity. The higher change in micropolar constants diminishes the binary fluid viscosity due to which the velocity function heightens. Consequently, an increasing impact in velocity function due to micropolar constants is depicted (see Fig. 3b). A similar impact of micropolar constant against microrotation function is observed as shown in Fig. 3c. In addition, the binary fluid has maximum viscosity when the micropolar constant converges to zero (i.e.$K = 0$). The decreasing impact in temperature, concentration, and motile density functions due to micropolar constant is shown in Figs. 3d–f. Actually, the higher change in micropolar constants reduces the viscosity of the nanoparticles of due to which the temperature and concentration of the nanoparticles reduces. Thus, decreasing impacts in temperature and concentration functions are depicted. A similar impact of micropolar constant against motile density function is depicted (see Fig. 3f). Figures 4a–e) show the variation in $f^{\prime}\left( \xi \right)$, $g\left( \xi \right)$, $\theta \left( \xi \right)$, $\phi \left( \xi \right)$, and $\chi \left( \xi \right)$ due to $Nr$. A decreasing impact in velocity profile is depicted here. This impact is due to the buoyancy forces which lead the velocity function to decrease. However, the microrotation function increases from $0 \le Nr \le 1$ while a decreasing impact is depicted as $1 \le Nr < \infty$. Furthermore $\theta \left( \xi \right)$, $\phi \left( \xi \right)$, and $\chi \left( \xi \right)$ functions increase with greater $Nr$. Figure 5a–e show the variation in $f^{\prime}\left( \xi \right)$, $g\left( \xi \right)$, $\theta \left( \xi \right)$, $\phi \left( \xi \right)$, and $\chi \left( \xi \right)$ due to $Rb$. The decreasing impact of $Rb$ on $f^{\prime}\left( \xi \right)$ and $g\left( \xi \right)$ is depicted (see Fig. 5a, b). Physically, higher values of $Rb$ heighten the buoyancy forces which conclude the decreasing impact in $f^{\prime}\left( \xi \right)$. However, $g\left( \xi \right)$ increases from $0 \le Rb \le 1$ while a decreasing impact is depicted as $1 \le Rb < \infty$. Furthermore, $\theta \left( \xi \right)$, $\phi \left( \xi \right)$, and $\chi \left( \xi \right)$ increase with greater $Rb$. Figure 6a, b show the variation in $f^{\prime}\left( \xi \right)$ via $M$ in the absence and presence of $\overline{E}$. $f^{\prime}\left( \xi \right)$ reduces with greater $M$ in the absence of $\overline{E}$. The Lorenz Force which is greater with a larger magnetic field is obviously dependent on the magnetic field. The Lorenz force augments the frictional force in the absence of $\overline{E}$, acting as a retarding force that opposes the flow of binary fluid. In the presence of $\overline{E}$, the magnetic parameter has increasing impact on velocity profile (see Fig. 6b). Physically, $\overline{E}$ increases the body force which accelerates the flow velocity. Thus, an increasing conduct is observed here. A similar impact is depicted in^53^. Figures 6c,d show the variation in $g\left( \xi \right)$ via $M$ in the absence and presence of $\overline{E}$. In the absence of $\overline{E}$, a decreasing impact of $M$ on $g\left( \xi \right)$ is depicted (see Fig. 6c). However in the presence of $\overline{E}$, magnetic field parameter has dual impact on $g\left( \xi \right)$ (see Fig. 6d). Figure 7a, b display the variation in $f^{\prime}\left( \xi \right)$ and $g\left( \xi \right)$ via $\overline{E}$. The greater $\overline{E}$ increases the velocity function whereas the microrotation function reduces with greater electric field parameter. With larger values of $\overline{E}$, the velocity function boosts. Physically, the electric field acts as a decreasing force and lowering the fluid's frictional strength and thereby increasing the fluid velocity. However, electric field has the opposite effect on microrotation function. Figure 8a–c denote the variation in $\theta \left( \xi \right)$, $\phi \left( \xi \right)$, and $\chi \left( \xi \right)$ via thermal, mass, and motile density stratification parameters, respectively. Physically, the thermal stratification parameter increases the fluid density which results reduction in the temperature function. A decreasing impact of mass, and motile density stratification parameters are also depicted on concentration and motile density functions. Figure 9a–c show the variation in $\theta \left( \xi \right)$ due to $Rd$, $Ec$, and $\delta$ respectively. The influence of $Rd$ on $\theta \left( \xi \right)$ is displayed in Fig. 9a. The greater thermal radiation increases the temperature function. Physically, the greater thermal radiation means production of more heat to the fluid flow system. Thus, the greater thermal radiation parameter heightens the temperature function. Figure 9b displays the inspiration of $Ec$ on $\theta \left( \xi \right)$. The greater $Ec$ increases the temperature function. Physically, the increasing dissipation improves the thermal conductivity of the binary fluid which consequently enhances the temperature boundary layer thickness. Figure 9c shows the influence of $\delta$ on $\theta \left( \xi \right)$. The greater $\delta$ increases $\theta \left( \xi \right)$. Physically, increasing $\delta$ releases energy to the fluid flow which accordingly heightens the thermal function. Thus, $\theta \left( \xi \right)$ increases with greater $\delta$. Figure 10a–c represent the variation in $\phi \left( \xi \right)$ due to $\varpi$, $E$, and $Sc$ respectively. The impact of $\varpi$ on $\phi \left( \xi \right)$ is displayed in Fig. 10a. The greater $\varpi$ reduces $\phi \left( \xi \right)$. Physically, the increasing chemical reaction results the thickening in a concentration boundary layer. With greater $\omega$ the term $\varpi \left( {1 + \varepsilon \theta } \right)^{{\overline{n}}} \exp \left( {\frac{ - E}{{\left( {1 + \varepsilon \theta } \right)}}} \right)$ increases significantly. Thus, the greater chemical reaction parameter declines the concentration function. Figure 10b shows the impact of $E$ on $\phi \left( \xi \right)$. $\phi \left( \xi \right)$ increases with greater $E$. Actually, the greater activation energy heightens the thickness of mass transport boundary layer which consequently increases $\phi \left( \xi \right)$. Figure 10c specifies the deviation in $\phi \left( \xi \right)$ due to $Sc$. The greater $Sc$ reduces $\phi \left( \xi \right)$. The Schmidt number has opposite relation with mass diffusivity which consequently reduces $\phi \left( \xi \right)$. Figure 11a,b show the variation in $\chi \left( \xi \right)$ due to $Pe$, and $Lb$ respectively. The higher $Pe$ reduces $\chi \left( \xi \right)$. Physically, the diffusivity of microorganisms declines with greater $Pe$ which consequently reduces $\chi \left( \xi \right)$. Figure 11b shows the variation in $\chi \left( \xi \right)$ due to $Lb$. It is found that $\chi \left( \xi \right)$ reduces with greater $Lb$. Figures 12a–d specify the impacts of $Nr$, $Rb$, $\lambda$, and $\overline{E}$ on skin friction. The increasing $Nr$, $Rb$ and $\overline{E}$ increases the skin friction whereas the increasing $\lambda$ reduces the skin friction. Figures 13a,d specify the impacts of $Nr$, $Rb$, $\lambda$, and $\overline{E}$ on couple stress. The increasing $Nr$ and $Rb$ increases the couple stress whereas the increasing $\lambda$ and $\overline{E}$ reduces the couple stress. Figures 14a–f specify the impacts of $Ec$, $\delta$, $K$, $Rd$, $M$ and $S$ on Nusselt number. The increasing $K$, $Rd$, and $Ec$ increases the Nusselt number whereas the increasing $\delta$, $S$ and $M$ reduces the Nusselt number. Figure 15a–c specify the impacts of $Ec$, $Sc$, and $Q$ on Sherwood number. The increasing $Ec$ and $Sc$ increases the Sherwood number whereas the increasing $Q$ reduces the Sherwood number. Figure 16a–d specify the impacts of $Pe$, $B$, $Lb$ and $\Omega$ on density number. The greater $Lb$ increases the density number, while $Pe$, $B$ and $\Omega$ has reverse impacts on density number. Tables 4, 5, 6, 7 and 8 show the numerical values of $- f^{\prime\prime}\left( 0 \right)$, $g^{\prime}\left( 0 \right)$, $\theta^{\prime}\left( 0 \right)$, $\phi^{\prime}\left( 0 \right)$, and $\chi^{\prime}\left( 0 \right)$ via different embedded parameters. The outcomes are discussed in Figs. 12, 13, 14, 15, 16.

**Table 3:** Embedded parameters and their default values.

Parameter	Default value	Parameter	Default value	Parameter	Default value
$K$	1.0	$Nr$	0.8	$Ec$	0.4
$\alpha_{1}$	0.5	$Rb$	0.7	$\delta$	0.6
$M$	0.5	$Rd$	0.3	$Sc$	0.5
$\overline{E}$	0.2	$\Pr$	1.0	$\varpi$	0.3
$\lambda$	0.9	$S$	0.2	$E$	0.1
$Q$	0.2	$Lb$	0.7	$\Omega$	0.2
$\varepsilon$	0.1	$Pe$	0.4	$B$	0.2

**Table 4:** Numerical values of $- f^{\prime\prime}\left( 0 \right)$ via different embedded parameters.

$\lambda$	$Nr$	$Rb$	$\overline{E}$	$- f^{\prime\prime}\left( 0 \right)$
0.1	0.1	0.1	0.1	1.076865
0.2	0.1	0.1	0.1	1.038653
0.3	0.1	0.1	0.1	0.986367
0.1	0.2	0.1	0.1	1.084852
0.1	0.4	0.1	0.1	1.106473
0.1	0.6	0.1	0.1	1.129064
0.1	0.1	0.2	0.1	1.079995
0.1	0.1	0.4	0.1	1.087640
0.1	0.1	0.6	0.1	1.099853
0.1	0.1	0.1	0.2	1.346806
0.1	0.1	0.1	0.4	1.357537
0.1	0.1	0.1	0.6	1.368079

**Table 5:** Numerical values of $g^{\prime}\left( 0 \right)$ via different embedded parameters.

$\lambda$	$Nr$	$Rb$	$\overline{E}$	$g^{\prime}\left( 0 \right)$
0.1	0.1	0.1	0.1	0.106432
0.2	0.1	0.1	0.1	0.100175
0.3	0.1	0.1	0.1	0.094562
0.1	0.2	0.1	0.1	0.107527
0.1	0.4	0.1	0.1	0.109753
0.1	0.6	0.1	0.1	0.116474
0.1	0.1	0.2	0.1	0.106436
0.1	0.1	0.4	0.1	0.109292
0.1	0.1	0.6	0.1	0.116468
0.1	0.1	0.1	0.2	0.340543
0.1	0.1	0.1	0.4	0.335789
0.1	0.1	0.1	0.6	0.329064

**Table 6:** Numerical values of $\theta^{\prime}\left( 0 \right)$ via different embedded parameters.

$K$	$M$	$Rd$	$S$	$\delta$	$Ec$	$\theta^{\prime}\left( 0 \right)$
0.1	0.1	0.1	0.1	0.1	0.1	1.225757
0.2	0.1	0.1	0.1	0.1	0.1	1.236580
0.3	0.1	0.1	0.1	0.1	0.1	1.246725
0.1	0.2	0.1	0.1	0.1	0.1	1.358975
0.1	0.4	0.1	0.1	0.1	0.1	1.347853
0.1	0.6	0.1	0.1	0.1	0.1	1.339841
0.1	0.1	0.2	0.1	0.1	0.1	1.546853
0.1	0.1	0.4	0.1	0.1	0.1	1.580637
0.1	0.1	0.6	0.1	0.1	0.1	1.625721
0.1	0.1	0.1	0.1	0.1	0.1	1.431547
0.1	0.1	0.1	0.2	0.1	0.1	1.414789
0.1	0.1	0.1	0.3	0.1	0.1	1.397825
0.1	0.1	0.1	0.1	0.1	0.1	1.653368
0.1	0.1	0.1	0.1	0.2	0.1	1.636795
0.1	0.1	0.1	0.1	0.3	0.1	1.613404
0.1	0.1	0.1	0.1	0.1	0.1	1.357437
0.1	0.1	0.1	0.1	0.1	0.2	1.347533
0.1	0.1	0.1	0.1	0.1	0.3	1.335367

**Table 7:** Numerical values of $\phi^{\prime}\left( 0 \right)$ via different embedded parameters.

$Sc$	$Q$	$Ec$	$\phi^{\prime}\left( 0 \right)$
0.1	0.1	0.1	0.357446
0.2	0.1	0.1	0.359881
0.3	0.1	0.1	0.360563
0.1	0.1	0.1	0.247463
0.1	0.2	0.1	0.233853
0.1	0.3	0.1	0.225164
0.1	0.1	0.1	0.115788
0.1	0.1	0.2	0.125367
0.1	0.1	0.3	0.136352

**Table 8:** Numerical values of $\chi^{\prime}\left( 0 \right)$ via different embedded parameters.

$Lb$	$Pe$	$B$	$\Omega$	$\chi^{\prime}\left( 0 \right)$
0.1	0.1	0.1	0.1	0.368757
0.2	0.1	0.1	0.1	0.379642
0.3	0.1	0.1	0.1	0.386325
0.1	0.1	0.1	0.1	0.224678
0.1	0.2	0.1	0.1	0.214795
0.1	0.3	0.1	0.1	0.204526
0.1	0.1	0.1	0.1	0.904324
0.1	0.1	0.2	0.1	0.896537
0.1	0.1	0.3	0.1	0.885735
0.1	0.1	0.1	0.1	0.886474
0.1	0.1	0.1	0.2	0.868522
0.1	0.1	0.1	0.3	0.846723

## Conclusion

This analysis has been performed for electrically conducting MHD binary fluid containing nanoparticles and gyrotactic microorganisms through a stratified stretching sheet. The present analysis has been performed in the presence of electric and magnetic fields. An analytical scheme called HAM has been dedicated for the solution of the flow problem. The present analysis is compared with previously published results and has found a great agreement. The final comments are listed as:

1. The presence and absence of an electric field has affected the binary fluid velocities due to the magnetic field.
2. The velocity and microrotation functions have escalated, while the temperature, concentration, and motile density functions have reduced via microrotation constant.
3. The buoyancy parameter and bioconvection Rayleigh number have reduced the velocity function, while a dual impression of buoyancy parameter and bioconvection Rayleigh number on microrotation function are depicted.
4. The thermal, mass, and density stratification parameters have reduced the temperature, concentration, and motile density functions.
5. The greater chemical reaction parameter and Schmidt number have reduced the concentration function while an opposite behavior is observed via activation energy parameter.
6. The higher bioconvection Peclet and Lewis number have reduced the motile density function.
7. The increasing buoyancy ratio parameter, bioconvection Rayleigh number, and electric parameter have increased the skin friction, whereas the increasing mixed convection parameter have reduced the skin friction.
8. The increasing buoyancy ratio parameter and bioconvection Rayleigh number have increased the coupe stress, whereas the increasing mixed convection parameter and electric parameter have reduced the couple stress.

## Abbreviations

$$ {b}_{1} $$ , $$ {b}_{2} $$ , $$ {d}_{1} $$ , $$ {d}_{2} $$ , $$ {e}_{1} $$ , $$ {e}_{2} $$: Dimensionless constants
$B$: Motile density stratification parameter
$$ {B}_{0}$$: Strength of magnetic field
$$\overset{\lower0.5em\hbox{$\smash{\scriptscriptstyle\frown}$}}{b}_{c}$$: Chemotaxis constant
$$ {C}$$: Concentration
$$ {c}_{p}$$: Heat capacitance
$$ {B} $$: Diffusion constant
$$ {E}_{0}$$: Electric field
$E$: Activation energy parameter
$\overline{E}$: Electric field parameter
$$\overset{\lower0.5em\hbox{$\smash{\scriptscriptstyle\frown}$}}{E} a$$: Activation energy
$$Ec$$: Eckert number
$$\overset{\lower0.5em\hbox{$\smash{\scriptscriptstyle\frown}$}}{g}^{*}$$: Gravity
$j$: Micro-inertia density
$$K$$: Micropolar constant
$$\overset{\lower0.5em\hbox{$\smash{\scriptscriptstyle\frown}$}}{k}_{f}$$: Vortex viscosity
$$\overset{\lower0.5em\hbox{$\smash{\scriptscriptstyle\frown}$}}{k} r$$: Chemical reaction constant
$Lb$: Bioconvection Lewis number
$$M$$: Magnetic parameter
$$\overset{\lower0.5em\hbox{$\smash{\scriptscriptstyle\frown}$}}{n}$$: Concentration of the microorganisms
$$\overset{\lower0.5em\hbox{$\smash{\scriptscriptstyle\frown}$}}{N}$$: Angular velocity
$Nr$: Buoyancy ratio parameter
$Pe$: Bioconvection Peclet number
$$\Pr$$: Prandtl number
$Q$: Mass stratification parameter
$$\overset{\lower0.5em\hbox{$\smash{\scriptscriptstyle\frown}$}}{q}_{r}$$: Radiative heat flux
$Rb$: Bioconvection Rayleigh number
$$Rd$$: Thermal radiation
$S$: Thermal stratification parameter
$$Sc$$: Schmidt number
$$\overset{\lower0.5em\hbox{$\smash{\scriptscriptstyle\frown}$}}{T}$$: Temperature
$$\overset{\lower0.5em\hbox{$\smash{\scriptscriptstyle\frown}$}}{u}_{w}$$: Stretching velocity
$$\overset{\lower0.5em\hbox{$\smash{\scriptscriptstyle\frown}$}}{W}_{c}$$: Swimming cell speed
$$\overset{\lower0.5em\hbox{$\smash{\scriptscriptstyle\frown}$}}{x}$$,$$\overset{\lower0.5em\hbox{$\smash{\scriptscriptstyle\frown}$}}{y}$$: Coordinates

## Greek letters

$\Omega$: Microorganisms concentration difference parameter
$\varepsilon$: Temperature difference parameter
$\varpi$: Reaction rate parameter
$$\overset{\lower0.5em\hbox{$\smash{\scriptscriptstyle\frown}$}}{\kappa }$$: Boltzmann constant
$\delta$: Heat generation parameter
$$\overset{\lower0.5em\hbox{$\smash{\scriptscriptstyle\frown}$}}{\rho }_{f}$$: Density
$$\overset{\lower0.5em\hbox{$\smash{\scriptscriptstyle\frown}$}}{\sigma }$$: Electrical conductivity
$$\overset{\lower0.5em\hbox{$\smash{\scriptscriptstyle\frown}$}}{\nu }_{f}$$: Kinematic viscosity
$$\overset{\lower0.5em\hbox{$\smash{\scriptscriptstyle\frown}$}}{\beta }^{*}$$: Coefficient of volume expansion
$$\overset{\lower0.5em\hbox{$\smash{\scriptscriptstyle\frown}$}}{\alpha }_{f}$$: Thermal diffusivity
$$\overset{\lower0.5em\hbox{$\smash{\scriptscriptstyle\frown}$}}{\alpha }$$: Non-dimensionless viscoelastic parameter
$\alpha_{1}$: Dimensionless viscoelastic parameter
$$\overset{\lower0.5em\hbox{$\smash{\scriptscriptstyle\frown}$}}{\gamma }_{f}$$: Spin gradient viscosity
$\lambda$: Mixed convection parameter

## Subscripts

$$f$$: Nanofluids
$$p$$: Particles
$$m$$: Microorganisms
$$0$$: Reference
$$w$$: Surface
$$\infty$$: Free stream
